# Supplementary material for: Factors influencing decisions about whether to participate in health research by people of diverse ethnic and cultural backgrounds: a realist review
Source: BMJ Open. 2022 May 18;12(5):e058380. doi: 10.1136/bmjopen-2021-058380 (PMC9121482; doi:10.1136/bmjopen-2021-058380)
Supplement: Supplementary data [file bmjopen-2021-058380supp001.pdf]

Scoping Searches

Medline Scoping Search

Key terms informed consent, under-served , under-represented, health research, biomedical research 2005-current, English only

Database: Ovid MEDLINE(R) <1946 to May Week 2 2020>
Search Strategy:
----
1 informed consent.mp. or Informed Consent/ (57316)
2 exp Telemedicine/ or under-served.mp. or exp Vulnerable Populations/ (38247)
3 under-representation.mp. (756)
4 1 and 2 and 3 (0)
5 1 and 2 (820)
6 health research.mp. or exp Biomedical Research/ (277290)
7 5 and 6 (373)
8 limit 7 to (english language and yr="2005 -Current") (181)

****

Web of Science search May 21 2020

Key words : informed consent and under represented

Limits : English, 2005-current

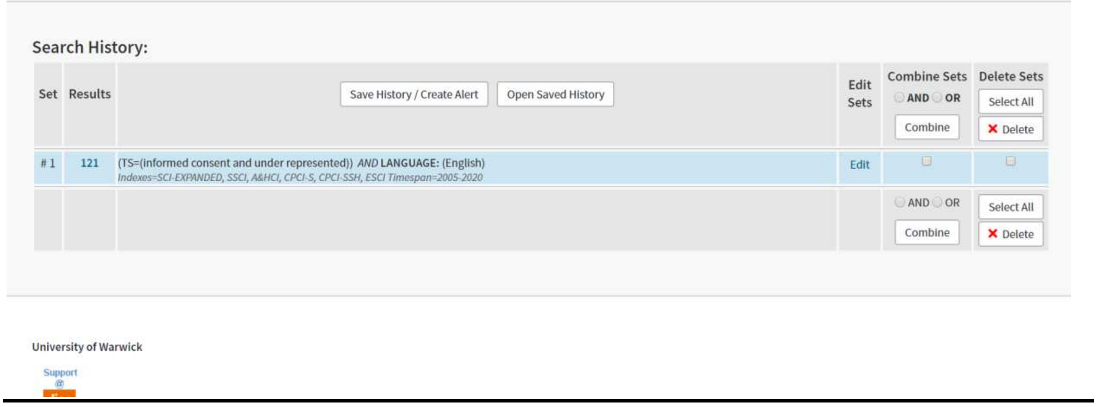

Database : UCL IRIS
Keywords : informed consent, under-served, health research, under represented

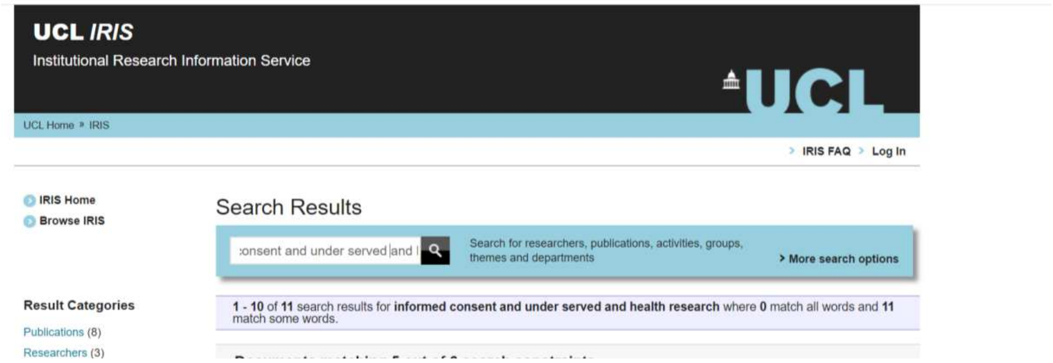

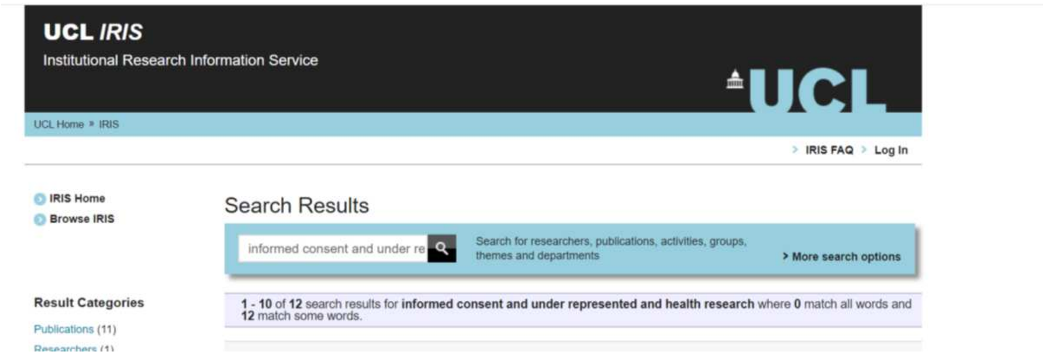

HRA (Health Research Authority)

Keywords: informed consent, underrepresented, health research
