# Supplementary material for: Factors influencing decisions about whether to participate in health research by people of diverse ethnic and cultural backgrounds: a realist review
Source: BMJ Open. 2022 May 18;12(5):e058380. doi: 10.1136/bmjopen-2021-058380 (PMC9121482; doi:10.1136/bmjopen-2021-058380)
Supplement: Supplementary data [file bmjopen-2021-058380supp002.pdf]

EMBASE SEARCH

Database: EMBASE <1947 to 2020 Week 20>

Search Strategy:

----

1 informed consent.mp. or exp informed consent/ (128719)

2 health research.mp. or exp medical research/ (433296)

3 1 and 2 (9419)

4 under served.mp. (587)

5 vulnerable populations.mp. or vulnerable population/ (20469)

6 under-served.mp. (587)

7 4 and 5 and 6 (27)

8 4 or 5 or 6 (21029)

9 3 and 8 (218)

10 limit 9 to english language (211)

****

Medline Search 1

Database: Ovid MEDLINE(R) <1946 to May Week 1 2020>

Search Strategy:

----

1 informed consent.mp. or exp Informed Consent/ (62033)

2 under served.mp. (312)

3 Health Services Research/ or health research.mp. or Biomedical Research/ (118031)

4 1 and 3 (2832)

5 under-served.mp. or exp Vulnerable Populations/ (10894)

6 4 and 5 (107)

****

Web of Science

TOPIC: (informed consent and under represented and health research)
Refined by: [excluding] PUBLICATION YEARS=(2004 OR 2003 OR 1993)

Timespan=All years. Indexes=SCI-EXPANDED, SSCI, A&HCI, CPCI-S, CPCI-SSH, ESCI.

"Title","Authors","Corporate Authors","Editors","Book Editors","Source Title","Publication
Date","Publication Year","Volume","Issue","Part Number","Supplement","Special
Issue","Beginning Page","Ending Page","Article Number","DOI","Conference
Title","Conference Date","Total Citations","Average per
Year","1900","1901","1902","1903","1904","1905","1906","1907","1908","1909","1910","1911","
1912","1913","1914","1915","1916","1917","1918","1919","1920","1921","1922","1923","1924",
"1925","1926","1927","1928","1929","1930","1931","1932","1933","1934","1935","1936","1937"
,"1938","1939","1940","1941","1942","1943","1944","1945","1946","1947","1948","1949","1950
","1951","1952","1953","1954","1955","1956","1957","1958","1959","1960","1961","1962","196
3","1964","1965","1966","1967","1968","1969","1970","1971","1972","1973","1974","1975","19
76","1977","1978","1979","1980","1981","1982","1983","1984","1985","1986","1987","1988","1
989","1990","1991","1992","1993","1994","1995","1996","1997","1998","1999","2000","2001","
2002","2003","2004","2005","2006","2007","2008","2009","2010","2011","2012","2013","2014",
"2015","2016","2017","2018","2019","2020"

Database: APA PsycInfo <1806 to May Week 4 2020>

Search Strategy:

----

1 (under-served or underserved).mp. [mp=title, abstract, heading word, table of contents, key concepts, original title, tests & measures, mesh] (5394)

2 health research.mp. (5729)

3 informed consent.mp. or exp Informed Consent/ (11199)

4 biomedical research.mp. (5165)

5 (under represented or under-represented).mp. [mp=title, abstract, heading word, table of contents, key concepts, original title, tests & measures, mesh] (1120)

6 underserved.mp. (5126)

7 under served.mp. (294)

8 vulnerable population.mp. (2420)

9 6 or 7 (5394)

10 informed consent.mp. or exp Informed Consent/ (11199)

11 decision making.mp. or exp Decision Making/ (167677)

12 research.mp. or exp Experimentation/ (1223074)

13 10 or 11 or 12 (1339577)

14 9 and 13 (2287)

15 1 or 5 or 6 or 7 (6498)

16 2 and 3 (168)

Iteration 1- search

Database: APA PsycInfo <1806 to May Week 3 2020>

Search Strategy:

----

1 INFORMED CONSENT.mp. or exp Informed Consent/ (11049)

2 HEALTH RESEARCH.mp. (5578)

3 exp Blacks/ or exp At Risk Populations/ or UNDER-SERVED.mp. or exp Disadvantaged/ (94625)

4 1 and 2 and 3 (7)

Database: Embase Classic+Embase <1947 to 2020 Week 20>

Search Strategy:

----

1 informed consent.mp. or informed consent/ (131165)

2 health research.mp. or medical research/ (234000)

3 Black.mp. or Black person/ (178998)

4 At risk populations.mp. (3605)

5 Under-served.mp. (601)

6 disadvantaged.mp. (15612)

7 1 and 2 and 3 (20)

Web of Science<br>17	TOPIC: (informed consent and minority ethnic groups and health research)<br>**Refined by:** [excluding] **PUBLICATION YEARS:** (2004 OR 2002)<br>*Indexes=SCI-EXPANDED, SSCI, A&HCI, CPCI-S, CPCI-SSH, ESCI Timespan=All years*

Database: Ovid MEDLINE(R) <1946 to June Week 1 2020>

Search Strategy:

----

1 informed consent.mp. or Informed Consent/ (57978)

2 health research.mp. (12581)

3 Black.mp. (102740)

4 at risk populations.mp. (2115)

5 under-served.mp. or Vulnerable Populations/ (10932)

6 disadvantaged.mp. or Vulnerable Populations/ (20499)

7 1 and 2 and 3 (1)

8 2 and 5 (101)

9 1 and 8 (22)

****

Iteration 2 – Searches

(Search 1)

Database: Embase Classic+Embase <1947 to 2020 Week 35>

Search Strategy:

----

1 institutional racism.mp. (185)

2 health research.mp. or medical research/ (234000)

3 informed consent.mp. or informed consent/ (131165)

4 1 and 2 (3)

5 limit 4 to yr="2005 -Current" (1)

****

Database: APA PsycInfo <1806 to July Week 1 2020>

Search Strategy:

----

1 INSTITUTIONAL RACISM.mp. (453)

2 health research.mp. (5729)

3 informed consent.mp. or exp Informed Consent/ (11199)

4 1 and 2 (2)

5 limit 2 to yr =”2005-Current” (0)

Database : Web of Science July Week 1

Results: 2
(from Web of Science Core Collection)
You searched for: **TOPIC:** (institutional racism and health research and informed consent)
Timespan: All years. **Indexes:** SCI-EXPANDED, SSCI, A&HCI, CPCI-S, CPCI-SSH, ESCI.

Database: Ovid MEDLINE(R) <1946 to July Week 1 2020>

Search Strategy:

----

1 institutional racism.mp. (115)

2 Health Services Research/ or health research.mp. or Biomedical Research/ (118339)

3 1 and 2 (9)

4 limit 3 to yr="2005 -Current" (5)

(Search 2)

Database: Embase Classic+Embase <1947 to 2020 Week 25>

Search Strategy:

----

1 cultural competency.mp. or cultural competence/ (7186)

2 health research.mp. or medical research/ (234000)

3 1 and 2 (213)

4 informed consent.mp. or informed consent/ (131165)

5 3 and 4 (9)

Database: Ovid MEDLINE(R) <1946 to July Week 1 2020>

Search Strategy:

----

1 cultural competency.mp. or Cultural Competency/ (6133)

2 Health Services Research/ or health research.mp. or Biomedical Research/ (118339)

3 1 and 2 (196)

4 informed consent.mp. or Informed Consent/ (58028)

5 3 and 4 (8)

Database: APA PsycInfo <1806 to July Week 1 2020>

Search Strategy:

----

1 cultural competency.mp. (2952)

2 health research.mp. (5729)

3 informed consent.mp. or exp Informed Consent/ (11199)

4 1 and 2 and 3 (1)

Results: 9

(from Web of Science Core Collection)

You searched for: **TOPIC:** (cultural competency and health research and informed consent)

Timespan: All years. **Indexes:** SCI-EXPANDED, SSCI, A&HCI, CPCI-S, CPCI-SSH, ESCI.
