# Supplementary material for: Factors influencing decisions about whether to participate in health research by people of diverse ethnic and cultural backgrounds: a realist review
Source: BMJ Open. 2022 May 18;12(5):e058380. doi: 10.1136/bmjopen-2021-058380 (PMC9121482; doi:10.1136/bmjopen-2021-058380)
Supplement: Supplementary data [file bmjopen-2021-058380supp003.pdf]

Inclusion/exclusion criteria

Inclusion criteria

- Quantitative and qualitative studies, grey literature, websites, stakeholder recommendations (e.g., reports, conference papers).
- The search will be limited to a 15-year publication period (2005-2020) due to the extent to which research infrastructure and research governance has changed over that period of time. Inclusion of review articles (published within the last 15 years) included evidence from earlier studies so that we were able to draw on earlier evidence where it was relevant. In addition to this, the evidence reviewed was sufficient to inform and develop programme theory which was the purpose of the review.
- All studies that report on experiences and decision-making involved in the informed consent process will be included.
- Any source of data that is deemed relevant by the stakeholder group.
- Literature that explores the concept and practice of informed consent.
- Only sources written in English will be included.

Exclusion criteria

- Studies or sources of data that are not relevant to the informed consent process in health research in relation to under-represented populations.
