# Supplementary material for: Factors influencing decisions about whether to participate in health research by people of diverse ethnic and cultural backgrounds: a realist review
Source: BMJ Open. 2022 May 18;12(5):e058380. doi: 10.1136/bmjopen-2021-058380 (PMC9121482; doi:10.1136/bmjopen-2021-058380)
Supplement: Supplementary data [file bmjopen-2021-058380supp004.pdf]

CMOC Long list

The CMOC's shaded blue are specific to people of diverse ethnic and cultural backgrounds in the UK. All other CMOC's include those from the initial programme theory.

CMOC	Context (How designed and delivered)	Mechanism (tailored, how info provided, delivered)	Outcome (decision-making)	Does it work?
**CMOC1**	**C2 Meso level**<br>**Organisations and people within them should deliver services with cultural competence**	**M1 Skilled , culturally competent researchers**<br>**Promotes positive and effective relationships with diverse communities . Researchers need to develop and employ sensitive skills to work with people from different cultural backgrounds**	**Meets the social, cultural, religious and linguistic needs of ethnic minority populations**	**yes**
**CMOC2**	**C1 Macro level**<br>**C2 Meso level**<br>**informed consent can be delivered in other formats apart from written**	**M4 Flexible processes, tailored information**<br>**oral, audio-recorded study information and verbal consent**	**promotes feelings of independence;**	**yes**
**CMOC3**	**C2 Meso level**<br>**communities advertising research**	**M5 Community involvement**<br>**shopkeepers and mosque leaders as advocates for research creates enthusiasm**	**influences decision to take part**	**yes**

**CMOC4**	**C2 Meso level appropriate matching of researcher to group being researched**	**M4 Flexible processes, tailored information consideration of the gender of the researcher due to religious reasons can affect engagement with discussion; matching ethnic background of researcher to ethnic background of participant**	**participant does not feel comfortable to discuss problems in some cases depending on gender of researcher, due to religious reasons; may prevent some participants from airing counter-cultural views**	**no**
**CMOC 5**	**M1 Macro level M2 Meso level**	**M4 Flexible processes, tailored information involving family, community members, or local health professionals to support shared decision making**	**shared decision making**	**yes**
**CMOC4**	**C2 Meso level ethnic matching**	**M4 Flexible processes, tailored information participant has some choice in which researcher they work with**	**effective and positive experience for potential participant, having a bearing on decision-making about taking part**	**yes**
**CMOC5**	**C1 Macro level C2 Meso level information provided only in English**	**M2 Removing/restricting choice considered institutionalised racism**	**excludes older ethnic minority populations and migrants**	**no**
**CMOC6**	**C1 Macro level C2 Meso level study design restricts access**	**M3 Inflexible process M2 Removing/Restricting choice**	**under-representation**	**no**

		eligibility criteria excludes certain populations		
**CMOC7**	**C2 Meso level**<br>**commitment to confidentiality**	**M1 Skilled, culturally competent researchers**<br>**researchers qualify confidentiality before obtaining consent, confirming comprehension**	**reduces fear of being reported to authorities (if migrant pop) and prevents decision of obedience to "community leaders"**	**yes**
**CMOC8**	**C1 Macro level**<br>**C3 Micro level**<br>**illiteracy, knowledge of health research, legal aspects**	**M3 Inflexible process**<br>**signed informed consent , lacking in assurance of confidentiality**	**feel undue pressure to participate , or refuse due to fear of a negative impact on immigration process**	**no**
**CMOC9**	**M1 Macro level**<br>**M2 Meso level**<br>**study design**	**M1 Skilled, culturally competent researchers**<br>**awareness of local protocol, legislation and culture; consideration of potential participants current situation in life; potential participant's awareness of the study; sensitive recruitment**	**culturally appropriate instruments , successful informed consent**	**yes**

**CMOC11**	**M3 Micro level<br>life situation of some populations e.g. migrant communities**	**M2 Removing/restricting choice<br>M3 Inflexible process<br>individual decision-making not possible due to family relationships**	**identification of key family/community member involved in decision making with improved**	**yes**
**CMOC12**	**M1 Macro level<br>M2 Meso level<br>design and delivery of study**	**M4 Flexible processes, tailored information<br>close connection to community - conversations are basis for informed consent , summarising information, explaining written information (this can be supported by traditional methods, witnessed consent, audio-recording, video-taping or illustrations)**	**consent given freely, respect for cultural differences, improves trust , is ethical and ensures dignity**	**yes**
**CMOC13**	**M1 Macro Level<br>M2 Meso level<br>wider health care system**	**M3 Inflexible processes<br>M2 Removing / restricting choice<br>policies of institutions<br>1. presumption that one size fits all<br>2. difference is acknowledged but needs are misrepresented**	**discriminatory outcomes (e.g. exclusion due to eligibility criteria); ignores needs of ethnically diverse populations; enforces inappropriate policy and responses by health professionals**	**no**

**CMOC14**	**M1 Macro level M2 Meso level primary health care**	**M2 Removing/restricting choice gaining access institutional racism used as a framework in healthcare**	**helps to understand discrimination and disadvantages minority ethnic groups experience**	**yes**
**CMOC15**	**M1 Macro level low-income countries**	**M2 Removing/restricting choice parental/guardian control**	**results in exclusion from taking part**	**no**
**CMOC 16**	**M1 Macro level M2 Meso level mature minors (often under-represented)**	**M4 Flexible processes, tailored information assessed for competence**	**meet competence threshold, providing valid informed consent**	**yes**
**CMOC 17**	**M1 Macro level health research sponsored by wealthy nations in low income countries**	**M3 Inflexible process Lengthy consent documentation, not tailored to those with low literacy levels**	**Inducement , poor understanding of study information, questionable validity of informed consent , exploitation**	**no**
**CMOC 18**	**M1 Macro level health research in resource-poor settings**	**M1 Skilled, culturally competent researchers delivered by trained researchers in obtaining valid consent from minors, with age-appropriate study information**	**free choice to participate**	**unclear**
**CMOC19**	**M1 Macro level regulations protect vulnerable populations from taking part**	**M2 Removing /restricting choice information not provided**	**removes freedom of choice**	**no**

**CMOC 20**	**M2 Meso level researcher matching**	**M1 Skilled, culturally competent researchers researchers from similar backgrounds who speak the language , understand cultural circumstances**	**creates trust**	**unclea r**
**CMOC21**	**M1 Macro level informed consent in low-income countries**	**M1 Skilled, culturally competent researchers fieldworker trained in communication skills and utilising local language to share information, in bite-size chunks**	**benefits potential participants whom are educated as knowledge is better**	**unclea r**
**CMOC22**	**M2 Meso level research protocol design**	**M3 Inflexible process M2 Removing/restricti ng choice complex forms and communication barriers, lack of social presence in the community, lack of diversity amongst researchers**	**results in exclusion, experience of mistrust, feel exploited and that academic institutions are not committed to ethnic minority communities, suspicion of formal transaction**	**no**
**CMOC23**	**M2 Meso level community participation and ethical review boards willingness to adapt existing process**	**M4 Flexible processes, tailored information adaptation of resources, consent in different formats (incl multimedia),**	**creates trust, better conditions , assessment of comprehension & acceptability, cultural sensitivity promotes access, community**	**unclea r**

			**relationship building**	
**CMOC24**	**M2 Meso level<br>design of study for offenders with serious mental health problems**	**M4 Flexible processes, tailored information<br>careful description of study, how it is communicated and by whom, in conjunction with dialogue-type counselling techniques , for patients whom are deemed to be able to make a competent decision**	**reduces coercion and confusion of role as a patient as opposed to a potential participant**	**unclear**
**CMOC25**	**M1 Macro level<br>ethical guidelines for informed consent**	**M2 Removing choice/restricting choice<br>restrict operational flexibility**	**lack of cultural sensitivity and appreciation of family and cultural dynamics that affect decision-making**	**no**
**CMOC26**	**M1 Macro level<br>many African countries**	**M1 Skilled, culturally competent researchers<br>culturally appropriate delivery, taking into consideration social and cultural conventions, familiarity with local context of research**	**improved acceptability of the decision-making process**	**yes**
**CMOC27**	**M2 Meso level<br>M1 Micro level**	**M2 Removing/restricting choice**	**prevents inclusion, and**	**no**

	**research with patients with cognitive decline**	**family members, clinicians and care facility staff acting as gatekeepers**	**freedom of choice**	
**CMOC28**	**M2 Meso Level<br>M1 Micro level<br>research with patients with cognitive decline e.g. dementia**	**M4 Flexible processes, tailored information capacity assessment processes such as a MMSE score, as a way of determining capacity to consent and communication strategies tailored to meet needs of those with dementia**	**enables person to make own decision , whilst is a continuous check of willingness**	**yes**
**CMOC29**	**M2 Meso level<br>M1 Micro level<br>research with people with intellectual disability**	**M4 Flexible processes, tailored information adapting study materials and communication strategies during the informed consent process**	**builds rapport and engagement with participant and enables them to participate in the consent process to the best of their ability**	**yes**
