# Supplementary material for: Factors influencing decisions about whether to participate in health research by people of diverse ethnic and cultural backgrounds: a realist review
Source: BMJ Open. 2022 May 18;12(5):e058380. doi: 10.1136/bmjopen-2021-058380 (PMC9121482; doi:10.1136/bmjopen-2021-058380)
Supplement: Author's manuscript [file bmjopen-2021-058380.draft_revisions.pdf]

BMJ Open

BMJ Open is committed to open peer review. As part of this commitment we make the peer review history of every article we publish publicly available.

When an article is published we post the peer reviewers' comments and the authors' responses online. We also post the versions of the paper that were used during peer review. These are the versions that the peer review comments apply to.

The versions of the paper that follow are the versions that were submitted during the peer review process. They are not the versions of record or the final published versions. They should not be cited or distributed as the published version of this manuscript.

BMJ Open is an open access journal and the full, final, typeset and author-corrected version of record of the manuscript is available on our site with no access controls, subscription charges or pay-per-view fees (http://bmjopen.bmj.com).

If you have any questions on BMJ Open's open peer review process please email info.bmjopen@bmj.com

BMJ Open

Factors influencing decisions about whether to participate in health research by people of diverse ethnic and cultural backgrounds: a realist review.

Journal:	*BMJ Open*
Manuscript ID	bmjopen-2021-058380
Article Type:	Original research
Date Submitted by the Author:	23-Oct-2021

Complete List of Authors:	Hoverd, Eleanor; University of Warwick<br>Hawker-Bond, George; Oxford University, Nuffield Department of Clinical Neurosciences,<br>Staniszewska, Sophie; University of Warwick, Warwick Medical School<br>Dale, Jeremy; University of Warwick, Warwick Medical School

Keywords:	Health policy < HEALTH SERVICES ADMINISTRATION & MANAGEMENT, MEDICAL ETHICS, Clinical trials < THERAPEUTICS

SCHOLARONE™
Manuscripts

I, the Submitting Author has the right to grant and does grant on behalf of all authors of the Work (as defined in the below author licence), an exclusive licence and/or a non-exclusive licence for contributions from authors who are: i) UK Crown employees; ii) where BMJ has agreed a CC-BY licence shall apply, and/or iii) in accordance with the terms applicable for US Federal Government officers or employees acting as part of their official duties; on a worldwide, perpetual, irrevocable, royalty-free basis to BMJ Publishing Group Ltd ("BMJ") its licensees and where the relevant Journal is co-owned by BMJ to the co-owners of the Journal, to publish the Work in this journal and any other BMJ products and to exploit all rights, as set out in our licence.

The Submitting Author accepts and understands that any supply made under these terms is made by BMJ to the Submitting Author unless you are acting as an employee on behalf of your employer or a postgraduate student of an affiliated institution which is paying any applicable article publishing charge ("APC") for Open Access articles. Where the Submitting Author wishes to make the Work available on an Open Access basis (and intends to pay the relevant APC), the terms of reuse of such Open Access shall be governed by a Creative Commons licence – details of these licences and which Creative Commons licence will apply to this Work are set out in our licence referred to above.

Other than as permitted in any relevant BMJ Author's Self Archiving Policies, I confirm this Work has not been accepted for publication elsewhere, is not being considered for publication elsewhere and does not duplicate material already published. I confirm all authors consent to publication of this Work and authorise the granting of this licence.

For peer review only

TITLE

Factors influencing decisions about whether to participate in health research by people of diverse ethnic and cultural backgrounds: a realist review.

Corresponding Author: Mrs. Eleanor Hoverd, NIHR Clinical Research Network West Midlands, Room A117, Warwick Medical School, Warwick University, Gibbet Hill Rd, Coventry CV4 7AL, England eleanor.hoverd@nihr.ac.uk

Co-authors:
Dr.George Hawker-Bond, MChem (Hons) Chemistry, MBChB in Medicine and Surgery, Nuffield Department of Clinical Neurosciences, University of Oxford, Oxford, England

Professor Sophie Staniszewska, Warwick Research in Nursing, Warwick Medical School, Warwick University, Coventry, England

Professor Jeremy Dale, Unit of Academic Primary Care, Warwick Medical School, Warwick University, Coventry, England

Word count 4,007

For peer review only - http://bmjopen.bmj.com/site/about/guidelines.xhtml

ABSTRACT

Objective To develop and refine a programme theory that explains factors that influence decisions to take part in health research by people of diverse ethnic and cultural backgrounds.

Design Realist review following a sequence of five steps: 1) Scoping search and identification of programme theory 2) Evidence searching 3) Critical appraisal and data extraction 4) Organisation of evidence 5) Refinement of programme theory.

Eligibility criteria Documents (including peer-reviewed articles, grey literature, websites, reports, and conference papers) either full text, or a section of relevance to the over-arching research question were included.

Analysis Literature was searched iteratively to test theories that explain how and why a decision to participate in health research is made, based on understanding and experience of informed consent. Key stakeholders helped to refine and finalise programme theory. Data from eligible documents was extracted to build understanding of the factors that influence decision-making. Data was mapped to create a data matrix according to Context (C), Mechanism (M), Outcome (O), Configurations (C) (CMOCs) for the process of informed consent, to aid interpretation and produce a final programme theory.

Results 566 documents were screened and 71 included. The final programme theory included 14 CMOCs on processes influencing decisions to take part in research. Key findings indicate the type of infrastructure required, e.g. resources, services and policies, to support inclusion in health research, with a greater need to increase the social presence of researchers within communities, improve cultural competency of individuals and organisations, reduce the complexity of participant information , and provide additional resources to support adaptive processes and shared-decision making.

For peer review only - http://bmjopen.bmj.com/site/about/guidelines.xhtml

Conclusion The review describes factors that influence the decision to take part in health research with people of diverse ethnic and cultural backgrounds, identifying what works and what does not. It recommends actions for developing and adapting health research systems.

For peer review only

ARTICLE SUMMARY

Strengths and limitations of this study

· Using realist methods to explore the contexts and mechanisms of the complexities affecting the informed consent process in health research, for people of diverse ethnic and cultural populations, allowed new insight to emerge.

· There is a paucity of evidence around the informed consent process in health research with people of diverse ethnic and cultural backgrounds, limiting the evidence from which to extract data from.

· Relevance and rigour were increased through the involvement of National Institute for Health Research (NIHR) (Public) Research Champions, healthcare professionals and clinical academics in refining the programme theory.

· Owing to the COVID-19 pandemic, stakeholder involvement in the programme was conducted remotely, enabling access from a wider geographical spread of stakeholders.

·Whilst the review was undertaken in the context of the UK health care system, the programme theory is likely to have wider applicability.

Keywords: realist review, informed consent, under-served, ethnic minority, health research

For peer review only - http://bmjopen.bmj.com/site/about/guidelines.xhtml

INTRODUCTION

Background

The informed consent process in health research aims to provide patients and the public with the information they require to make a voluntary decision about participation. People taking part in health research do not represent the diversity of our population[1]. People who have poorer health outcomes, and have greater needs are currently under-served by the way in which health research is accessed and delivered[1]. It has been argued that the informed consent process may be contributing to inequalities in health research through a lack of flexibility in the process[2-10] . In May 2019, the International Council for Harmonisation (ICH)[11]-drafted the *General Considerations for Clinical Studies E8 (R1)* suggesting that a "one size fits all approach" to studies should be avoided.

Lack of diversity in health research poses a serious challenge both within the UK and globally, around how to address and develop a more inclusive health research system so that those whom are under-served can benefit. Hence, it is important to unravel the factors that may affect the decision to take part in health research by those whom are under-served. In this study, we undertook a realist review to produce a programme theory to explain the informed consent process, and hence provide actionable recommendations for policymakers and health researchers to inform the development of inclusive health research.

The over-arching research question for this review is: What contextual factors influence the decision to take part in health research in under-served populations? The term under-served is preferred when referring to groups, or communities who are underrepresented in health research e.g. marginalised groups, and is also used to describe geographical locations such as rural or low-income countries, although a single definition is not found within the literature[1].

For peer review only - http://bmjopen.bmj.com/site/about/guidelines.xhtml

Objectives were as follows:

1. To undertake a realist review to identify the contexts and mechanisms that affect the informed consent process and the decision to participate in health research in under-served populations .
2. To draw on the review's programme theory to develop guidance that addresses barriers to participating in health research in under-served populations.

METHODS

The review process followed the steps as laid out in the protocol[12] and was conducted and reported in accordance with RAMESES guidance and publication standards, as well as providing a rationale for selecting this approach[13].

Patient Public Involvement

NIHR (Public) Research Champions were involved as stakeholders in this review[14]. Their involvement is discussed below (step 1).

Development of an initial programme theory (step 1)

In order to develop an initial rough programme theory, a scoping search (see online supplementary file 1 Scoping searches) was carried out based on search terms centred on the intervention under study (informed consent) with under-served populations in health research, to explore key literature in this field. Theoretical concepts and evidence that would contribute to explaining different outcomes; for example, positive (understood study information) or negative experiences (excluded from taking part). A theoretical concept map was created (figure 1) identifying higher-level, abstract theories which provided a basis to discuss some of the larger complexities of informed consent with the stakeholder group in combination with the empirical evidence from the scoping search, as a way of supporting, contradicting , or modifying the programme theory and to refine the purpose of the review[15].

For peer review only - http://bmjopen.bmj.com/site/about/guidelines.xhtml

The initial scoping search resulted in 16 documents selected based on relevance to the over-arching research question[16-31]. These were examined, with codes applied to label Context, Mechanisms, Outcomes Configurations (CMOCs), concepts and theories, which were helpful in building the initial programme theory. These CMOCs were then collated, a long list of CMOCs drawn-up and then developed into a diagram, to create a visualisation of the initial programme theory (figure 2). This enabled identification of key theories for further exploration.

Stakeholder involvement

Stakeholder meetings were organised to provide perspectives and expert opinions around issues relevant to building programme theory, insights into the ways in which social structure may influence the informed consent process, as well as identifying any pertinent documents of relevance[32]. Stakeholder input is described in the protocol[12] (see online supplementary file 2 Searches) .

Evidence searching (step 2)

Literature searches were conducted iteratively. The initial search used the databases EMBASE, Medline, Web of Science and Psych Info, in addition to hand-searching using Google and Google Scholar. The search strategy was refined for each database , aiming to provide a broad enough review for further building of programme theory.

Following the initial search, 468 citations were identified following removal of duplicates, with 54 citations remaining following 1st and 2nd level screening by EH and G H-B. As synthesis progressed, key theories were emerging around the informed consent process with people of diverse ethnic and cultural backgrounds, with most of literature from the US. Therefore, following discussion with stakeholders it was agreed synthesis would focus on a smaller number of key theories with additional searches required to test and refine

For peer review only - http://bmjopen.bmj.com/site/about/guidelines.xhtml

programme theory, focussing on a “*conceptually rich*”[33] sub-group of literature to produce recommendations relevant to the UK health research system. The reason for limiting the subsequent searches to test theories within the UK literature was because an inclusion policy is lacking, and with a free , publicly funded health care system, context and resources may differ in comparison to other geographical locations, thus affecting mechanisms and outcomes. A flow diagram of the iterative searching process is displayed in figure 3, based on RAMESES guidelines[13].

A key consideration of a realist review is to reflect on contextual factors of the social system(s) and how this affects the working of the intervention[32]. In developing the initial programme theory, we sought to view the informed consent process through the larger social system at micro (individual), meso (organisational, institutional) and macro (policy) levels, where interactions between individuals and levels can be understood better[34]. This was aimed at bringing about recommendations for change[35].

Selection and appraisal of documents (step 3)

Broad inclusion and exclusion criteria (see online supplementary file 3 Inclusion/Exclusion criteria) were applied in the selection and appraisal process, each document was assessed for relevance and rigour by EH and G H-B[35]. Decisions were based on whether a document was relevant to the over-arching research question and what its contribution would be to the review. Rigour was determined by assessing methodological rigour and whether a document included important information that may help with interpretation, as well as providing sufficient detail to determine trustworthiness[13]. Quality of data was noted in data extraction forms and considered during analysis and synthesis.

EH screened all documents identified through searches, by title and abstract. Second reviewer G H-B screened a random 10% of the documents[37]. To establish inter-reviewer agreement,

For peer review only - http://bmjopen.bmj.com/site/about/guidelines.xhtml

a kappa measure of k>0.8 was pre-determined, with a resultant kappa measure of 0.90 following second level screening by EH and G H-B .

Data analysis and synthesis (step 4)

A combination of annotations and notes, along with a data extraction form was used to classify information from documents about potential candidate theories , through noting CMOC concepts[36]. A data extraction form was adapted from Rycroft-Malone[38] to record characteristics of studies and documents and whether evidence was good enough and relevant to include in the synthesis. This included author, year, source, issues of sample size, data collection and data analysis, and interpretations made, characteristics of theoretical concepts and the impact these characteristics may have on the informed consent process (e.g. characteristics of appropriateness of informed consent and how these characteristics may impact on the decision to take part in research). The data extraction form was tested on the first three documents to confirm its usefulness.

Refinement and validation of programme theory (step 5)

In step 5, both data and annotations were combined. Data synthesis was undertaken by EH through mapping data to create a data matrix according to CMOCs which helped with interpretations and in developing theory[13]. Contexts were themed according to the level of the social system within which they related to[34]. Through noting common features, or relationships and identifying any underlying concepts, recurring themes were identified and patterns in the evidence were tested.

Recurring contexts and outcomes were explained through mechanisms. For example, within the included texts, the design of some research studies were reported as resulting in exclusion of people of diverse ethnic and cultural backgrounds, leading to mistrust, feelings of being exploited and a belief that academic communities are not committed to understanding the needs and experiences of people from diverse ethnic and cultural communities[2,31,40-41].

For peer review only - http://bmjopen.bmj.com/site/about/guidelines.xhtml

During data synthesis the aim was to provide an explanation of these contexts and outcomes through the identification of mechanisms. Further studies were sought to test the various elements of programme theory. Synthesis results were shared and discussed at stakeholders' meetings, to ensure validity and consistency of the interpretations made. The extraction of data, analysis and synthesis process was iterative with documents repeatedly read at various points throughout the process.

Development of recommendations (step 6)

Recommendations emerged through EH leading a remote discussion with stakeholders whereby the final programme theory was considered, leading to agreement upon a set of recommendations aimed at government agencies, Institutional Review Boards (IRBs), researchers, and health professionals. Input from stakeholders was intended to ensure the recommendations were realistic.

RESULTS

Document characteristics

In total, seventy-one studies/documents published between 2005 and 2020 were included from 19 countries with the majority from the USA (37%), UK (24%) and Africa (13%). The date range of the sources was from 2004 to 2020. The source type was mixed including : discussion papers and opinion pieces (17, 24%), qualitative research (27, 38%), literature reviews (4, 6%) , mixed methods research (2, 3%), systematic reviews (5, 8%), quantitative research (9, 13%), guidelines (2, 3%), case study (1, 1%), book chapter (1, 1%), audit (1, 1%), report (1, 1%) and a working paper (1, 1%).

Context Mechanism Outcome Configurations (CMOCs)

Themes emerged throughout examination of the data: inter-relationships; organisational and individual cultural competency and adaptive processes. Patterns in Mechanisms were identified as can be seen on the long list of CMOCs (see online supplementary file 4 CMOC

For peer review only - http://bmjopen.bmj.com/site/about/guidelines.xhtml

Long List). These are discussed below making reference to the associated CMOCs and selected quotations from documents which help to explain the theories, drawing out key aspects of the CMOCs.

Inter-relationships

Developing community relationships is important. Key family members, community, or faith leaders and local healthcare staff may influence a potential participant's decision to take part (CMOC 3, CMOC 5, CMOC 11)[3,46,48]. The approach to identifying individuals must be done sensitively with clear information provided about what participation involves (CMOC 1, CMOC 7, CMOC 9, CMOC 18, CMOC 26)[3,6,49-50]. This can lead to increased autonomy, trust and a positive view towards health research with the key individuals becoming a trusted point of information, thus helping to improve knowledge about health research, increasing the awareness and confidence of communities to participate in research[3].

> *…research assistants attempted to immerse themselves in the activities of the diabetes clinic…* [6] (P.7) (CMOC 1)

Researcher-matching may help (CMOC 1, CMOC 4) as it provides some choice for the participant as to whom they work with[7,51,53] and can provide effective and positive experiences for potential participants. This has been used in trials on diabetes, mental health, and cardiovascular disease with participants of South Asian heritage and Black British, African, and African-Caribbean heritage[44,54]. Examples include an interviewer speaking the same language as the participant conducts interviews; a health professional who speaks the same language as participant conducts intervention; an interpreter is present to support delivery of the intervention; trained multi-lingual facilitators; ethnically matched outreach team delivers the intervention; research assistants who speak the same language; and culturally-adapted interventions[2,9,31,43,45,47,54-59]. The cost implications of this approach were rarely reported, with only one study discussing the impact and challenges of

For peer review only - http://bmjopen.bmj.com/site/about/guidelines.xhtml

training facilitators and the complexities of translating material[9] . The perceptions of having interviews or focus groups conducted by someone of the same ethnicity is under-explored and viewed by some researchers as not as essential as language, gender, technical and interpersonal skills which may be more important[9]. There was some suggestion that researcher-matching should be used with caution as there is a risk that this may lead to researchers lacking cultural competence[60]. There is also a risk when using interpreters who have been trained to facilitate qualitative interviews that the richness of data could be compromised[61].

Family relationships may trigger a positive or negative experience of the decision-making process[2-3,71] (CMOC 5, CMOC 11). Identification of key family /community members involved in decision making process can determine whether someone takes part and conversely can improve the choice through improved understanding[2-3,60,71] (CMOC 5).

Organisational and individual cultural competency

There is considerable evidence to support the stance that organisations and the people within them should deliver services with cultural competence (CMOC 2, CMOC 9)[3,5]. Cultural competence is defined as the way in which behaviours, attitudes, and policies of a system, or amongst individuals, such as health care professionals, enables a service to operate effectively amongst diverse cultural situations[63]. Developing cultural competency and leadership helps to develop a capable, skilled research workforce (CMOC 1)[3,5,7,10] protecting the autonomy of potential participants, encouraging reflection on the individual's life situation, and developing awareness of different cultures[5].

Contextual factors such as the life situation of some populations e.g. migrant communities can influence the decision-making process in health research due to

> *...fear of identification and reporting to authorities…* [5](P.13) (CMOC 11)

For peer review only - http://bmjopen.bmj.com/site/about/guidelines.xhtml

Whilst cultural competence is evidenced to improve the cultural responsiveness and appropriateness in the way in which health research is delivered, as well as reducing health disparities, it requires further exploration that goes beyond the scope of this review[3,5,7,10,45]. There is some evidence to suggest that cultural competency conflates language, geographical origin, ethnicity, and race, potentially diluting the identity of individuals and communities[62]. The term has caused considerable debate since the introduction of the concept in the 1980's[63] with some asserting that it is not possible to be competent in other individuals' cultures[60,64]. However, cultural competence has been considered vital for engaging with diverse ethnic and cultural communities to ensure accurate information is shared appropriately to instil confidence in the safety and efficacy of COVID-19 vaccines[65]. Cultural competency when positioned within a human rights framework is viewed positively as a way of operationalising respect, providing a service that is culturally appropriate[62,66].

Adaptive processes

Providing small bitesize participant information about a health research study, in local languages, with local cultural context supports understanding[5,45,67]. In addition to this, co-production and more community based participatory approaches to health research are required, creating a more inclusive approach to study design and delivery, offering a way to empower individuals and communities who are under-served in health research[68]. It has been demonstrated that engagement with people of African-Caribbean heritage in the development of a culturally-adapted family intervention for people with schizophrenia can be achieved by taking a two-way approach, whereby researchers become integrated within communities and individuals from communities are able to access training and support around health research to increase capability as part of a community based participatory research (CBPR) approach, this has been demonstrated[69].

For peer review only - http://bmjopen.bmj.com/site/about/guidelines.xhtml

Taking these approaches improves accountability and makes information provided relevant.

> *…members of target populations should be involved in the overall design of research…*[53] (P.5) (CMOC 9, CMOC 14)

In addition to this, it may be helpful to offer a range of materials (e.g. written, oral, audio, and multi-media) to ensure concepts are understood[3,19] (CMOC 2, CMOC 12). Providing study information in various formats can promote feelings of independence, leading to consent being given freely, serving more respect for cultural differences[6,7,70] (CMOC 2, CMOC 14).

> *...this way you won't need to depend on your children anymore* [6](P6*)* (CMOC 3)

Some population groups lack an agreed written form of their main language, and alternative formats are useful in this respect (CMOC 2, CMOC 12)[6-7].

This creates trust and ensures dignity[3,5-6,10,44,45].

Institutional barriers

When difference is acknowledged but needs are misrepresented[3,5,45] (CMOC 6, CMOC 8, CMOC 13). This results in discriminatory outcomes (e.g. exclusion due to eligibility criteria); ignores needs of ethnically diverse populations; enforces inappropriate ethical, legislative, scientific guidelines and responses by health professionals[3,5,8] (CMOC 5, CMOC 13). Evidence from over a decade ago indicated that individuals from ethnic minorities are under-represented in making funding decisions about health research, the commission of it and on editorial boards of journals[9](CMOC 13). This is considered a limitation of this review as more time to explore this topic in the available literature would improve understanding as to whether and how much this situation has improved.

Study designs can restrict access to health research through creating eligibility criteria that excludes certain populations from taking part, usually due to a language barrier*[3]*

For peer review only

For peer review only - http://bmjopen.bmj.com/site/about/guidelines.xhtml

...many studies require participants to have the ability to understand and speak English which excludes older people from ethnic minority communities and/or migrants[3] (P.6) (CMOC 5, CMOC 9, CMOC 12, CMOC 13)[3,45,54].

This results in under-representation and may be perceived as institutional racism[2,8,54,58](CMOC 5, CMOC 9, CMOC 12, CMOC 13).

...dearth of research with ethnic minority communities in the UK is a reflection of the lack of influential policy... [3](P.9) (CMOC 6, CMOC 13)

Evidence from the United States suggests that institutional racism is not easily defined and encompasses not only individual prejudices, but prejudices deeply embedded within five areas: the law, practice, economy , culture and society , which results in unequal access to healthcare, including health research[72]. The role of institutional racism in health research may be fuelled by the additional costs for translation and extra resources affecting the chances of receiving funding for grant applications[54]. There is very limited evidence available on the role of institutional racism in health research. A systematic review undertaken in the US found that it is a concept that is rarely explicitly named in abstracts and titles and rarely engaged with, indicating that the lack of acknowledgement of its existence could be holding back discovery within this field[62-73].

A resultant programme theory was produced following steps 1-5 (figure 4).

Recommendations are displayed in Table 1. to illuminate the ways in which individuals, organisations, institutions and policymakers may improve the informed consent process, based upon discussions with stakeholders and resultant programme theory.

Table 1.

**Recommendation**	**Action required**

For peer review only - http://bmjopen.bmj.com/site/about/guidelines.xhtml

**1. Policymakers, organisations, institutions and individuals need to take responsibility and address institutional racism, acting on the issues that we are aware of.**	This requires:<br>- leadership and identification of best practice<br>- open discussion of culturally sensitive research ethics throughout the social system (e.g. values, beliefs and practices)<br>Organisations and institutions should raise awareness of institutional racism, what it is and how to address it.
**2. Build relationships with communities from diverse cultural and ethnic backgrounds to provide opportunities for Patient and Public Involvement (PPI) and empower individuals so that study designs become more relevant and culturally appropriate.**	Employ more staff from diverse ethnic and cultural backgrounds in academic institutions and health research organisations.<br>Seek community members from diverse ethnic and cultural backgrounds on ethics committees.<br>Provide cultural competency training for all involved in health research.<br>Identify local champions and/or leads to raise awareness of cultural competency in health research.<br>Context is important.

For peer review only - http://bmjopen.bmj.com/site/about/guidelines.xhtml

**3. Develop tailored informed consent in health research that offers choices (e.g. written, audio, multi-media).**	Ensure Patient and Public Involvement (PPI) by people of diverse ethnic and cultural backgrounds in study designs.
**4. Organisations and institutions need to develop cultural competence within them, increasing capability, knowledge and awareness of other cultures.**	This requires behaviour change and leadership.<br>All whom are involved in health research should undertake cultural competency training (Available at: Centre for Ethnic Health Research, Leicester, and West Midlands).

DISCUSSION

Statement of principal findings

This review has identified a broad range of issues and processes that are affecting the informed consent process for populations that are under-served by health research. The key themes around inter-relationships, organisational and individual cultural competency and adaptive processes may help to explain some of the contextual factors that influence the decision to take part in health research with these populations, more specifically those from diverse ethnic and cultural backgrounds. It has identified the elements that are necessary within a health research infrastructure to develop an inclusive health research system and has highlighted the limited evidence about how to adapt and tailor study designs and interventions successfully with people of diverse ethnic and cultural backgrounds. In particular, the lack of an adaptive informed consent process that considers inter-relationships

For peer review only - http://bmjopen.bmj.com/site/about/guidelines.xhtml

between researchers and participants and how life situations and family/community relationships may have a bearing on the decision to take part in health research has emerged as a key barrier to under-served populations participating in health research. Providing culturally competent care in health research respects diversity within populations and considers the effect of language, behaviours, attitudes, and cultural factors may have on inclusion[62].

Through deeper exploration using iterative literature searching, data emerged that indicated the use of complex study documentation e.g. patient information leaflets, communication barriers, lack of social presence in the community by researchers and a lack of diversity amongst researchers were identified as key realist mechanisms, which led to an explanation of the interaction with context to influence outcomes[2,8,42,47] .

At the individual level (micro) level, empowerment through access to resources such as culturally adapted information and the opportunity to be involved in the design of health research form a significant aspect of the programme theory. At the interpersonal and institutional levels (meso) enabling shared-decision making through approaches such as co-production; building community relationships and provision of researcher-matching and/or culturally competent researchers provides assurance and increases trust. Resources at the macro level are lacking, such as the lack of flexibility in approaches to the informed consent process, leading to negative outcomes with needs not being met and exclusion from health research, which may be perceived as institutional racism[41,54,58,73].

The data synthesis together with the stakeholder group meetings framed an overall programme theory in realist terms as:

> *If* organisations, institutions and individuals work together with diverse ethnic and cultural communities and become culturally competent, provide adaptive processes and empower community members to become involved , *then* this will

For peer review only - http://bmjopen.bmj.com/site/about/guidelines.xhtml

develop and result in trust, better engagement, shared-decision making and improved understanding.

Strengths and weaknesses in relation to other studies

Using a realist approach enabled a wider overview of more hidden factors that affect the decision-making process in health research, regarding the informed consent process with people of diverse ethnic and cultural backgrounds. The realist review has drawn from both peer-reviewed literature and grey literature as well as bringing in expertise from the stakeholder group. The lack of literature available on health research in general with people of diverse ethnic and cultural backgrounds is a weakness in itself as more specific evidence about what works, for whom and under what circumstances is urgently needed in order to help researchers design studies that are more inclusive and inform service development[3,5]. It is acknowledged that not all sources may have been discovered throughout this realist review process, in addition to not including the expertise of policymakers due to restrictions on time. It is hoped that the review will be useful to policymakers, organisations, institutions and health researchers in taking action to make changes to the service, for the benefit of those who need research the most[71].

Interpretation of the findings are somewhat dependent upon the researcher's skills and therefore , steps taken to conduct the review in accordance with RAMESES quality and publication standards were followed to ensure transparency[13,38].

The involvement of NIHR (Public) Research Champions, healthcare professionals, clinical academics and PPI Lead in refining the programme theory was felt to enhance the review's relevance. Having a second reviewer can increase the number of relevant studies included in a review and therefore this was considered a strength[5].

Suggestions for further research

For peer review only - http://bmjopen.bmj.com/site/about/guidelines.xhtml

From the review, cultural competence emerged as a recurring mechanism within the literature with further gaps in the evidence around : the way in which studies are designed and delivered and how people from under-served groups are involved in this aspect of the research process; institutional racism and its impact on health research; experiences of participation in health research with people of diverse ethnic and cultural backgrounds; the best adaptive informed consent processes, particularly for people of diverse ethnic and cultural backgrounds . In addition to this, further research is required to understand more the full range of requirements that may be needed to develop a more inclusive informed consent process, alongside in-depth enquiry around cultural competence and researcher-matching in relation to outcomes about research participation.

CONCLUSION

The resultant programme theory explains how, when, why and for whom the processes involved in gaining to consent to participate in health research does and does not work, providing suggestions for improvements to achieve greater inclusivity with people of diverse ethnic and cultural backgrounds, based on data synthesis and stakeholder expertise.

Some aspects of the programme theory are less well defined (e.g. institutional racism, cultural competency in health research, researcher-matching) and there is a need for more research on such topics to better understand how they affect outcomes. A critical finding is that a research infrastructure that supports and mandates inclusion is needed, as well as additional resources to support adaptive processes, support shared-decision making through involving more patients and the public in the design of health research studies. A greater focus on the evidence base developing in the field of global health community engagement, particularly around co-production , may provide valuable insights into how patients and the public from under-served communities can become involved in the design of health research studies[75].

Main document

For peer review only - http://bmjopen.bmj.com/site/about/guidelines.xhtml

Figure 1. Theory Map – Factors influencing decisions about whether to participate in health research with under-served populations – identified from scoping search

Figure 2. Theoretical concepts compounding positively, or negatively, the intervention

Figure 3. Flow diagram of searching

Figure 4. Final programme theory

online supplementary file 1 – Scoping searches

online supplementary file 2 – Searches

online supplementary file 3 – Inclusion/Exclusion criteria

online supplementary file 4 – CMOC Long List

LIST OF ABBREVIATIONS

NIHR National Institute for Health Research

PPI Patient and Public Involvement

CMOC Context Mechanism Outcome Configuration

UCL IRIS University College London Institutional Research Information System

IHE Institute for Health Equality

DECLARATIONS

Funding statement

This work was supported by a National Institute for Health Research (NIHR), HEE/NIHR ICA Programme Pre-doctoral Clinical Academic Fellowships *grant number* NIHR 300317. Funding for the open access charges for the publication of this protocol was provided by the NIHR Clinical Research Network West Midlands.

For peer review only - http://bmjopen.bmj.com/site/about/guidelines.xhtml

SS is part funded by the NIHR Applied Research Collaboration (ARC) West Midlands, the NIHR Health Protection Research Unit (HPRU) Gastrointestinal Infections, and the NIHR HPRU Genomics and Enabling data.

Disclaimer The views expressed in this publication are those of the author(s) and not necessarily those of the NIHR, HEE, NHS or the UK Department of Health and Social Care.

Competing Interests

None declared.

Ethics approval and consent to participate not applicable**.**

Consent for publication. Not applicable.

Availability of data and materials. Not applicable.

Competing interests. None declared.

Author contributions EH was the lead reviewer. G H-B completed second reviewer tasks. SS, JD, G H-B critically reviewed and edited the manuscript. EH was responsible for the initial design and drafting of the manuscript. All authors read and approved the final manuscript.

Acknowledgements We thank Samantha Johnson Information Specialist, for critically appraising the search strategy. We would also like to thank the following stakeholders involved in this review for their contribution to improving the quality and relevance of the final programme theory as well as the recommendations: Anne Devrell, Geoff Robson, Ifeanyi Sargeant, Mohini Samini, Dr. Mohammed Shaikh, Richard Stephens, Sandra Prew,

For peer review only - http://bmjopen.bmj.com/site/about/guidelines.xhtml

Dr. Farhana Lockhat, Dr. Emma Sutton , Zi Liew, Sarah Joshi, Claire Talbot, Jonathan Heffernan-Davies, Sylvia Turner, Mohamed Mooradun.

Patient consent for publication

Not required.

Data sharing statement

This review presents previously published and publicly available data. Please refer to the reference list and their authors for these research data.

References

1 Witham MD, Anderson E, Carroll C, et al. Developing a roadmap to improve trial delivery for under-served groups: results from a UK multi-stakeholder process. *Trials*. 2020. doi.org/10.1186/s13063-020-04613-7 [published First Online 1 Aug 2020]

2 Redwood S, Gill P. Under-representation of minority ethnic groups in research- call for action. *Br J Gen Pract*. 2013. doi:10.3399/bjgp13X668456 [published Jul 2013]

3 Jutlla, K, Raghavan R. Improving the recruitment of Black, Asian and Minority Ethnic (BAME) communities in health and social care research: a review of literature 2017.www.dora.dmu.ac.uk/handle/2086/13221 (accessed 28 Jun 2020)

4 Verastegui, E.Consenting of the vulnerable: The informed consent procedure in advanced cancer patients in Mexico. *BMC medical ethics*. 2006 doi:org/10.1186/1472-6939-7-13 [published Online First: 13 December 2006]

5 Vickers T, Craig G, Atkin K. Addressing Ethnicity in Social Care Research *Social Policy and Administration47(3):310 – 326* doi:org/10.1111/j.1467-9515.2012.00851.x [published Online First 24 May 2012]

6 Lloyd, C, Johnson, M, Mughal, S. Securing recruitment and obtaining informed consent in minority ethnic groups in the UK. BMC *Health Serv Res*.2008; 8: 68.doi:org/10.1186/1472-6963-8-68[published Online First 30 Mar 2008]

For peer review only - http://bmjopen.bmj.com/site/about/guidelines.xhtml

7 Masood Y, Bower P, Waheed MW, et al.Synthesis of researcher reported strategies to recruit adults of ethnic minorities to clinical trials in the United Kingdom: A systematic review. *Contemp Clin Trials*2019; 78:1-10. doi: 10.1016/j.cct.2019.01.004 [published First Online 8 Jan 2019]

8 Barata P, Guicciardi E, Ahmad F et al.Cross-cultural perspectives on research participation and informed consent. *Social Science & Medicine.*2006;62:479- www.academia.edu/9183270/Cross_cultural_perspectives_on_research_participation_and_informed_consent (accessed 01 Jun 2020)

9 Culley L, Hudson N, Rapport F. Using Focus Groups with Minority Ethnic Communities: Researching Infertility in British South Asian Communities. *Qualitative Health Research*. 2007;17(1):102-112. doi:10.1177/1049732306296506 [published First Online 1 Jan 2007]

10 Smart A, Harrison E. The under-representation of minority ethnic groups in UK medical research. *Ethn Health*. 2017 Feb;22(1):65-82. doi: 10.1080/13557858.2016.1182126 [published First Online 13 May 2016]

11 ICH .ICH E8 General considerations for clinical studies.2019.www.ema.europa.eu/en/ich-e8-general-considerations-clinical-studies (accessed Jun 2021)

12 Hoverd E, Staniszewska S, Dale J. The informed consent process in health research with under-served populations: a realist review protocol. *Syst Rev*. 2021;10: 103. doi.org/10.1186/s13643-021-01652-2 [published First Online 9 Apr 2021]

13 Wong G, Greenhalgh T, Westhorp G. et al. RAMESES publication standards: realist syntheses. *BMC Med.2013;* 11:21 . doi.org/10.1186/1741-7015-11-21[published First Online29 Jan 2013]

14 National Institute for Health Research. Research Champions 2020.www.nihr.ac.uk/patients-carers-and-the-public/i-want-to-help-with-research/research-champions.htm (accessed 20 Apr 2020)

15 Emmel N, Greenhalgh J, Manzano A, et al. *Doing Realist Research*. London: Sage 2018.

16 Verastegui E. Consenting of the vulnerable: the informed consent procedure in advanced cancer patients in Mexico *BMC Medical Ethics.* 2006;7:13 doi:10.1186/1472-6939-7-13 [published First Online 13 Dec 2006]

17 Calamaro CJ. Culture competence in research: research design and subject recruitment. *J Pediatr Health Care.* 2008 Sep-Oct;22(5):329-32. doi: 10.1016/j.pedhc.2008.05.007 [published 1 Sep 2008]

18 Francis-Graham S, Ekeke N, Nelson C, et al. Understanding how, why, for whom, and under what circumstances opt-out bloodborne virus testing programmes work to increase test engagement and uptake within prison: a rapid-realist review *BMC Health Services Research* .2019;19:152 doi.org/10.1186/s12913-019-3970-z [published First Online 8 Mar 2019]

19 Garrison, N Sathe, N, Antommaria, A *et al.* A systematic literature review of individuals' perspectives on broad consent and data sharing in the United

For peer review only - http://bmjopen.bmj.com/site/about/guidelines.xhtml

States. *Genet Med* 2016;18, 663–671. doi.org/10.1038/gim.2015.138 [published First Online 19 Nov 2015]

20 Hill, C Tawiah-Agyemang, S, Odei-Danso, B et al.Informed consent in Ghana: what do participants really understand? *Med Ethics* 2008;34:48–53. doi:10.1136/jme.2006.019059 [published First Online 21 Dec 2007]

21 Hughson J, Woodward-Kron R, Parker A, et al. A review of approaches to improve participation of culturally and linguistically diverse populations in clinical trials *Trials*. 2016 doi:10.1186/s13063-016-1384-3 [published First Online 26 May 2016]

22 Locher,J,Bronstein, J, Robinson et al. Ethical Issues Involving Research Conducted With Homebound Older Adults The Gerontologist *The Gerontological Society of America;*2006; 46: 2, 160–164. doi.org/10.1093/geront/46.2.160 [published First 1 Apr 2006]

23 Munthe C, Radovic S, Anckarsater H.Ethical Issues in forensic psychiatric research on mentally disordered offenders. *Bioethics*2010;24(1):34-35. doi:10.1111/j.1467-8519.2009.01773.x [published First Online 11 Dec 2009]

24 Mystakidou K ,Panagiotou I. Ethical and practical challenges in implementing informed consent in HIV/AIDS clinical trials in developing or resource-limited countries, SAHARA-J: *Journal of Social Aspects of HIV/AIDS*, 2009;6:2, 46-57. doi.org/10.1080/17290376.2009.9724930 [published First Online 28 Feb 2009]

25 Riesa N, Mansfield E, Sanson-Fisher R. Ethical and legal aspects of research involving older people with cognitive impairment: A survey of dementia researchers in *Australia International Journal of Law and Psychiatry. 2019;68.*doi.org/10.1016/j.ijlp.2019.101534 [published Feb 2020]

26 Shepherd, V, Wood, F, Griffith, R et al. Protection by exclusion? The (lack of) inclusion of adults who lack capacity to consent to research in clinical trials in the UK *.Trials.* 2019;20:474. doi.org/10.1186/s13063-019-3603-1[published First Online 5 Aug 2019]

27 Smith J. How ethical is ethical research? Recruiting marginalized, vulnerable groups into health services research. *Journal of Advanced Nursing* 2008;62(2), 248–257. doi: 10.1111/j.1365-2648.2007.04567.x [published First Online 3 Apr 2008]

28 Vallely L, Lees S, Shagi C et al. How informed is consent in vulnerable populations? Experience using a continuous consent process during the MDP301 vaginal microbicide trial in Mwanza, Tanzania *BMC Medical Ethics* 2010;11:10 .www.biomedcentral.com/1472-6939/11/10 (accessed Jul 2020)

29 Zion D, Briskman L, Loff B. Returning to History: The Ethisc of Researchign Asylum Seeker Health in Australia . *The American Journal of Bioethics*.2010; 10(2). doi.org/10.1080/15265160903469310 [published First Online 3 Feb 2010]

30 Lakes, K, Vaughan, E, Jones, M.et al. Diverse perceptions of the informed consent process: implications for the recruitment and participation of diverse communities in the National Children's Study. *American journal of community*

For peer review only - http://bmjopen.bmj.com/site/about/guidelines.xhtml

psychology 2012; 49(1-2), 215–232. doi.org/10.1007/s10464-011-9450-1[published First Online 14 Jun 2011]

31 Folayan M, Haire B, Harrison A, et al.Ethical Issues in Adolescents' Sexual and Reproductive Health Research in Nigeria. *Dev World Bioeth*. 2015;15(3):191-8. doi: 10.1111/dewb.12061[published First Online 9 Jun 2014]

32 Pawson, R, Greenhalgh, T, Harvey, G et al. 2004 *Realist Synthesis: An Introduction.* RMP Methods Paper 2/2004. www.researchgate.net/publication/228855827_Realist_Synthesis_An_Introduction/link/0fcfd507f0b7cbb2ce000000/download (accessed May 2020)

33 Kantilal K, Hardeman W, Whiteside H*,* et al. Realist review protocol for understanding the real-world barriers and enablers to practitioners implementing self-management support to people living with and beyond cancer *BMJ Open* 2020;10. doi: 10.1136/bmjopen-2020-037636 [published First Online 3 Sep 2020]

34 Malek- Ahmadi M. Macro-Level, Meso-Level, and Micro-Level Analysis 2020.www.coursehero.com/sg/introduction-to-sociology/macro-level-meso-level-and-micro-level-analysis/ (accessed Aug 2020)

35 Moore T, McArthur M, Noble-Carr D. More a marathon than a hurdle: towards children's informed consent in a study on safety. *Qualitative Research* 2018;18(1):88-107. doi:10.1177/1468794117700708 [published First Online 2 May 2017]

36 Pawson R, Greenhalgh T, Harvey G, et al. Realist Review - a new method of systematic review designed for complex policy interventions. *J Health Serv Res Policy.* 2005; 1:21-34.doi:10.1258/1355819054308530 [published 1 Jul 2005]

37 Stoll C, Izadi S, Fowler S et al. The value of a second reviewer for study selection in systematic reviews *Research synthesis methods*. 2019;10(4):539-545. doi.org/10.1002/jrsm.1369 [published First Online 4 Jul 2019]

38 Rycroft - Malone J, McCormack B, Hutchinson A. Realist synthesis: illustrating the method for implementation research. *Impl Science*. 2012;doi:10.1186/1748-5908-7-33 [published First Online 19 Apr 2012]

39 Scharff D, Mathews K, Jackson P, et al. More than Tuskegee: understanding mistrust about research participation. *J Health Care Poor Underserved*. 2010;21(3):879-897. doi:10.1353/hpu.0.0323 [published First Online Aug 2010]

40 Kao C, Aranda S, Krishnasamy M, et al. Identifying essential information to support patient decision-making regarding participation in cancer clinical trials: A Delphi study. *Eur J Cancer Care* (Engl). 2018 ;27(6).doi: 10.1111/ecc.12954 [published First Online 17 Sep 2018]

41 Grady, C, Hampson, L, Wallen, G et al Exploring the ethics of clinical research in an urban community. American journal of public health,

For peer review only - http://bmjopen.bmj.com/site/about/guidelines.xhtml

2006;96(11). doi.org/10.2105/AJPH.2005.071233 [published First Online 10 Oct 2011]

42 Kadam R. Informed consent process: A step further towards making it meaningful! *Perspectives in Clinical Research*. 2017; 8(3):107-12. doi10.4103/picr.PICR_147_16 [published Sept 2017]

43 Kleinman A, Benson P. Anthropology in the Clinic: The Problem of Cultural Competency and How to Fix It. *PLoS Med* 2006;3(10). doi.org/10.1371/journal.pmed.0030294 [published First Online 24 Oct 2006]

44 Jolly, K., Lip, G, Taylor, R,et al. Recruitment of ethnic minority patients to a cardiac rehabilitation trial: the Birmingham Rehabilitation Uptake Maximisation (BRUM) study *BMC medical research methodology* 2005;*5*, 18. doi.org/10.1186/1471-2288-5-18 [published First Online 17 May 2005]

45 Atkin, K. Institutional racism, policy and practice. In: Primary Healthcare and South Asian Populations–Meeting the Challenges. Oxford: Radcliffe Medical Press. 2004:1-12

46 McDougall G, Simpson G, Friend M. Strategies for research recruitment and retention of older adults of racial and ethnic minorities. *J Geront Nurs*. 2015. doi.org/10.3928/00989134-20150325-01 [published First Online 25 Mar 2015]

47 Kirby T. Evidence mounts on the disproportionate effect of COVID-19 on ethnic minorities. *The Lancet Respiratory Medicine*.2020.doi:10.1016/S2213-2600(20)30228-9 [published First Online 8 May 2020]

48 Heywood, R., Mackaskill, A,Williams K. Informed consent in hospital practice: health professionals' perspectives and legal reflections.*Medical Law Review,* 2010; 18 (2).www.pubmed.ncbi.nlm.nih.gov/20513795/ (accessed May 2020)

49 Flory J, Emanuel E. Interventions to improve research participants' understanding in informed consent for research: a systematic review *JAMA 2004;*292(13):1593-1601. doi:10.1001/jama.292.13.1593 [published 6 Oct 2004]

50 Mchale J. Innovation, informed consent, health research and the Supreme Court: Montgomery v Lanarkshire - a brave new world? *Health Econ Policy Law*. 2017;12(4):435-452. doi: 10.1017/S174413311700010X. [published First Online 27 Apr 2017]

51 Treweek S, Forouhi N, Narayan K,et al .COVID-19 and ethnicity:who will research results apply to? *The Lancet* 2020. 395(10242):1955.doi: 10.1016/S0140-6736(20)31380-5 [published First Online 12 Jun 2020]

52 Grewal, I. and Ritchie, J. Ethnic and language matching of the researcher and the research group during design, fieldwork, and analysis. In: Nazroo, J (ed) *Health and Social Research in Multiethnic Societies London:* Routledge :2006:1-17 www.taylorfrancis.com/chapters/edit/10.4324/9780203969939-12/ethnic-language-matching-researcher-research-group-design-%EF%AC%81eldwork-analysis-ini-grewal-jane-ritchie (accessed May 2020)

53 Vickers, T ,Craig, G,Atkin, K. Research with black and minority ethnic people using social care services. SSCR methods review, 2012;11. NIHR School for Social Care Research, London, UK.www.eprints.lse.ac.uk/43161/1/SSCR_Methods_Review_11_(lsero).pdf (accessed Apr 2020)

54 Hussain-Gambles M, Atkin K, Leese B. South Asian participation in clinical trials: the views of lay people and health professionals. Health Policy. 2006 ;77(2):149-65. doi: 10.1016/j.healthpol.2005.07.022.[published First Online 10 Oct 2005]

55 McDougall G, Simpson G, Friend M. Strategies for research recruitment and retention of older adults of racial and ethnic minorities. *J Geront Nurs.* 2015.doi:10.3928/00989134-20150325-01 [published First Online 30 Mar 2015]

56 Ries N, Thompson K, Lowe M. Including People with Dementia in Research: An Analysis of Australian Ethical and Legal Rules and Recommendations for Reform. *J Bioeth Inq.* 2017;14(3):359-374. www. https://psycnet.apa.org/record/2017-38470-007 (accessed Apr 2020)

57 Dawson, S, Campbell S, Giles S et al. "Black and minority ethnic group involvement in health and social care research: A systematic review." Health expectations: an international journal of public participation in health care and health policy 2018;21(1): 3-22. doi:10.1111/hex.12597 [published First Online 15 Aug 2018]

58 Wiles, R, Charles V, Crow G et al. 'Researching researchers: lessons for research ethics. *Qualitative Research*, 2006;6(3): 283–299.www. https://eprints.soton.ac.uk/40739/ (accessed Aug 2021)

59 Hamel L, Penner L, Albrecht T, et al. Barriers to Clinical Trial Enrollment in Racial and Ethnic Minority Patients With Cancer. *Cancer Control*. 2016;23(4):327-337. doi:10.1177/107327481602300404 [published First Online 1 Oct 2016]

60 Chavez, V. Cultural humility: Reflections and relevance for CBPR. In Wallerstein, N., Duran, B., Oetzel, J., Minkler, M. (Eds.), Community-based participatory research for health: Advancing social and health equity 2018; 357-362. [doi.org/10.1177/1524839919884912 [published First Online 12 Nov 2019]

61 Erves J, Mayo-Gamble T, Malin-Fair A, et al. Needs, Priorities, and Recommendations for Engaging Underrepresented Populations in Clinical Research: A Community Perspective. *J Community Health*. 2017 ;42(3):472-480. doi: 10.1007/s10900-016-0279-2. [published First Online 3 Nov 2016]

62 Jongen, C, McCalman, J, Bainbridge, R. Health workforce cultural competency interventions: a systematic scoping review. *BMC Health Serv Res* 2018;18(232) . doi.org/10.1186/s12913-018-3001-5 [published First Online 2 Apr 2018]

63 Cross T, Bazron B, Dennis K, et al. Towards a culturally competent system of care.1989;Georgetown University Child Development Center, CASSP

For peer review only - http://bmjopen.bmj.com/site/about/guidelines.xhtml

Technical Assistance Center, Washington, DC. www. https://files.eric.ed.gov/fulltext/ED330171.pdf (accessed May 2020)

64 Minkler, M, Pies, C, Hyde, C. Ethical issues in community organizing and capacity building. In Minkler, M. (Ed.), Community organizing and community building for health and welfare .New Brunswick, NJ: Rutgers University Press. 2012:110-129

65 Hanif W, Ali S, Patel K et al.Cultural competence in Covid-19 vacine rollout *BMJ* 2020;371:4845 doi:10.1136/bmj.m4845.*doi: 10.1136/bmj.m4845* [published First Online 18 Dec 2020]

66 National Institute of Healthcare (NIH). 2019;Cultural respect.www.nih.gov/institutes-nih/nih-office-director/office-communications-public-liason/clear-communication/cultural-respect (accessed Apr 2020)

67 Ahikari B, Vincent R, Wong, G. A realist review of community engagement with health research. *Wellcome Open Research* 2019; 4(87). doi: 10.12688/wellcomeopenres.15298.2 [published First Online 2 Aug 2019]

68 Fiske A, Prainsack B, Buyx A. *J Med Ethics* 2019;45:617–622. doi:10.1136/medethics-2018-105253 [published First Online 12 Jul 2019]

69 Edge, D, Grey, P. An Assets-Based Approach to Co-Producing a Culturally Adapted Family Intervention (CaFI) with African Caribbeans Diagnosed with Schizophrenia and Their Families. *Ethnicity & disease*, *28*(Suppl 2), 485-492.www.ethndis.org/edonline/index.php/ethndis/article/view/951 (accessed Sept 2020)

70 Nnaji C, Boone M, Pugnaire MP, et al.An Innovative Simulation-based Community-engaged Intervention for Training Research Assistants in Culturally Appropriate Informed Consent. Prog Community Health Partnersh. 2018;12(3):247-262. doi: 10.1353/cpr.2018.0049 [published First Online 28 Nov 2017]

71 Cheah, P, Parker, M. Research consent from young people in resource-poor settings. *Archives of disease in childhood.*2014. doi:10.1136/archdischild-2014-307121.[published May 2015]

72 Bailey, Z, Feldman, J, Bassett, M. How Structural Racism Works — Racist Policies as a Root Cause of U.S. Racial Health Inequities. *New England Journal of Medicine* 2021;384, 768–773.doi:10.1056/nejmms2025396 [published First Online 25 Feb 2021]

73 Hardeman R, Murphy K, Karbeah J,et al. Naming Institutionalized Racism in the Public Health Literature: A Systematic Literature Review. Public Health Report . 2018;133(3): 240-249. doi: 10.1177/0033354918760574[published First Online 3 Apr 2018]

74 Booth M. Informed consent in emergency research: a contradiction in terms. *Sci Eng Ethics.* 2007;13(3):351-9. doi.org/10.1007/s11948-007-9028-3 [published First Online 3 Aug 2007]

75 Redman S, Greenhalgh T, Adedokun L et al.(2021) Co-production of knowledge: the future A new collection highlights the role of co-production in

For peer review only - http://bmjopen.bmj.com/site/about/guidelines.xhtml

strengthening health systems 2021;372.doi.org/10.1136/bmj.n434 [published First Online 16 Feb 2021]

For peer review only

For peer review only - http://bmjopen.bmj.com/site/about/guidelines.xhtml

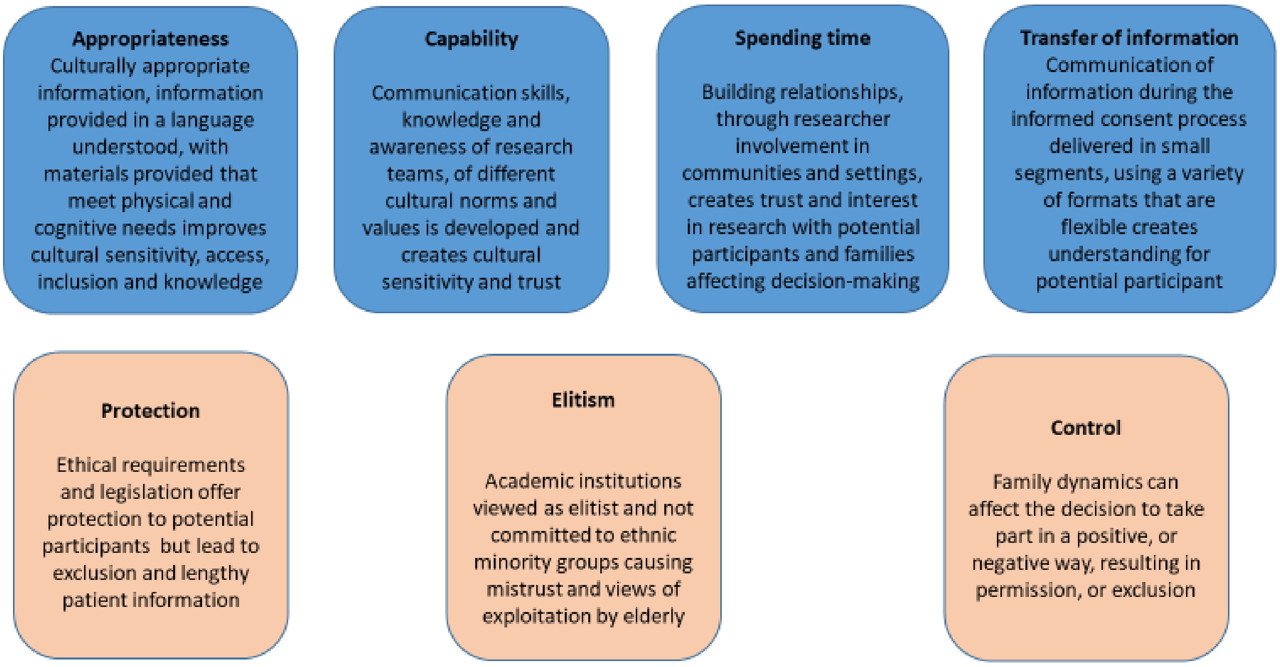

Figure 1. Theory Map –Factors influencing decisions about whether to participate in health research with under-served populations – identified from scoping search

Figure 2.

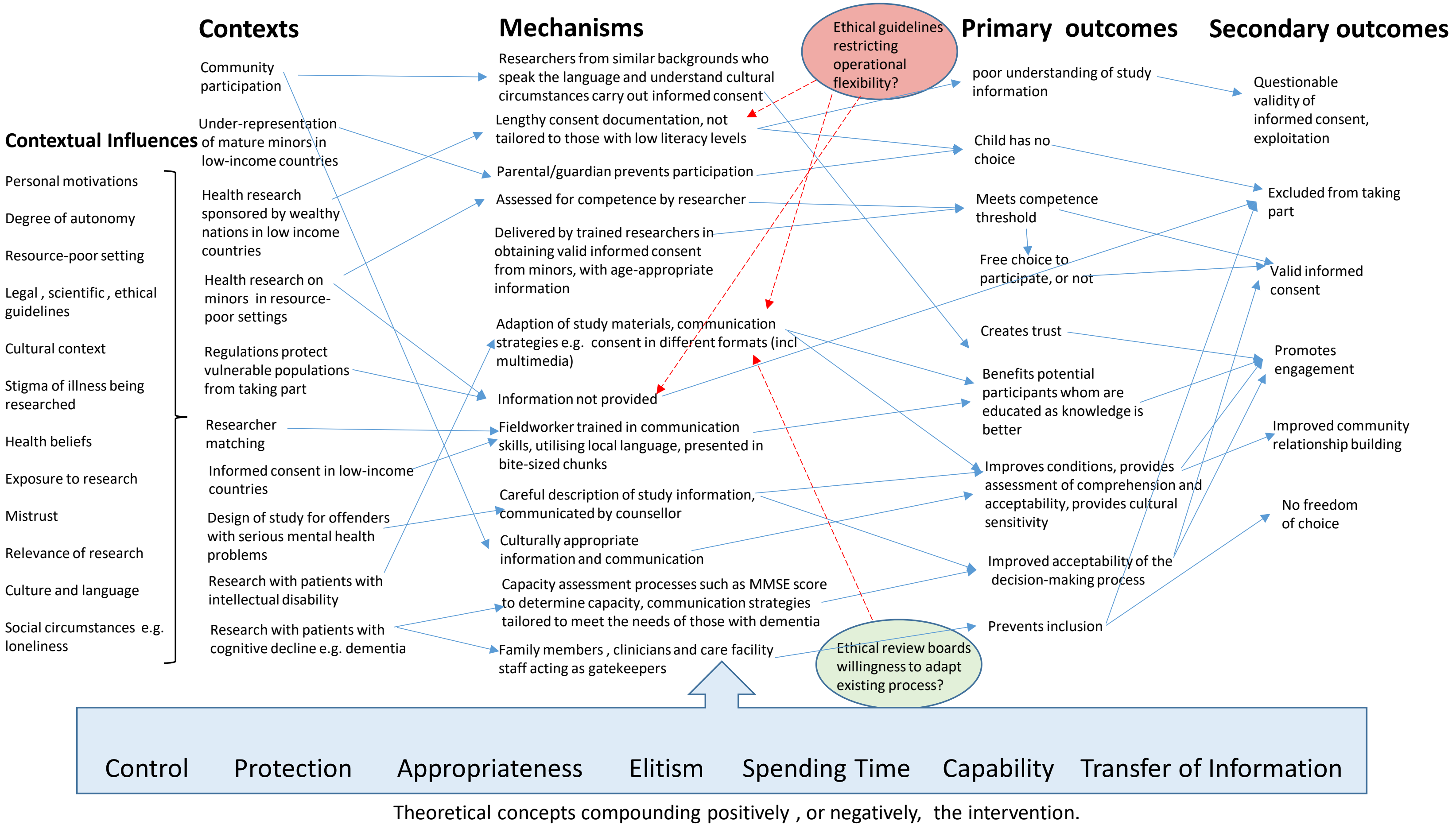

For peer review only - http://bmjopen.bmj.com/site/about/guidelines.xhtml

Figure 3.

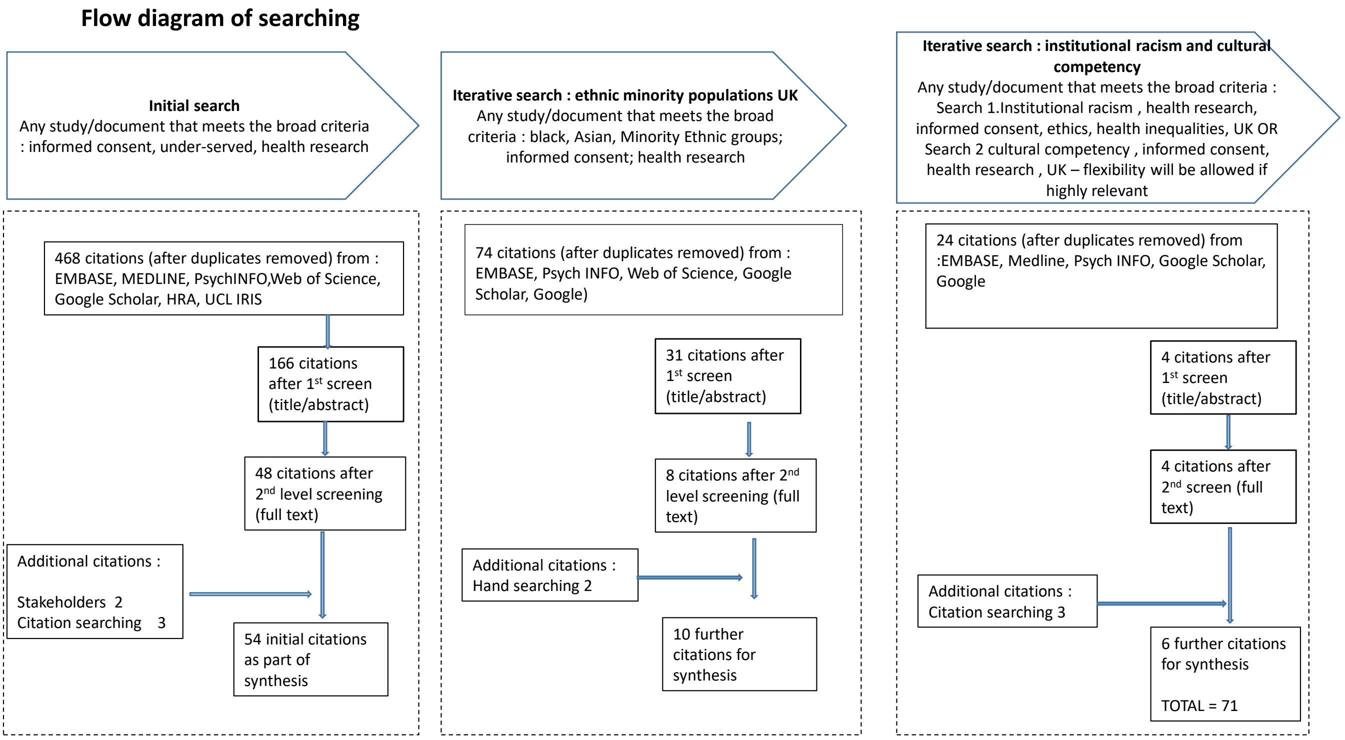

review only

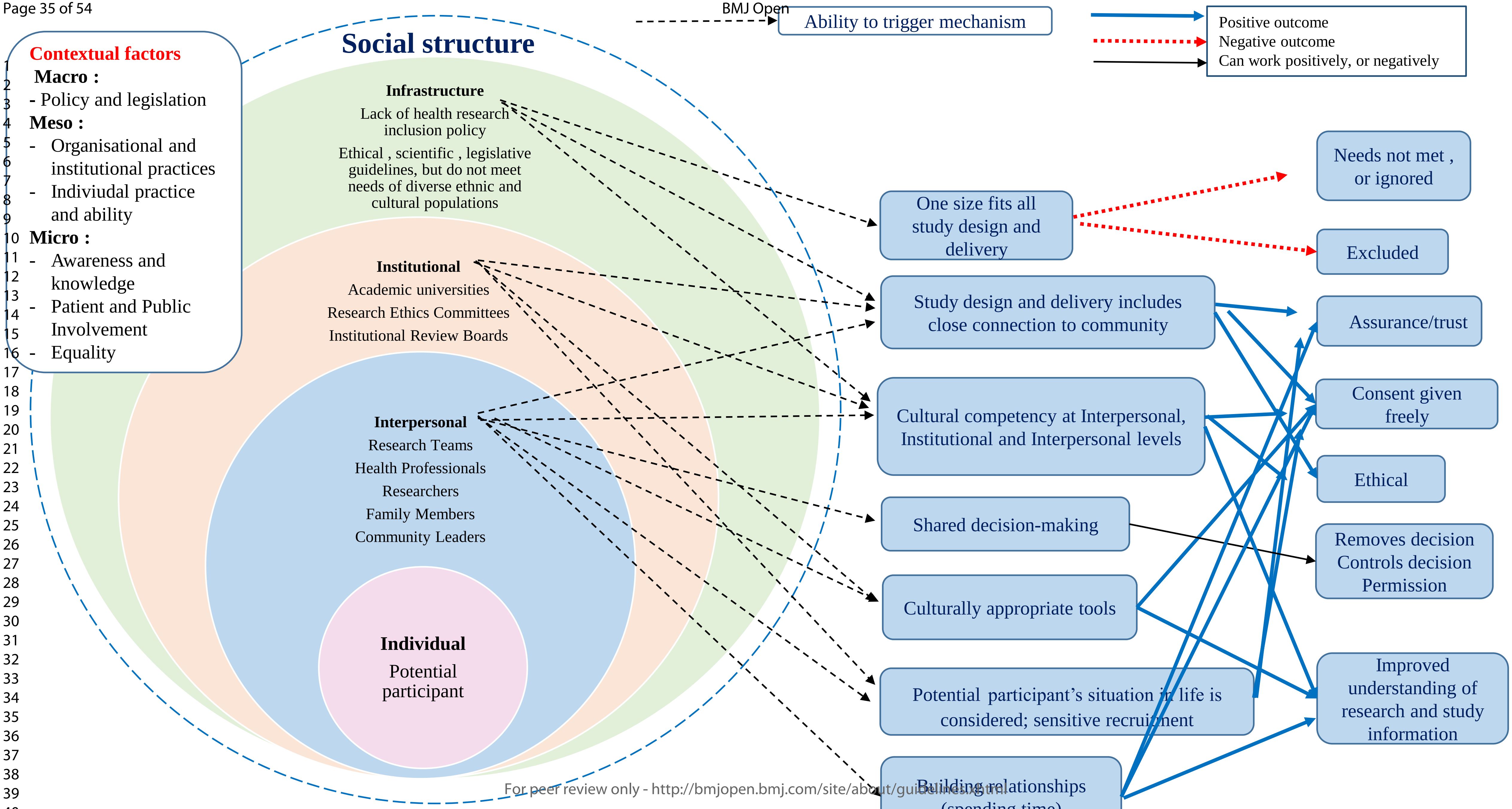

Final programme theory

Scoping Searches

Medline Scoping Search
Key terms informed consent, under-served , under-represented, health research, biomedical research 2005-current, English only

Database: Ovid MEDLINE(R) <1946 to May Week 2 2020>
Search Strategy:
----
1 informed consent.mp. or Informed Consent/ (57316)
2 exp Telemedicine/ or under-served.mp. or exp Vulnerable Populations/ (38247)
3 under-representation.mp. (756)
4 1 and 2 and 3 (0)
5 1 and 2 (820)
6 health research.mp. or exp Biomedical Research/ (277290)
7 5 and 6 (373)
8 limit 7 to (english language and yr="2005 -Current") (181)

****

Web of Science search May 21 2020

Key words : informed consent and under represented

Limits : English, 2005-current

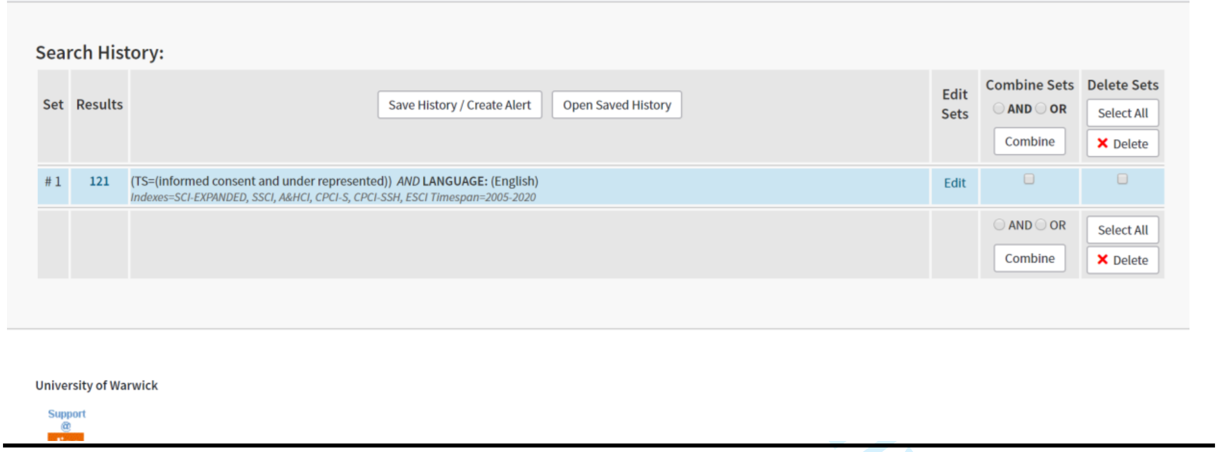

Database : UCL IRIS
Keywords : informed consent, under-served, health research, under represented

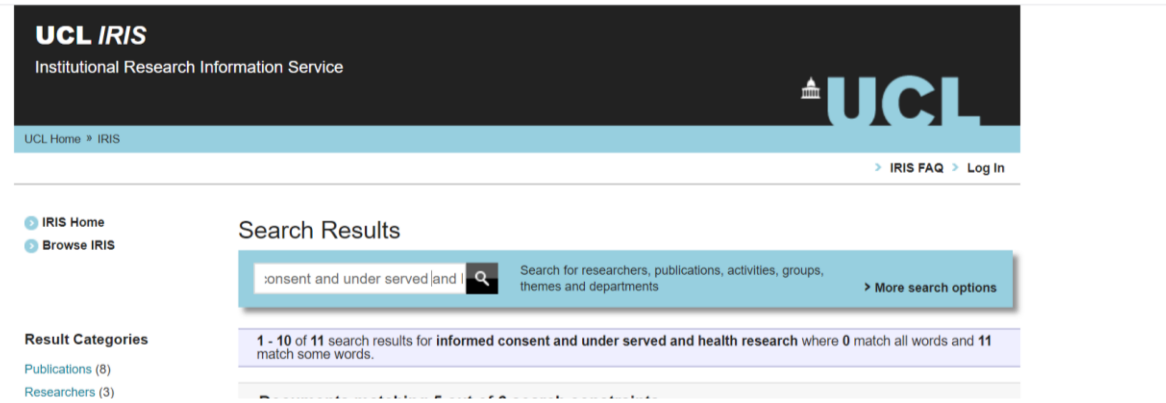

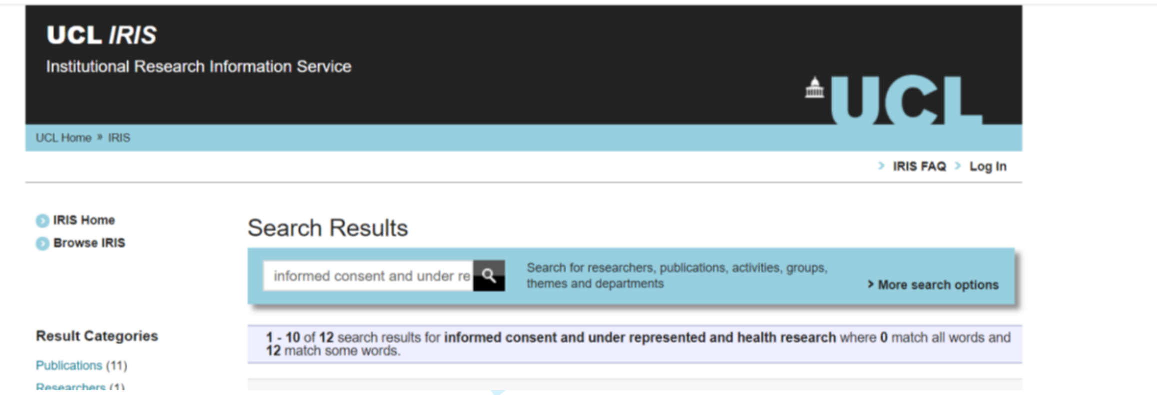

HRA (Health Research Authority)
Keywords: informed consent, underrepresented, health research

review only

EMBASE SEARCH

Database: EMBASE <1947 to 2020 Week 20>

Search Strategy:

----

1 informed consent.mp. or exp informed consent/ (128719)

2 health research.mp. or exp medical research/ (433296)

3 1 and 2 (9419)

4 under served.mp. (587)

5 vulnerable populations.mp. or vulnerable population/ (20469)

6 under-served.mp. (587)

7 4 and 5 and 6 (27)

8 4 or 5 or 6 (21029)

9 3 and 8 (218)

10 limit 9 to english language (211)

****

Medline Search 1

Database: Ovid MEDLINE(R) <1946 to May Week 1 2020>

Search Strategy:

----

1 informed consent.mp. or exp Informed Consent/ (62033)

2 under served.mp. (312)

3 Health Services Research/ or health research.mp. or Biomedical Research/ (118031)

4 1 and 3 (2832)

5 under-served.mp. or exp Vulnerable Populations/ (10894)

6 4 and 5 (107)

****

Web of Science

TOPIC: (informed consent and under represented and health research)
Refined by: [excluding] PUBLICATION YEARS=(2004 OR 2003 OR 1993)

For peer review only

Timespan=All years. Indexes=SCI-EXPANDED, SSCI, A&HCI, CPCI-S, CPCI-SSH, ESCI.

"Title","Authors","Corporate Authors","Editors","Book Editors","Source Title","Publication Date","Publication Year","Volume","Issue","Part Number","Supplement","Special Issue","Beginning Page","Ending Page","Article Number","DOI","Conference Title","Conference Date","Total Citations","Average per Year","1900","1901","1902","1903","1904","1905","1906","1907","1908","1909","1910","1911","1912","1913","1914","1915","1916","1917","1918","1919","1920","1921","1922","1923","1924","1925","1926","1927","1928","1929","1930","1931","1932","1933","1934","1935","1936","1937","1938","1939","1940","1941","1942","1943","1944","1945","1946","1947","1948","1949","1950","1951","1952","1953","1954","1955","1956","1957","1958","1959","1960","1961","1962","1963","1964","1965","1966","1967","1968","1969","1970","1971","1972","1973","1974","1975","1976","1977","1978","1979","1980","1981","1982","1983","1984","1985","1986","1987","1988","1989","1990","1991","1992","1993","1994","1995","1996","1997","1998","1999","2000","2001","2002","2003","2004","2005","2006","2007","2008","2009","2010","2011","2012","2013","2014","2015","2016","2017","2018","2019","2020"

Database: APA PsycInfo <1806 to May Week 4 2020>

Search Strategy:

----

1 (under-served or underserved).mp. [mp=title, abstract, heading word, table of contents, key concepts, original title, tests & measures, mesh] (5394)

2 health research.mp. (5729)

3 informed consent.mp. or exp Informed Consent/ (11199)

4 biomedical research.mp. (5165)

5 (under represented or under-represented).mp. [mp=title, abstract, heading word, table of contents, key concepts, original title, tests & measures, mesh] (1120)

6 underserved.mp. (5126)

7 under served.mp. (294)

8 vulnerable population.mp. (2420)

9 6 or 7 (5394)

10 informed consent.mp. or exp Informed Consent/ (11199)

11 decision making.mp. or exp Decision Making/ (167677)

12 research.mp. or exp Experimentation/ (1223074)

13 10 or 11 or 12 (1339577)

14 9 and 13 (2287)

15 1 or 5 or 6 or 7 (6498)

16 2 and 3 (168)

<u>Iteration 1- search</u>

Database: APA PsycInfo <1806 to May Week 3 2020>

Search Strategy:

----

1 INFORMED CONSENT.mp. or exp Informed Consent/ (11049)

2 HEALTH RESEARCH.mp. (5578)

3 exp Blacks/ or exp At Risk Populations/ or UNDER-SERVED.mp. or exp Disadvantaged/ (94625)

4 1 and 2 and 3 (7)

Database: Embase Classic+Embase <1947 to 2020 Week 20>

Search Strategy:

----

1 informed consent.mp. or informed consent/ (131165)

2 health research.mp. or medical research/ (234000)

3 Black.mp. or Black person/ (178998)

4 At risk populations.mp. (3605)

5 Under-served.mp. (601)

6 disadvantaged.mp. (15612)

7 1 and 2 and 3 (20)

Web of Science <u>17</u>	TOPIC: (informed consent and minority ethnic groups and health research)<br>**Refined by:** [excluding] **PUBLICATION YEARS:** (2004 OR 2002)<br>*Indexes=SCI-EXPANDED, SSCI, A&HCI, CPCI-S, CPCI-SSH, ESCI Timespan=All years*

Database: Ovid MEDLINE(R) <1946 to June Week 1 2020>

Search Strategy:

For peer review only

----

1 informed consent.mp. or Informed Consent/ (57978)

2 health research.mp. (12581)

3 Black.mp. (102740)

4 at risk populations.mp. (2115)

5 under-served.mp. or Vulnerable Populations/ (10932)

6 disadvantaged.mp. or Vulnerable Populations/ (20499)

7 1 and 2 and 3 (1)

8 2 and 5 (101)

9 1 and 8 (22)

****

For peer review only

Iteration 2 – Searches

(Search 1)

Database: Embase Classic+Embase <1947 to 2020 Week 35>

Search Strategy:

----

1 institutional racism.mp. (185)

2 health research.mp. or medical research/ (234000)

3 informed consent.mp. or informed consent/ (131165)

4 1 and 2 (3)

5 limit 4 to yr="2005 -Current" (1)

****

Database: APA PsycInfo <1806 to July Week 1 2020>

Search Strategy:

----

1 INSTITUTIONAL RACISM.mp. (453)

2 health research.mp. (5729)

3 informed consent.mp. or exp Informed Consent/ (11199)

4 1 and 2 (2)

5 limit 2 to yr =”2005-Current” (0)

Database : Web of Science July Week 1

Results: 2

(from Web of Science Core Collection)

You searched for: **TOPIC:** (institutional racism and health research and informed consent)

Timespan: All years. **Indexes:** SCI-EXPANDED, SSCI, A&HCI, CPCI-S, CPCI-SSH, ESCI.

Database: Ovid MEDLINE(R) <1946 to July Week 1 2020>

Search Strategy:

----

1 institutional racism.mp. (115)

2 Health Services Research/ or health research.mp. or Biomedical Research/ (118339)

3 1 and 2 (9)

4 limit 3 to yr="2005 -Current" (5)

(Search 2)

Database: Embase Classic+Embase <1947 to 2020 Week 25>

Search Strategy:

----

1 cultural competency.mp. or cultural competence/ (7186)

2 health research.mp. or medical research/ (234000)

3 1 and 2 (213)

4 informed consent.mp. or informed consent/ (131165)

5 3 and 4 (9)

Database: Ovid MEDLINE(R) <1946 to July Week 1 2020>

Search Strategy:

----

1 cultural competency.mp. or Cultural Competency/ (6133)

2 Health Services Research/ or health research.mp. or Biomedical Research/ (118339)

3 1 and 2 (196)

For peer review only

4 informed consent.mp. or Informed Consent/ (58028)

5 3 and 4 (8)

Database: APA PsycInfo <1806 to July Week 1 2020>

Search Strategy:

----

1 cultural competency.mp. (2952)

2 health research.mp. (5729)

3 informed consent.mp. or exp Informed Consent/ (11199)

4 1 and 2 and 3 (1)

Results: 9

(from Web of Science Core Collection)

You searched for: **TOPIC:** (cultural competency and health research and informed consent)

Timespan: All years. **Indexes:** SCI-EXPANDED, SSCI, A&HCI, CPCI-S, CPCI-SSH, ESCI.

For peer review only

Inclusion/Exclusion criteria

Inclusion criteria

- Quantitative and qualitative studies, grey literature, websites, stakeholder recommendations (e.g. reports, conference papers).
- The search will be limited to a 15-year publication period (2005 – 2020) to provide a large enough overview of relevant literature.
- All studies that report on experiences and decision-making involved in the informed consent process in under-served populations will be included.
- Any source of data that is deemed relevant by the stakeholder group.
- Literature that explores the concept and practice of informed consent.

Exclusion criteria

- Studies, or sources of data that are not relevant to the informed consent process in health research in relation to under-represented populations.

only

CMOC Long list

The CMOC's shaded blue are specific to people of diverse ethnic and cultural backgrounds in the UK. All other CMOC's include those from the initial programme theory.

CMOC	Context (How designed and delivered)	Mechanism (tailored, how info provided, delivered)	Outcome (decision-making)	Does it work?
**CMOC1**	**C2 Meso level Organisations and people within them should deliver services with cultural competence**	**M1 Skilled , culturally competent researchers Promotes positive and effective relationships with diverse communities . Researchers need to develop and employ sensitive skills to work with people from different cultural backgrounds**	**Meets the social, cultural, religious and linguistic needs of ethnic minority populations**	**yes**
**CMOC2**	**C1 Macro level C2 Meso level informed consent can be delivered in other formats apart from written**	**M4 Flexible processes, tailored information oral, audio-recorded study information and verbal consent**	**promotes feelings of independence;**	**yes**
**CMOC3**	**C2 Meso level communities advertising research**	**M5 Community involvement shopkeepers and mosque leaders as advocates for research creates enthusiasm**	**influences decision to take part**	**yes**

For peer review only

CMOC4	C2 Meso level appropriate matching of researcher to group being researched	M4 Flexible processes, tailored information consideration of the gender of the researcher due to religious reasons can affect engagement with discussion; matching ethnic background of researcher to ethnic background of participant	participant does not feel comfortable to discuss problems in some cases depending on gender of researcher, due to religious reasons; may prevent some participants from airing counter-cultural views	no
CMOC 5	M1 Macro level<br>M2 Meso level	M4 Flexible processes, tailored information involving family, community members, or local health professionals to support shared decision making	shared decision making	yes
CMOC4	C2 Meso level ethnic matching	M4 Flexible processes, tailored information participant has some choice in which researcher they work with	effective and positive experience for potential participant, having a bearing on decision-making about taking part	yes
CMOC5	C1 Macro level<br>C2 Meso level information provided only in English	M2 Removing/restricting choice considered institutionalised racism	excludes older ethnic minority populations and migrants	no
CMOC6	C1 Macro level<br>C2 Meso level study design restricts access	M3 Inflexible process<br>M2 Removing/Restricting choice	under-representation	no

For peer review only

		eligibility criteria excludes certain populations		
CMOC7	C2 Meso level commitment to confidentiality	M1 Skilled, culturally competent researchers researchers qualify confidentiality before obtaining consent, confirming comprehension	reduces fear of being reported to authorities (if migrant pop) and prevents decision of obedience to "community leaders"	yes
CMOC8	C1 Macro level C3 Micro level illiteracy, knowledge of health research, legal aspects	M3 Inflexible process signed informed consent , lacking in assurance of confidentiality	feel undue pressure to participate , or refuse due to fear of a negative impact on immigration process	no
CMOC9	M1 Macro level M2 Meso level study design	M1 Skilled, culturally competent researchers awareness of local protocol, legislation and culture; consideration of potential participants current situation in life; potential participant's awareness of the study; sensitive recruitment	culturally appropriate instruments , successful informed consent	yes

For peer review only

**CMOC11**	**M3 Micro level**<br>**life situation of some populations e.g. migrant communities**	**M2 Removing/restricting choice**<br>**M3 Inflexible process**<br>**individual decision-making not possible due to family relationships**	**identification of key family/community member involved in decision making with improved**	**yes**
**CMOC12**	**M1 Macro level**<br>**M2 Meso level**<br>**design and delivery of study**	**M4 Flexible processes, tailored information**<br>**close connection to community - conversations are basis for informed consent , summarising information, explaining written information (this can be supported by traditional methods, witnessed consent, audio-recording, video-taping or illustrations)**	**consent given freely, respect for cultural differences, improves trust , is ethical and ensures dignity**	**yes**
**CMOC13**	**M1 Macro Level**<br>**M2 Meso level**<br>**wider health care system**	**M3 Inflexible processes**<br>**M2 Removing / restricting choice**<br>**policies of institutions**<br>**1. presumption that one size fits all**<br>**2. difference is acknowledged but needs are misrepresented**	**discriminatory outcomes (e.g. exclusion due to eligibility criteria); ignores needs of ethnically diverse populations; enforces inappropriate policy and responses by health professionals**	**no**

For peer review only

CMOC14	M1 Macro level<br>M2 Meso level<br>primary health care	M2 Removing/restricting choice<br>gaining access<br>institutional racism used as a framework in healthcare	helps to understand discrimination and disadvantages minority ethnic groups experience	yes
CMOC15	M1 Macro level<br>low-income countries	M2 Removing/restricting choice<br>parental/guardian control	results in exclusion from taking part	no
CMOC 16	M1 Macro level<br>M2 Meso level<br>mature minors (often under-represented)	M4 Flexible processes, tailored information assessed for competence	meet competence threshold, providing valid informed consent	yes
CMOC 17	M1 Macro level<br>health research sponsored by wealthy nations in low income countries	M3 Inflexible process<br>Lengthy consent documentation, not tailored to those with low literacy levels	Inducement , poor understanding of study information, questionable validity of informed consent , exploitation	no
CMOC 18	M1 Macro level<br>health research in resource-poor settings	M1 Skilled, culturally competent researchers<br>delivered by trained researchers in obtaining valid consent from minors, with age-appropriate study information	free choice to participate	unclear
CMOC19	M1 Macro level<br>regulations protect vulnerable populations from taking part	M2 Removing /restricting choice<br>information not provided	removes freedom of choice	no

For peer review only

CMOC 20	M2 Meso level researcher matching	M1 Skilled, culturally competent researchers researchers from similar backgrounds who speak the language , understand cultural circumstances	creates trust	unclea r
CMOC2 1	M1 Macro level informed consent in low-income countries	M1 Skilled, culturally competent researchers fieldworker trained in communication skills and utilising local language to share information, in bite-size chunks	benefits potential participants whom are educated as knowledge is better	unclea r
CMOC2 2	M2 Meso level research protocol design	M3 Inflexible process M2 Removing/restricti ng choice complex forms and communication barriers, lack of social presence in the community, lack of diversity amongst researchers	results in exclusion, experience of mistrust, feel exploited and that academic institutions are not committed to ethnic minority communities, suspicion of formal transaction	no
CMOC2 3	M2 Meso level community participation and ethical review boards willingness to adapt existing process	M4 Flexible processes, tailored information adaptation of resources, consent in different formats (incl multimedia),	creates trust, better conditions , assessment of comprehension & acceptability, cultural sensitivity promotes access, community	unclea r

For peer review only

			relationship building	
CMOC2 4	M2 Meso level<br>design of study for offenders with serious mental health problems	M4 Flexible processes, tailored information<br>careful description of study, how it is communicated and by whom, in conjunction with dialogue-type counselling techniques , for patients whom are deemed to be able to make a competent decision	reduces coercion and confusion of role as a patient as opposed to a potential participant	unclea r
CMOC2 5	M1 Macro level<br>ethical guidelines for informed consent	M2 Removing choice/restricting choice<br>restrict operational flexibility	lack of cultural sensitivity and appreciation of family and cultural dynamics that affect decision-making	no
CMOC2 6	M1 Macro level<br>many African countries	M1 Skilled, culturally competent researchers<br>culturally appropriate delivery, taking into consideration social and cultural conventions, familiarity with local context of research	improved acceptability of the decision-making process	yes
CMOC2 7	M2 Meso level<br>M1 Micro level	M2 Removing/restricting choice	prevents inclusion, and	no

For peer review only

	research with patients with cognitive decline	family members, clinicians and care facility staff acting as gatekeepers	freedom of choice	
CMOC28	M2 Meso Level<br>M1 Micro level<br>research with patients with cognitive decline e.g. dementia	M4 Flexible processes, tailored information capacity assessment processes such as a MMSE score, as a way of determining capacity to consent and communication strategies tailored to meet needs of those with dementia	enables person to make own decision , whilst is a continuous check of willingness	yes
CMOC29	M2 Meso level<br>M1 Micro level<br>research with people with intellectual disability	M4 Flexible processes, tailored information adapting study materials and communication strategies during the informed consent process	builds rapport and engagement with participant and enables them to participate in the consent process to the best of their ability	yes

For peer review only

PRISMA 2020 Checklist

Section and Topic	Item #	Checklist item	Location where item is reported
**TITLE**			
Title	1	Identify the report as a systematic review.	N/A (realist review) RAMESES guidelines : http://www.ramesesproject.org/
**ABSTRACT**			
Abstract	2	See the PRISMA 2020 for Abstracts checklist.	N/A
**INTRODUCTION**			
Rationale	3	Describe the rationale for the review in the context of existing knowledge.	Page 5 (lines 109-110)
Objectives	4	Provide an explicit statement of the objective(s) or question(s) the review addresses.	Page 5 (lines 114-115)<br>Page 6 (lines 120-125)
**METHODS**			
Eligibility criteria	5	Specify the inclusion and exclusion criteria for the review and how studies were grouped for the syntheses.	Page 8 (line 184)
Information sources	6	Specify all databases, registers, websites, organisations, reference lists and other sources searched or consulted to identify studies. Specify the date when each source was last searched or consulted.	Page 7 (lines 160-162)
Search strategy	7	Present the full search strategies for all databases, registers and websites, including any filters and limits used.	Page 7 (lines 162-163)
Selection process	8	Specify the methods used to decide whether a study met the inclusion criteria of the review, including how many reviewers screened each record and each report retrieved, whether they worked independently, and if applicable, details of automation tools used in the process.	Page 7-8 (lines 164-176)<br>Page 8 (lines 184-191)
Data collection process	9	Specify the methods used to collect data from reports, including how many reviewers collected data from each report, whether they worked independently, any processes for obtaining or confirming data from study investigators, and if applicable, details of automation tools used in the process.	Page 8 (line 190)<br>Page 9 (lines 197-206)
Data items	10a	List and define all outcomes for which data were sought. Specify whether all results that were compatible with each outcome domain in each study were sought (e.g. for all measures, time points, analyses), and if not, the methods used to decide which results to collect.	N/A
	10b	List and define all other variables for which data were sought (e.g. participant and intervention characteristics, funding sources). Describe any assumptions made about any missing or unclear information.	N/A
Study risk of bias assessment	11	Specify the methods used to assess risk of bias in the included studies, including details of the tool(s) used, how many reviewers assessed each study and whether they worked independently, and if applicable, details of automation tools used in the process.	N/A
Effect measures	12	Specify for each outcome the effect measure(s) (e.g. risk ratio, mean difference) used in the synthesis or presentation of results.	N/A
Synthesis methods	13a	Describe the processes used to decide which studies were eligible for each synthesis (e.g. tabulating the study intervention characteristics and comparing against the planned groups for each synthesis (item #5)).	N/A

For peer review only - http://bmjopen.bmj.com/site/about/guidelines.xhtml

PRISMA 2020 Checklist

Section and Topic	Item #	Checklist item	Location where item is reported
	13b	Describe any methods required to prepare the data for presentation or synthesis, such as handling of missing summary statistics, or data conversions.	N/A
	13c	Describe any methods used to tabulate or visually display results of individual studies and syntheses.	N/A
	13d	Describe any methods used to synthesize results and provide a rationale for the choice(s). If meta-analysis was performed, describe the model(s), method(s) to identify the presence and extent of statistical heterogeneity, and software package(s) used.	N/A
	13e	Describe any methods used to explore possible causes of heterogeneity among study results (e.g. subgroup analysis, meta-regression).	N/A
	13f	Describe any sensitivity analyses conducted to assess robustness of the synthesized results.	N/A
Reporting bias assessment	14	Describe any methods used to assess risk of bias due to missing results in a synthesis (arising from reporting biases).	N/A
Certainty assessment	15	Describe any methods used to assess certainty (or confidence) in the body of evidence for an outcome.	N/A
**RESULTS**			
Study selection	16a	Describe the results of the search and selection process, from the number of records identified in the search to the number of studies included in the review, ideally using a flow diagram.	Page 8 (line 175 (figure 3))
	16b	Cite studies that might appear to meet the inclusion criteria, but which were excluded, and explain why they were excluded.	N/A
Study characteristics	17	Cite each included study and present its characteristics.	N/A
Risk of bias in studies	18	Present assessments of risk of bias for each included study.	N/A
Results of individual studies	19	For all outcomes, present, for each study: (a) summary statistics for each group (where appropriate) and (b) an effect estimate and its precision (e.g. confidence/credible interval), ideally using structured tables or plots.	N/A
Results of syntheses	20a	For each synthesis, briefly summarise the characteristics and risk of bias among contributing studies.	N/A
	20b	Present results of all statistical syntheses conducted. If meta-analysis was done, present for each the summary estimate and its precision (e.g. confidence/credible interval) and measures of statistical heterogeneity. If comparing groups, describe the direction of the effect.	N/A
	20c	Present results of all investigations of possible causes of heterogeneity among study results.	N/A
	20d	Present results of all sensitivity analyses conducted to assess the robustness of the synthesized results.	N/A

For peer review only

PRISMA 2020 Checklist

Section and Topic	Item #	Checklist item	Location where item is reported
Reporting biases	21	Present assessments of risk of bias due to missing results (arising from reporting biases) for each synthesis assessed.	Page 19 (lines 414-416)
Certainty of evidence	22	Present assessments of certainty (or confidence) in the body of evidence for each outcome assessed.	N/A
**DISCUSSION**			
Discussion	23a	Provide a general interpretation of the results in the context of other evidence.	Page 17-19 (lines 369-405)
	23b	Discuss any limitations of the evidence included in the review.	N/A
	23c	Discuss any limitations of the review processes used.	Page 19 (lines 414-416)
	23d	Discuss implications of the results for practice, policy, and future research.	Page 15 (lines 365-366 (Table 1))
**OTHER INFORMATION**			
Registration and protocol	24a	Provide registration information for the review, including register name and registration number, or state that the review was not registered.	Not registered
	24b	Indicate where the review protocol can be accessed, or state that a protocol was not prepared.	https://systematicreviewsjournal.biomedcentral.com/articles/10.1186/s13643-021-01652-2
	24c	Describe and explain any amendments to information provided at registration or in the protocol.	N/A
Support	25	Describe sources of financial or non-financial support for the review, and the role of the funders or sponsors in the review.	Page 21 (lines 471-478)
Competing interests	26	Declare any competing interests of review authors.	Page 22 (line 481)
Availability of data, code and other materials	27	Report which of the following are publicly available and where they can be found: template data collection forms; data extracted from included studies; data used for all analyses; analytic code; any other materials used in the review.	Page 6 Supplementary file 1 (lines 134-135)<br>Page 7 Supplementary file 2 (lines 157-158)<br>Page 8 Supplementary file 3 (line 184)<br>Page 10 Supplementary file 4 (line 243-244)

From: Page MJ, McKenzie JE, Bossuyt PM, Boutron I, Hoffmann TC, Mulrow CD, et al. The PRISMA 2020 statement: an updated guideline for reporting systematic reviews. BMJ 2021;372:n71. doi: 10.1136/bmj.n71

For more information, visit: http://www.prisma-statement.org/

For peer review only

BMJ Open

Factors influencing decisions about whether to participate in health research by people of diverse ethnic and cultural backgrounds: a realist review.

Journal:	*BMJ Open*
Manuscript ID	bmjopen-2021-058380.R1
Article Type:	Original research
Date Submitted by the Author:	20-Jan-2022
Complete List of Authors:	Hoverd, Eleanor; University of Warwick<br>Hawker-Bond, George; Oxford University, Nuffield Department of Clinical Neurosciences,<br>Staniszewska, Sophie; University of Warwick, Warwick Medical School<br>Dale, Jeremy; University of Warwick, Warwick Medical School
**Primary Subject Heading**:	Health services research
Secondary Subject Heading:	Ethics
Keywords:	Health policy < HEALTH SERVICES ADMINISTRATION & MANAGEMENT, MEDICAL ETHICS, Clinical trials < THERAPEUTICS

SCHOLARONE™
Manuscripts

I, the Submitting Author has the right to grant and does grant on behalf of all authors of the Work (as defined in the below author licence), an exclusive licence and/or a non-exclusive licence for contributions from authors who are: i) UK Crown employees; ii) where BMJ has agreed a CC-BY licence shall apply, and/or iii) in accordance with the terms applicable for US Federal Government officers or employees acting as part of their official duties; on a worldwide, perpetual, irrevocable, royalty-free basis to BMJ Publishing Group Ltd ("BMJ") its licensees and where the relevant Journal is co-owned by BMJ to the co-owners of the Journal, to publish the Work in this journal and any other BMJ products and to exploit all rights, as set out in our licence.

The Submitting Author accepts and understands that any supply made under these terms is made by BMJ to the Submitting Author unless you are acting as an employee on behalf of your employer or a postgraduate student of an affiliated institution which is paying any applicable article publishing charge ("APC") for Open Access articles. Where the Submitting Author wishes to make the Work available on an Open Access basis (and intends to pay the relevant APC), the terms of reuse of such Open Access shall be governed by a Creative Commons licence – details of these licences and which Creative Commons licence will apply to this Work are set out in our licence referred to above.

Other than as permitted in any relevant BMJ Author's Self Archiving Policies, I confirm this Work has not been accepted for publication elsewhere, is not being considered for publication elsewhere and does not duplicate material already published. I confirm all authors consent to publication of this Work and authorise the granting of this licence.

For peer review only

TITLE

Factors influencing decisions about whether to participate in health research by people of diverse ethnic and cultural backgrounds: a realist review.

Corresponding Author: Mrs. Eleanor Hoverd, NIHR Clinical Research Network West Midlands, Room A117, Warwick Medical School, Warwick University, Gibbet Hill Rd, Coventry CV4 7AL, England eleanor.hoverd@nihr.ac.uk

Co-authors:

Dr. George Hawker-Bond, MChem (Hons) Chemistry, MBChB in Medicine and Surgery, Nuffield Department of Clinical Neurosciences, University of Oxford, Oxford, England george.hawker-bond@medsci.ox.ac.uk

Professor Sophie Staniszewska, DPhil (Oxon), BSc (Hons), Warwick Research in Nursing, Warwick Medical School, Warwick University, Coventry, England Sophie.Staniszewska@warwick.ac.uk

Professor Jeremy Dale, Unit of Academic Primary Care, Warwick Medical School, Warwick University, Coventry, England jeremy.dale@warwick.ac.uk

Word count 4806

ABSTRACT

Objective To develop and refine a programme theory that explains factors that influence decisions to take part in health research by people of diverse ethnic and cultural backgrounds.

Design Realist review following a sequence of five steps: 1) scoping search and identification of programme theory 2) evidence searching 3) critical appraisal and data extraction 4) organisation of evidence 5) refinement of programme theory.

Eligibility criteria Documents (including peer-reviewed articles, grey literature, websites, reports, and conference papers) either full text, or a section of relevance to the over-arching research question were included.

Analysis Literature was searched iteratively to develop and test theories that explain how and why a decision to participate in health research is made, based on understanding and experience of informed consent. Key stakeholders helped to refine and finalise programme theory. Data from eligible documents was extracted to build understanding of the factors that influence decision-making. Data was mapped to create a data matrix according to Context (C), Mechanism (M), Outcome (O), Configurations (C) (CMOCs) for the process of informed consent, to aid interpretation and produce a final programme theory.

Results 566 documents were screened and 71 included. The final programme theory was underpinned by CMOCs on processes influencing decisions to take part in research. Key findings indicate the type of infrastructure required, e.g., resources, services and policies, to support inclusion in health research, with a greater need to increase the social presence of researchers within communities, improve cultural competency of individuals and organisations, reduce the complexity of participant information, and provide additional resources to support adaptive processes and shared decision making.

For peer review only - http://bmjopen.bmj.com/site/about/guidelines.xhtml

Conclusion The review indicates the need for a more inclusive research infrastructure that facilitates diverse participation in health research through incorporating adaptive processes that support shared decision making within the informed consent process and in the conduct of research projects.

For peer review only

For peer review only - http://bmjopen.bmj.com/site/about/guidelines.xhtml

ARTICLE SUMMARY

Strengths and limitations of this study

·Using realist methods to explore the contexts and mechanisms of the complexities affecting the informed consent process in health research, for people of diverse ethnic and cultural populations, allowed new insight to emerge.

·There is a paucity of evidence around the informed consent process in health research with people of diverse ethnic and cultural backgrounds, limiting the evidence from which to extract data from.

·Relevance and rigour were increased through the involvement of National Institute for Health Research (NIHR) (Public) Research Champions, healthcare professionals and clinical academics in refining the programme theory.

·Owing to the COVID-19 pandemic, stakeholder involvement in the programme was conducted remotely, enabling access from a wider geographical spread of stakeholders.

·Whilst the review was undertaken in the context of the UK health care system, the programme theory is likely to have wider applicability.

Keywords: realist review, informed consent, under-served, ethnic minority, health research

For peer review only - http://bmjopen.bmj.com/site/about/guidelines.xhtml

INTRODUCTION

People who have poorer health outcomes, and have greater needs are currently under-served by the way in which health research is accessed and delivered.[1] Lack of diversity of participants taking part in health research, poses a serious challenge both within the UK and globally, around how to address and develop a more inclusive health research system so that under-served populations can benefit. It has been argued that the informed consent process may be contributing to inequalities in health research through a lack of flexibility in the process.[2-10] The informed consent process in health research aims to provide patients and the public with the information they require to make a voluntary decision about participation in health research. In May 2019, the International Council for Harmonisation (ICH)[11] drafted the *General Considerations for Clinical Studies E8 (R1)* suggesting that a "*one size fits all approach*" to studies should be avoided. Hence, it is important to unravel the factors that may affect the decision to take part in health research by these populations, to develop a system that is more inclusive.

Evidence suggests that the informed consent process, including the language used within it, creates a serious barrier to access and participation.[8,12-17] Several systematic reviews highlight common barriers to the informed consent process and indicate the need for further research to determine how information is delivered and what information people need, to make an informed decision about participation in health research.[18-20] There is less focus in the literature around what works, to inform research design and delivery. It is a critical issue that requires considerable improvement to develop a more inclusive health research system.

Conducting a realist review of the informed consent process in health research with under-served populations, is a well-suited method for exploring such a complex intervention[21] to explain how the informed consent process may work for whom, how and under what

For peer review only - http://bmjopen.bmj.com/site/about/guidelines.xhtml

circumstances. The over-arching research question for this review is: What contextual factors influence the decision to take part in health research in under-served populations?

The term under-served is preferred when referring to groups or communities who are under-represented in health research e.g., marginalised groups, and is also used to describe geographical locations such as rural or low-income countries, although a single definition is not found within the literature.[1] There are a number of intersecting factors that contribute to groups being under-served; such as demographic, socioeconomic, health status and disease-specific status, leading to disadvantage and discrimination.[1] However, being under-served is also likely to be context-specific, with key characteristics related to trial design influencing the ability to participate.[1] For example, the informed consent process is recognised as a specific barrier to inclusion in health research by under-served populations.[1]

Objectives were as follows:

1. To undertake a realist review to identify the contexts and mechanisms that affect the informed consent process and the decision to participate in health research in under-served populations .
2. To draw on the review's programme theory to develop guidance that addresses barriers to participating in health research in under-served populations.

METHODS

In this study, we undertook a realist review to produce a programme theory to explain the informed consent process, and hence provide actionable recommendations for policymakers and health researchers to inform the development of inclusive health research. The review process followed the steps as laid out in the protocol[22] and was conducted and reported in accordance with RAMESES (Realist and Meta-narrative Evidence Syntheses : Evolving Standards) guidance and publication standards, as well as providing a rationale for selecting

For peer review only

this approach.[23] RAMESES guidance has been produced to provide initial reporting standards for realist reviews and evaluations.[23]

Following a realist analytical process, Context, Mechanism, Outcome, Configurations (CMOCs) were built by EH. These were shared and discussed at meetings with the review team and stakeholders whereby feedback was encouraged which contributed to further refinement and establishing a long list of CMOCs. The CMOCs conceptualise key features that support explanation and understanding of complex programmes and create a model that describes how mechanisms are activated, for who and under what circumstances, which affects outcomes.[23] Summaries were developed after each meeting to support reflection and understanding with stakeholders. Diagrams were constructed and refined to support explanation of the findings.

Patient Public Involvement

The National Institute for Health Research (NIHR) funds health and social care research in the UK and advocates for patient and public involvement (PPI) throughout all stages of research. To support PPI, the NIHR have introduced the role of NIHR (Public) Research Champions who are patients or members of the public, and volunteer to raise awareness of health and social care research as well as help researchers to understand the participant experience.[24] Six NIHR (Public) Research Champions were involved in this review, helping to clarify the scope of the review, contributing to the development of programme theory and developing recommendations. Three of the NIHR (Public) Research Champions were from populations of diverse ethnic and cultural backgrounds.[24] Their involvement is discussed in more detail, below (step 1).

Development of an initial programme theory (step 1)

In order to develop an initial programme theory, a scoping search (see online supplementary file 1 Scoping searches) was carried out based on search terms centred on the intervention

For peer review only - http://bmjopen.bmj.com/site/about/guidelines.xhtml

under study (informed consent) with under-served populations in health research, to explore key literature in this field. This allowed us to explore the available literature and to create some initial boundaries.[25] Theoretical concepts and evidence that would contribute to explaining different outcomes; for example, positive (understood study information) or negative experiences (excluded from taking part). A theoretical concept map was created (figure 1) identifying higher-level, abstract theories which provided a basis to discuss some of the larger complexities of informed consent with the stakeholder group in combination with the empirical evidence from the scoping search, as a way of supporting, contradicting , or modifying the programme theory and to refine the purpose of the review.[25]

The initial scoping search resulted in 16 documents selected based on relevance to the over-arching research question.[4,26-37] These were examined, with codes applied to label CMOCs, concepts and theories, which were helpful in building the initial programme theory. These CMOCs were then collated, a long list of CMOCs drawn-up and then developed into a diagram, to create a visualisation of the initial programme theory (figure 2). This enabled identification of key theories for further exploration.

Stakeholder involvement

Stakeholders included NIHR (Public) Research Champions, health professionals (research nurses, a dietician, physiotherapists and a general practitioner) and clinical academics who were openly invited to participate via the review team's associated Medical School and NIHR Clinical Research Network (CRN). A total of six meetings were held virtually (three with NIHR (Public) Research Champions and three with the mixed group of health professionals and clinical academics). Stakeholders were geographically widespread and thus virtual meetings enabled easier access than had meetings been held face to face. At each stakeholder meeting, background information was provided, including an explanation of realist methodology. The facilitated discussions covered under-served populations; initial

For peer review only

For peer review only - http://bmjopen.bmj.com/site/about/guidelines.xhtml

programme theory; analysis; results and recommendations; and stakeholder feedback and advice.

Meetings were organised to provide perspectives and expert opinions around issues relevant to building programme theory, insights into the ways in which social structure may influence the informed consent process, as well as identifying any pertinent documents of relevance.[21] Stakeholder input is described in the protocol.[22]

Evidence searching (step 2)

Literature searches were conducted iteratively and conducted between May through August 2020 (see online supplementary file 2 Searches) . The initial search used the databases EMBASE, Medline, Web of Science and Psych Info, in addition to manual-searching using Google and Google Scholar. The search strategy was refined for each database , aiming to provide a broad enough review for further building of programme theory.

Following the initial search, 468 citations were identified following removal of duplicates, with 54 citations remaining following 1st and 2nd level screening by EH and G H-B. As synthesis progressed, key theories were emerging around the informed consent process with people of diverse ethnic and cultural backgrounds, with most of literature from the US. Therefore, following discussion with stakeholders and the review team, it was agreed synthesis would focus on a smaller number of key theories with additional searches required to test and refine programme theory, focussing on a "*conceptually rich*"[38] sub-group of literature to produce recommendations relevant to the UK health research system. The reason for limiting the subsequent searches to test theories within the UK literature was because an inclusion policy is lacking, and with a free , publicly funded health care system, context and resources may differ in comparison to other geographical locations, thus affecting mechanisms and outcomes. A flow diagram of the iterative searching process is displayed in figure 3, based on RAMESES guidelines.[23]

For peer review only - http://bmjopen.bmj.com/site/about/guidelines.xhtml

A key consideration of a realist review is to reflect on contextual factors of the social system(s) and how this affects the working of the intervention.[21] In developing the initial programme theory, we sought to view the informed consent process through the larger social system at micro (individual), meso (organisational, institutional) and macro (policy) levels, where interactions between individuals and levels can be understood better.[39] This was aimed at bringing about recommendations for change.[20]

Selection and appraisal of documents (step 3)

Broad inclusion and exclusion criteria (see online supplementary file 3 Inclusion/Exclusion criteria) were applied in the selection and appraisal process, each document was assessed for relevance and rigour by EH and G H-B, independently.[40] Relevance and rigour were assessed in parallel. Decisions were based on whether a document was relevant to the over-arching research question and what it contributed to the review. Rigour was determined by assessing methodological rigour and whether a document included important information that may help with interpretation, as well as providing sufficient detail to determine trustworthiness.[23] Quality of data was noted in data extraction forms and considered during analysis and synthesis. All relevant data was used to build the arguments that supported the final programme theory, including systematic and literature reviews.[25] Two papers included in the review are noted to be included in a systematic review, but do not impact upon the review in terms of the importance given to CMOC development.[6,41] Documents identified as being helpful in developing, refuting and refining the programme theory were sought, although the number of iterations and length of time spent on this was limited due to the timescale of the review.[25]

EH screened all documents identified through searches, by title and abstract. Second reviewer G H-B screened a random 10% of the documents.[42] To establish inter-reviewer agreement,

For peer review only - http://bmjopen.bmj.com/site/about/guidelines.xhtml

a kappa measure of k>0.8 was pre-determined, with a resultant kappa measure of 0.90 following second level screening by EH and G H-B .

Data analysis and synthesis (step 4)

A combination of annotations and notes, along with a data extraction form was used to classify information from documents about potential candidate theories, through noting CMOC concepts.[20] A data extraction form was adapted from Rycroft-Malone[43] to record characteristics of documents and whether evidence was good enough and relevant to include in the synthesis. This included author, year, source, issues of sample size, data collection and data analysis, and interpretations made, characteristics of theoretical concepts and the impact these characteristics may have on the informed consent process (e.g., characteristics of appropriateness of informed consent and how these characteristics may impact on the decision to take part in research). The data extraction form was tested on the first three documents to confirm its applicability and usefulness.

Refinement and validation of programme theory (step 5)

In step 5, both data and annotations were combined. Data synthesis was undertaken by EH through mapping data to create a data matrix according to CMOCs which helped with interpretations and in developing theory.[23] Contexts were themed according to the level of the social system within which they related to.[39] Through noting common features, or relationships and identifying any underlying concepts, recurring themes were identified and patterns in the evidence were tested.

Recurring contexts and outcomes were explained through mechanisms. For example, within the included texts, the design of some research studies were reported as resulting in exclusion of people of diverse ethnic and cultural backgrounds, leading to mistrust, feelings of being exploited and a belief that academic communities are not committed to understanding the needs and experiences of people from diverse ethnic and cultural communities.[5,37,45-46]

For peer review only - http://bmjopen.bmj.com/site/about/guidelines.xhtml

During data synthesis the aim was to provide an explanation of these contexts and outcomes through the identification of mechanisms. Further studies were sought to test the various elements of programme theory. Synthesis results were shared and discussed at stakeholders' meetings, to ensure validity and consistency of the interpretations made. The extraction of data, analysis and synthesis process was iterative with documents repeatedly read at various points throughout the process.

An overview of theories is described in the results section, through a narrative of how the informed consent process works, for whom, how and under what circumstances based upon the evidence in this review. References are made to CMOCs , including some quotations to draw out key aspects of CMOCs.

Development of recommendations (step 6)

Recommendations were developed with stakeholders through presenting the findings and a narrative of the programme theory through a virtual meeting. EH led the remote discussion leading to agreement upon a set of practical recommendations aimed at government agencies, Research Ethics Committees (RECs), researchers, and health professionals. Input from stakeholders was intended to ensure their practicality.

RESULTS

Document characteristics

In total, seventy-one documents published between 2005 and 2020 were included from 19 countries with the majority from the USA (37%), UK (24%) and Africa (13%). The date range of the sources was from 2004 to 2020. The source type was mixed including discussion papers and opinion pieces (17, 24%), qualitative research (27, 38%), literature reviews (4, 6%) , mixed methods research (2, 3%), systematic reviews (5, 8%), quantitative research (9, 13%), guidelines (2, 3%), case study (1, 1%), book chapter (1, 1%), audit (1, 1%), report (1, 1%) and a working paper (1, 1%).

For peer review only - http://bmjopen.bmj.com/site/about/guidelines.xhtml

Context Mechanism Outcome Configurations (CMOCs)

The key themes that emerged related to inter-relationships; organisational and individual cultural competency and adaptive processes. Patterns in Mechanisms were identified as can be seen on the long list of 29 CMOCs used to develop programme theory (see online supplementary file 4 CMOC Long List). These are discussed below making reference to the associated, numbered CMOCs and selected quotations from documents which help to explain the theories.

Inter-relationships

Developing trusting relationships between researchers and under-served communities is important. Key family members, community, or faith leaders and local healthcare staff may influence a potential participant's decision to take part (CMOC 3, CMOC 5, CMOC 11).[3,47,48] The approach to identifying key individuals must be done sensitively with clear information provided about what participation involves (CMOC 1, CMOC 7, CMOC 9, CMOC 18, CMOC 26).[3,6,17-49] This can lead to increased autonomy, trust and a positive view towards health research with the key individuals becoming a trusted point of information, thus helping to improve knowledge about health research, increasing the awareness and confidence of communities to participate in research.[3]

> *…research assistants attempted to immerse themselves in the activities of the diabetes clinic…*[6] (P.7) (CMOC 1).

Researcher-matching may help (CMOC 1, CMOC 4) as it provides some choice for the potential participant as to whom they work with[7,50] and can enable more effective and positive experiences. This has been used in trials on diabetes, mental health, and cardiovascular disease with participants of South Asian heritage and Black British, African, and African-Caribbean heritage.[51,52] There does not appear to be one definition of researcher-matching; examples include: an interviewer speaking the same language as the

For peer review only - http://bmjopen.bmj.com/site/about/guidelines.xhtml

participant conducts interviews; a health professional who speaks the same language as participant conducts intervention; an interpreter is present to support delivery of the intervention; trained multi-lingual facilitators; ethnically matched outreach team delivers the intervention; research assistants who speak the same language; and culturally adapted interventions.[2,9,17,37,52-59,53] The cost implications of this approach were rarely reported, with only one study discussing the impact and challenges of training facilitators and the complexities of translating material.[9] The perceptions of having interviews or focus groups conducted by someone of the same ethnicity is under-explored and viewed by some researchers as not as essential as language, gender, technical and interpersonal skills which may be more important.[9] There was some suggestion that researcher-matching should be used with caution as there is a risk that this may lead to researchers lacking cultural competence.[60] There is also a risk when using interpreters who have been trained to facilitate qualitative interviews that the richness of data could be compromised.[61]

Family relationships may trigger a positive or negative experience of the decision-making process (CMOC 5, CMOC 11).[2-4,62] Identification of key family /community members involved in decision-making process can determine whether someone takes part and conversely can improve the choice through improved understanding (CMOC 5).[2-3,60,62]

Organisational and individual cultural competency

There is considerable evidence to support the importance of delivering services with cultural competence (CMOC 2, CMOC 9).[3,5] Cultural competence is defined as the way in which behaviours, attitudes, and policies of a system, or amongst individuals, such as health care professionals, enables a service to operate effectively amongst diverse cultural situations.[63] Developing cultural competency and leadership helps to develop a capable, skilled research workforce (CMOC 1)[3,5,7,10] protecting the autonomy of potential participants,

For peer review only - http://bmjopen.bmj.com/site/about/guidelines.xhtml

encouraging reflection on the individual's life situation, and developing awareness of different cultures.[5]

Contextual factors such as the life situation of some populations (e.g., migrant communities) can influence the decision-making process in health research due to

> *...fear of identification and reporting to authorities*[5](P.13) (CMOC 11).

Whilst cultural competence is evidenced to improve the responsiveness and appropriateness in the way in which health research is delivered, as well as reducing health disparities, it requires further exploration that goes beyond the scope of this review.[3,5,7,10,17] There is some evidence to suggest that cultural competency conflates language, geographical origin, ethnicity and race, potentially diluting the identity of individuals and communities.[64] The term has caused considerable debate since the introduction of the concept in the 1980's[63] with some asserting that it is not possible to be competent in other individuals' cultures.[59,65] However, cultural competence has been considered vital for engaging with diverse ethnic and cultural communities to ensure accurate information is shared appropriately to instil confidence in the safety and efficacy of COVID-19 vaccines.[66]

Cultural competency when positioned within a human rights framework is viewed positively as a way of operationalising respect, providing a service that is culturally appropriate.[64,67]

Adaptive processes

Providing small bitesize participant information about an available health research study, in local languages, with local cultural context supports understanding.[5,17,68] In addition to this, co-production and more community-based participatory approaches may encourage wider participation, offering a way to empower individuals and communities who are under-served in health research.[69] It has been demonstrated that engagement with people of African-Caribbean heritage in the development of a culturally-adapted family intervention for people with schizophrenia can be achieved by taking a two-way approach, whereby

For peer review only - http://bmjopen.bmj.com/site/about/guidelines.xhtml

researchers become integrated within communities and individuals from communities are able to access training and support around health research to increase capability as part of a community based participatory research (CBPR) approach, this has been demonstrated.[71] Taking these approaches improves accountability and makes provided information more relevant.

> *…members of target populations should be involved in the overall design of research…*[41] (P.5) (CMOC 9, CMOC 14).

In addition, it may be helpful to offer a range of materials (e.g., written, oral, audio, and multi-media) to ensure concepts are understood (CMOC 2, CMOC 12).[3,28] Providing study information in various formats can promote feelings of independence, leading to consent being given freely, serving more respect for cultural differences (CMOC 2, CMOC 14).[6,7,71]

> *...this way you won't need to depend on your children anymore* [6](P6*)* (CMOC 3).

Some groups lack an agreed written form of their main language, and alternative formats are useful in this respect (CMOC 2, CMOC 12).[6-7]

This creates trust and ensures dignity.[3,5-6,10,17,51]

Institutional barriers

When the needs (e.g., cultural, linguistic or historical) of people from diverse ethnic and cultural backgrounds are misrepresented or ignored [3,5,72] (CMOC 6, CMOC 8, CMOC 13) this results in: discriminatory outcomes (e.g., exclusion due to eligibility criteria) and enforces inappropriate ethical, legislative, scientific guidelines and recruitment processes (CMOC 5, CMOC 13).[3,5,8] It has been recognised that individuals from diverse ethnic and cultural backgrounds are under-represented in commissioning and making funding decisions about health research, and on editorial boards of journals (CMOC 13).[9]

For peer review only - http://bmjopen.bmj.com/site/about/guidelines.xhtml

Study designs can restrict access to health research through creating eligibility criteria that excludes populations from taking part, usually due to a language barrier.[3]

> *...many studies require participants to have the ability to understand and speak English which excludes older people from ethnic minority communities and/or migrants*[3] (P.6) (CMOC 5, CMOC 9, CMOC 12, CMOC 13).[3,72-73]

This results in under-representation and may be perceived as institutional racism (CMOC 5, CMOC 9, CMOC 12, CMOC 13).[2,8,58,73]

> *…dearth of research with ethnic minority communities in the UK is a reflection of the lack of influential policy…*[3](P.9) (CMOC 6, CMOC 13).

Evidence from the United States suggests that institutional racism is not easily defined and encompasses not only individual prejudices, but prejudices deeply embedded within the law, practice, economy, culture and society which result in unequal access to healthcare, including health research.[74] The role of institutional racism in health research may be fuelled by the costs for translation and additional culturally sensitive resources affecting the chances of receiving funding for grant applications.[52] However, there is little evidence available on the extent to which institutional racism occurs in health research. A systematic review found that it is a concept that is rarely explicitly named in abstracts and titles and rarely engaged with; lack of acknowledgement of its existence could be holding back discovery within this field.[64-75]

Programme Theory

A resultant programme theory was produced following the five steps shown in figure 4. The programme theory informed discussions with stakeholders to develop recommendations and actions (Table 1) for individuals, organisations, institutions and policymakers to improve the informed consent process.

Table 1. Recommendations

For peer review only - http://bmjopen.bmj.com/site/about/guidelines.xhtml

Recommendation	Action required
**1. Policymakers, organisations, institutions and individuals need to take responsibility and address institutional racism, identifying and tackling issues that contribute to this (e.g., lack of culturally adapted information, practices that may be contributing to institutional racism.)**	This requires:<br><br>- leadership<br>- identification of best practice<br>- open discussion of culturally sensitive research ethics throughout the social system (e.g., values, beliefs and practices)<br><br>Organisations and institutions should inform and educate their staff about institutional racism, what it is and how to address it.
**2. Build relationships with communities from diverse cultural and ethnic backgrounds to provide opportunities for Patient and Public Involvement (PPI) and empower individuals so that study designs become more relevant and culturally appropriate.**	Employ more staff from diverse ethnic and cultural backgrounds in academic institutions and health research organisations.<br><br>Ethics committees should seek community members from diverse ethnic and cultural backgrounds.<br><br>Provide cultural competency training for all involved in health research.<br><br>Identify local champions and/or leads to raise awareness of cultural competency in health research.
**3. Develop tailored informed consent in health research that offers choices (e.g., written, audio, multi-media), which may require additional resources.**	Ensure greater Patient and Public Involvement (PPI) by people of diverse ethnic and cultural backgrounds in study designs.
**4. Organisations and institutions need to develop cultural competence within them,**	This requires cultural competence training that covers capability, knowledge and

For peer review only - http://bmjopen.bmj.com/site/about/guidelines.xhtml

**increasing capability, knowledge and awareness of other cultures.**	awareness in relation to research participation.<br>All who are involved in health research should undertake cultural competency training.

DISCUSSION

Statement of principal findings

This realist review of 71 sources summarises and expands upon the existing evidence around decision-making and participation in health research through focussing upon the context and mechanisms surrounding decision-making and understanding. The evidence base on participation in health research has tended to focus on the psychosocial drivers of participation, but use of a realist approach enabled greater consideration of contextual factors that influence whether participation occurs.

Through deeper exploration using iterative literature searching, data emerged that indicated the use of complex study documentation e.g., patient information leaflets, communication barriers, lack of social presence in the community by researchers and a lack of diversity amongst researchers as key realist mechanisms which led to an explanation of the interaction with context to influence outcomes.[2,8,54,72] Whilst the review team itself was not ethnically diverse, the stakeholder group were ethnically, professionally and organisationally diverse. EH is part of an Equality, Diversity, Inclusion working group which supported engagement with patient and public stakeholders from diverse cultural and ethnic backgrounds.

At the individual level (micro) level, empowerment through access to resources, such as culturally adapted information, and the opportunity to be involved in the design of health research form a significant aspect of the programme theory. At the interpersonal and

For peer review only - http://bmjopen.bmj.com/site/about/guidelines.xhtml

institutional (meso) levels, enabling shared decision making can result in positive outcomes through approaches such as co-production; building community relationships and provision of researcher-matching and/or culturally competent researchers provides assurance and increases trust.

The differences and similarities between the use of the term shared decision making in clinical care and within a health research context would benefit from additional exploration of the literature. The term shared decision making is defined by the UK's National Institute for Health and Care Excellence (NICE) shared decision making collaborative as:

> *a process in which clinicians and patients work together to select tests, treatments, management or support packages, based on clinical evidence and the patient's informed preferences. It involves the provision of evidence-based information about options, outcomes and uncertainties, together with decision support counselling and a system for recording and implementing patients' informed preferences.*[78-79]

It is of key importance in protecting patient/participant autonomy and voluntariness.[76-79] In the context of this review, shared decision making is part of the informed consent process and requires establishing understanding by the potential participant, discussion of the risks and benefits, as well as clarifying the consequences of different options available.[77] Shared decision making is also a vital element of co-production, or the involvement of patients and the public in shaping research projects.[80] In some instances, shared decision making can result in negative, or positive outcomes; for example, the hierarchies within some families may control decision-making through preventing or granting permission for family members to take part in health research.[6,9] More positive, shared decision making occurs when researchers spend time with communities, providing opportunities for informal discussions about research, building trust whilst developing cultural sensitivity to the "*norms and values*" of particular groups.[26]

For peer review only - http://bmjopen.bmj.com/site/about/guidelines.xhtml

Resources at the macro level are lacking, such as the lack of flexibility in approaches to the informed consent process, leading to negative outcomes with needs not being met and exclusion from health research, which may be perceived as institutional racism.[46,52,58,75] The data synthesis together with the stakeholder group meetings framed an overall programme theory in realist terms as:

> *If* organisations, institutions and individuals work together with diverse ethnic and cultural communities and become culturally competent, provide adaptive processes and empower community members to become involved , *then* this will develop and result in trust, better engagement, shared decision making and improved understanding.

The programme theory from this realist review is likely to have wider applicability despite being carried out in the context of the UK health care system. The informed consent process is a universal cornerstone to ethical practice in health research although may vary in the way in which it is carried out. However, rigid study designs and delivery may be contributing to the global issue of under-representation of under-served populations in health research.[18,50] While the critical issues described in this review are likely to be suitable for consideration by health research systems outside of the UK, it is recognised that resources and context differ across research systems, which may limit the transferability, particularly in low-income countries. The recommendations in this realist review requires evaluation in different settings to understand their wider applicability and transferability.

Strengths and weaknesses in relation to other studies

Using a realist approach enabled a wider overview of more hidden factors that affect the decision-making process in health research, regarding the informed consent process with people of diverse ethnic and cultural backgrounds. The realist review has drawn from both peer-reviewed literature (66,93%) and grey literature (5,7%) as well as bringing in expertise

For peer review only - http://bmjopen.bmj.com/site/about/guidelines.xhtml

from the stakeholder group. The lack of literature available on health research in general with people of diverse ethnic and cultural backgrounds is a weakness as more specific evidence about what works, for whom and under what circumstances is urgently needed in order to help researchers design studies that are more inclusive and inform service development.[3,5] It is acknowledged that not all sources may have been discovered throughout this realist review process, in addition to not including the expertise of policymakers due to restrictions on time.

The review was conducted in accordance with RAMESES quality and publication standards to ensure transparency and is viewed as a strength.[19,43]

The involvement of NIHR (Public) Research Champions, healthcare professionals, clinical academics and PPI Lead in refining the programme theory was felt to enhance the review's relevance. Having a second reviewer can increase the number of relevant studies included in a review and therefore this was considered a strength.[5]

Suggestions for further research

From the review, cultural competence emerged as a recurring mechanism within the literature with further gaps in the evidence around : the way in which studies are designed and delivered and how people from under-served populations are involved in this aspect of the research process; institutional racism and its impact on health research; experiences of participation in health research with people of diverse ethnic and cultural backgrounds; the best adaptive informed consent processes, particularly for people of diverse ethnic and cultural backgrounds . In addition to this, further research is required to understand more the full range of requirements that may be needed to develop a more inclusive informed consent process, alongside in-depth enquiry around cultural competence and researcher-matching in relation to outcomes about research participation.

CONCLUSION

For peer review only - http://bmjopen.bmj.com/site/about/guidelines.xhtml

The resultant programme theory explains how, when, why and for whom the processes involved in consenting to participate in health research does and does not work, and leads to suggestions for improvements to achieve greater inclusivity with people of diverse ethnic and cultural backgrounds.

Some aspects of the programme theory (e.g., institutional racism, cultural competency in health research, researcher-matching) need more research to better understand how they affect outcomes. A critical finding is that a research infrastructure that supports and mandates inclusion is needed, as well as additional resources to support adaptive processes, support shared decision making through involving more patients and the public in the design of health research studies. A greater focus on the evidence base developing in the field of global health community engagement, particularly around co-production, may provide valuable insights into how patients and the public from under-served communities can become involved in the design of health research studies.[80]

Main document

Figure 1. Theory Map – Factors influencing decisions about whether to participate in health research with under-served populations – identified from scoping search

Figure 2. Theoretical concepts compounding positively, or negatively, the intervention

Figure 3. Flow diagram of searching

Figure 4. Final programme theory

online supplementary file 1 – Scoping searches

online supplementary file 2 – Searches

online supplementary file 3 – Inclusion/Exclusion criteria

For peer review only - http://bmjopen.bmj.com/site/about/guidelines.xhtml

online supplementary file 4 – CMOC Long List

LIST OF ABBREVIATIONS

NIHR National Institute for Health Research

PPI Patient and Public Involvement

CMOC Context Mechanism Outcome Configuration

UCL IRIS University College London Institutional Research Information System

IHE Institute for Health Equality

DECLARATIONS

Funding statement

This work was supported by a National Institute for Health Research (NIHR), HEE/NIHR ICA Programme Pre-doctoral Clinical Academic Fellowships *grant number* NIHR 300317, as a Masters in Health Research dissertation. Funding for the open access charges for the publication of this protocol was provided by the NIHR Clinical Research Network West Midlands.

SS is part funded by the NIHR Applied Research Collaboration (ARC) West Midlands, the NIHR Health Protection Research Unit (HPRU) Gastrointestinal Infections, and the NIHR HPRU Genomics and Enabling Data.

Disclaimer The views expressed in this publication are those of the author(s) and not necessarily those of the NIHR, HEE, NHS or the UK Department of Health and Social Care.

Competing Interests None declared.

Ethics approval and consent to participate Not applicable**.**

Consent for publication. Not applicable.

For peer review only

For peer review only - http://bmjopen.bmj.com/site/about/guidelines.xhtml

Availability of data and materials. Not applicable.

Competing interests. None declared.

Author contributions EH was the lead reviewer. G H-B completed second reviewer tasks. SS, JD, G H-B critically reviewed and edited the manuscript. EH was responsible for the initial design and drafting of the manuscript. All authors read and approved the final manuscript.

Acknowledgements We thank Samantha Johnson Information Specialist, for critically appraising the search strategy. We would also like to thank the following stakeholders involved in this review for their contribution to improving the quality and relevance of the final programme theory as well as the recommendations: Anne Devrell, Geoff Robson, Ifeanyi Sargeant, Mohini Samini, Dr. Mohammed Shaikh, Richard Stephens, Sandra Prew, Dr. Farhana Lockhat, Dr. Emma Sutton , Zi Liew, Sarah Joshi, Claire Talbot, Jonathan Heffernan-Davies, Sylvia Turner, Mohamed Mooradun.

Patient consent for publication Not required.

Data sharing statement This review presents previously published and publicly available data. Please refer to the reference list and their authors for these research data.

References

1 Witham M, Anderson E, Carroll C, et al. Developing a roadmap to improve trial delivery for under-served groups: results from a UK multi-stakeholder process. *Trials*. 2020. doi.org/10.1186/s13063-020-04613-7 [published First Online 1 Aug 2020]

2 Redwood S, Gill P. Under-representation of minority ethnic groups in research- call for action. *Br J Gen Pract*. 2013. doi:10.3399/bjgp13X668456 [published Jul 2013]

3 Jutlla, K, Raghavan R. Improving the recruitment of Black, Asian and Minority Ethnic (BAME) communities in health and social care research: a

For peer review only - http://bmjopen.bmj.com/site/about/guidelines.xhtml

review of literature 2017.www.dora.dmu.ac.uk/handle/2086/13221 (accessed 28 Jun 2020)

4 Verastegui, E.Consenting of the vulnerable: The informed consent procedure in advanced cancer patients in Mexico. *BMC medical ethics*. 2006 doi:org/10.1186/1472-6939-7-13 [published Online First: 13 December 2006]

5 Vickers T, Craig G, Atkin K. Addressing Ethnicity in Social Care Research *Social Policy and Administration47(3):310 – 326* doi:org/10.1111/j.1467-9515.2012.00851.x [published Online First 24 May 2012]

6 Lloyd, C, Johnson, M, Mughal, S. Securing recruitment and obtaining informed consent in minority ethnic groups in the UK. BMC *Health Serv Res*.2008; 8: 68.doi:org/10.1186/1472-6963-8-68 [published Online First 30 Mar 2008]

7 Masood Y, Bower P, Waheed M, et al.Synthesis of researcher reported strategies to recruit adults of ethnic minorities to clinical trials in the United Kingdom: A systematic review. *Contemp Clin Trials*2019; 78:1-10. doi: 10.1016/j.cct.2019.01.004 [published First Online 8 Jan 2019]

8 Barata P, Guicciardi E, Ahmad F et al.Cross-cultural perspectives on research participation and informed consent. *Social Science & Medicine.*2006;62:479-www.academia.edu/9183270/Cross_cultural_perspectives_on_research_participation_and_informed_consent (accessed 01 Jun 2020)

9 Culley L, Hudson N, Rapport F. Using Focus Groups with Minority Ethnic Communities: Researching Infertility in British South Asian Communities. *Qualitative Health Research*. 2007;17(1):102-112. doi:10.1177/1049732306296506 [published First Online 1 Jan 2007]

10 Smart A, Harrison E. The under-representation of minority ethnic groups in UK medical research. *Ethn Health*. 2017 Feb;22(1):65-82. doi: 10.1080/13557858.2016.1182126 [published First Online 13 May 2016]

11 ICH .ICH E8 General considerations for clinical studies.2019.www.ema.europa.eu/en/ich-e8-general-considerations-clinical-studies (accessed Jun 2021)

12 Verastegui E. Consenting of the vulnerable: the informed consent procedure in advanced cancer patients in Mexico *BMC Medical Ethics.* 2006;7:13 doi:10.1186/1472-6939-7-13 [published First Online 13 Dec 2006]

13 Riesa N, Mansfield E, Sanson-Fisher R. Ethical and legal aspects of research involving older people with cognitive impairment: A survey of dementia researchers in *Australia International Journal of Law and Psychiatry. 2019;68.*doi.org/10.1016/j.ijlp.2019.101534 [published Feb 2020]

14 Smith J. How ethical is ethical research? Recruiting marginalized, vulnerable groups into health services research. *Journal of Advanced Nursing* 2008;62(2), 248–257. doi: 10.1111/j.1365-2648.2007.04567.x [published First Online 3 Apr 2008]

15 Zion D, Briskman L, Loff B. Returning to History: The Ethics of Researchign Asylum Seeker Health in Australia . *The American Journal of Bioethics*.2010;

For peer review only - http://bmjopen.bmj.com/site/about/guidelines.xhtml

10(2). doi.org/10.1080/15265160903469310 [published First Online 3 Feb 2010]

16 Atkin K. Institutional racism, policy and practice. In: Primary Healthcare and South Asian Populations–Meeting the Challenges. Oxford: Radcliffe Medical Press. 2004:1-12

17 Flory J, Emanuel E. Interventions to improve research participants' understanding in informed consent for research: a systematic review 2004. *JAMA* 292(13):1593-601

18 Nishimura A, Carey J, Erwin P. Improving understanding in the research informed consent process: a systematic review of 54 interventions tested in randomised control trials. *BMC Medical Ethics*. [online] 2013*:*14:28 Available from : .doi:10.1186/1472-6939-14-28 (accessed 15 Jun 2020)

19 Montalvo W, Larson E. Participant comprehension of research for which they volunteer: a systematic review. *J Nurs Scholarsh*. [online]2014;46(6):423-31. Available from : doi: 10.1111/jnu.12097. (accessed 30 Jun 2020)

20 Pawson R, Greenhalgh T, Harvey G, et al. Realist Review - a new method of systematic review designed for complex policy interventions. *J Health Serv Res Policy*. 2005; 1:21-34.doi:10.1258/1355819054308530 [published 1 Jul 2005]

21 Pawson, R, Greenhalgh, T, Harvey, G et al. 2004 *Realist Synthesis: An Introduction.* RMP Methods Paper 2/2004. www.researchgate.net/publication/228855827_Realist_Synthesis_An_Introduction/link/0fcfd507f0b7cbb2ce000000/download (accessed May 2020)

22 Hoverd E, Staniszewska S, Dale J. The informed consent process in health research with under-served populations: a realist review protocol. *Syst Rev*. 2021;10: 103. doi.org/10.1186/s13643-021-01652-2 [published First Online 9 Apr 2021]

23 Wong G, Greenhalgh T, Westhorp G. et al. RAMESES publication standards: realist syntheses. *BMC Med.2013;* 11:21 . doi.org/10.1186/1741-7015-11-21[published First Online29 Jan 2013]

24 National Institute for Health Research. Research Champions 2020.www.nihr.ac.uk/patients-carers-and-the-public/i-want-to-help-with-research/research-champions.htm (accessed 20 Apr 2020)

25 Emmel N, Greenhalgh J, Manzano A, et al. *Doing Realist Research*. London: Sage 2018.

26 Calamaro CJ. Culture competence in research: research design and subject recruitment. *J Pediatr Health Care.* 2008 Sep-Oct;22(5):329-32. doi: 10.1016/j.pedhc.2008.05.007 [published 1 Sep 2008]

27 Francis-Graham S, Ekeke N, Nelson C, et al. Understanding how, why, for whom, and under what circumstances opt-out bloodborne virus testing programmes work to increase test engagement and uptake within prison: a rapid-realist review *BMC Health Services Research* .2019;19:152 doi.org/10.1186/s12913-019-3970-z [published First Online 8 Mar 2019]

For peer review only - http://bmjopen.bmj.com/site/about/guidelines.xhtml

28 Garrison, N Sathe, N, Antommaria, A *et al.* A systematic literature review of individuals' perspectives on broad consent and data sharing in the United States. *Genet Med* 2016;18, 663–671. doi.org/10.1038/gim.2015.138 [published First Online 19 Nov 2015]

29 Hill, C Tawiah-Agyemang, S, Odei-Danso, B et al.Informed consent in Ghana: what do participants really understand? *Med Ethics* 2008;34:48–53. doi:10.1136/jme.2006.019059 [published First Online 21 Dec 2007]

30 Hughson J, Woodward-Kron R, Parker A, et al. A review of approaches to improve participation of culturally and linguistically diverse populations in clinical trials *Trials*. 2016 doi:10.1186/s13063-016-1384-3 [published First Online 26 May 2016]

31 Locher J,Bronstein J, Robinson et al. Ethical Issues Involving Research Conducted With Homebound Older Adults The Gerontologist *The Gerontological Society of America;*2006; 46: 2, 160–164. doi.org/10.1093/geront/46.2.160 [published First 1 Apr 2006]

32 Munthe C, Radovic S, Anckarsater H.Ethical Issues in forensic psychiatric research on mentally disordered offenders. *Bioethics*2010;24(1):34-35. doi:10.1111/j.1467-8519.2009.01773.x [published First Online 11 Dec 2009]

33 Mystakidou K ,Panagiotou I. Ethical and practical challenges in implementing informed consent in HIV/AIDS clinical trials in developing or resource-limited countries, SAHARA-J: *Journal of Social Aspects of HIV/AIDS*, 2009;6:2, 46-57. doi.org/10.1080/17290376.2009.9724930 [published First Online 28 Feb 2009]

34 Shepherd, V, Wood, F, Griffith, R et al. Protection by exclusion? The (lack of) inclusion of adults who lack capacity to consent to research in clinical trials in the UK .*Trials.* 2019;20:474. doi.org/10.1186/s13063-019-3603-1[published First Online 5 Aug 2019]

35 Vallely L, Lees S, Shagi C et al. How informed is consent in vulnerable populations? Experience using a continuous consent process during the MDP301 vaginal microbicide trial in Mwanza, Tanzania *BMC Medical Ethics* 2010;11:10 .www.biomedcentral.com/1472-6939/11/10 (accessed Jul 2020)

36 Lakes, K, Vaughan, E, Jones, M.et al. Diverse perceptions of the informed consent process: implications for the recruitment and participation of diverse communities in the National Children's Study. *American journal of community psychology* 2012; 49(1-2), 215–232. doi.org/10.1007/s10464-011-9450-1[published First Online 14 Jun 2011]

37 Folayan M, Haire B, Harrison A, et al.Ethical Issues in Adolescents' Sexual and Reproductive Health Research in Nigeria. *Dev World Bioeth.* 2015;15(3):191-8. doi: 10.1111/dewb.12061[published First Online 9 Jun 2014]

38 Kantilal K, Hardeman W, Whiteside H, et al. Realist review protocol for understanding the real-world barriers and enablers to practitioners implementing self-management support to people living with and beyond

For peer review only - http://bmjopen.bmj.com/site/about/guidelines.xhtml

cancer *BMJ Open* 2020;10. doi: 10.1136/bmjopen-2020-037636 [published First Online 3 Sep 2020]

39 Malek- Ahmadi M. Macro-Level, Meso-Level, and Micro-Level Analysis 2020.www.coursehero.com/sg/introduction-to-sociology/macro-level-meso-level-and-micro-level-analysis/ (accessed Aug 2020)

40 Moore T, McArthur M, Noble-Carr D. More a marathon than a hurdle: towards children's informed consent in a study on safety. *Qualitative Research* 2018;18(1):88-107. doi:10.1177/1468794117700708 [published First Online 2 May 2017]

41 Vickers T, Craig G, Atkin K. Research with black and minority ethnic people using social care services. SSCR methods review, 2012;11. NIHR School for Social Care Research, London, UK.www.eprints.lse.ac.uk/43161/1/SSCR_Methods_Review_11_(lsero).pdf (accessed Apr 2020)

42 Stoll C, Izadi S, Fowler S et al. The value of a second reviewer for study selection in systematic reviews *Research synthesis methods*. 2019;10(4):539-545. doi.org/10.1002/jrsm.1369 [published First Online 4 Jul 2019]

43 Rycroft - Malone J, McCormack B, Hutchinson A. Realist synthesis: illustrating the method for implementation research. *Impl Science*. 2012;doi:10.1186/1748-5908-7-33 [published First Online 19 Apr 2012]

44 Scharff D, Mathews K, Jackson P, et al. More than Tuskegee: understanding mistrust about research participation. *J Health Care Poor Underserved*. 2010;21(3):879-897. doi:10.1353/hpu.0.0323 [published First Online Aug 2010]

45 Kao C, Aranda S, Krishnasamy M, et al. Identifying essential information to support patient decision-making regarding participation in cancer clinical trials: A Delphi study. *Eur J Cancer Care* (Engl). 2018 ;27(6).doi: 10.1111/ecc.12954 [published First Online 17 Sep 2018]

46 Grady, C, Hampson, L, Wallen, G et al. Exploring the ethics of clinical research in an urban community. *American Journal of Public Health,* 2006;96(11). doi.org/10.2105/AJPH.2005.071233 [published First Online 10 Oct 2011]

47 McDougall G, Simpson G, Friend M. Strategies for research recruitment and retention of older adults of racial and ethnic minorities. *J Geront Nurs*. 2015. doi.org/10.3928/00989134-20150325-01 [published First Online 25 Mar 2015]

48 Heywood R, Mackaskill A,Williams K. Informed consent in hospital practice: health professionals' perspectives and legal reflections.*Medical Law Review,* 2010; 18 (2).www.pubmed.ncbi.nlm.nih.gov/20513795/ (accessed May 2020)

49 Mchale J. Innovation, informed consent, health research and the Supreme Court: Montgomery v Lanarkshire - a brave new world? *Health Econ Policy Law*. 2017;12(4):435-452. doi: 10.1017/S174413311700010X. [published First Online 27 Apr 2017]

For peer review only - http://bmjopen.bmj.com/site/about/guidelines.xhtml

50 Treweek S, Forouhi N, Narayan K,et al .COVID-19 and ethnicity:who will research results apply to? *The Lancet* 2020. 395(10242):1955.doi: 10.1016/S0140-6736(20)31380-5 [published First Online 12 Jun 2020]

51 Jolly K, Lip G, Taylor R,et al. Recruitment of ethnic minority patients to a cardiac rehabilitation trial: the Birmingham Rehabilitation Uptake Maximisation (BRUM) study *BMC medical research methodology* 2005;*5*, 18. doi.org/10.1186/1471-2288-5-18 [published First Online 17 May 2005]

52 Hussain-Gambles M, Atkin K, Leese B. South Asian participation in clinical trials: the views of lay people and health professionals. *Health Policy.* 2006 ;77(2):149-65. doi: 10.1016/j.healthpol.2005.07.022.[published First Online 10 Oct 2005]

53 Kleinman A, Benson P. Anthropology in the Clinic: The Problem of Cultural Competency and How to Fix It. *PLoS Med* 2006;3(10). doi.org/10.1371/journal.pmed.0030294 [published First Online 24 Oct 2006]

54 Kirby T. Evidence mounts on the disproportionate effect of COVID-19 on ethnic minorities. *The Lancet Respiratory Medicine*.2020.doi:10.1016/S2213-2600(20)30228-9 [published First Online 8 May 2020]

55 McDougall G, Simpson G, Friend M. Strategies for research recruitment and retention of older adults of racial and ethnic minorities. *J Geront Nurs.* 2015.doi:10.3928/00989134-20150325-01 [published First Online 30 Mar 2015]

56 Ries N, Thompson K, Lowe M. Including People with Dementia in Research: An Analysis of Australian Ethical and Legal Rules and Recommendations for Reform. *J Bioeth Inq.* 2017;14(3):359-374. www. //psycnet.apa.org/record/2017-38470-007 (accessed Apr 2020)

57 Dawson, S, Campbell S, Giles S et al. Black and minority ethnic group involvement in health and social care research: A systematic review. *Health expectations: an international journal of public participation in health care and health policy* 2018;21(1): 3-22. doi:10.1111/hex.12597 [published First Online 15 Aug 2018]

58 Wiles, R, Charles V, Crow G et al. 'Researching researchers: lessons for research ethics. *Qualitative Research*, 2006;6(3): 283–299.www. https://eprints.soton.ac.uk/40739/ (accessed Aug 2021)

59 Hamel L, Penner L, Albrecht T, et al. Barriers to Clinical Trial Enrollment in Racial and Ethnic Minority Patients With Cancer. *Cancer Control.* 2016;23(4):327-337. doi:10.1177/107327481602300404 [published First Online 1 Oct 2016]

60 Chavez V. Cultural humility: Reflections and relevance for CBPR. In Wallerstein, N., Duran, B., Oetzel, J., Minkler, M. (Eds.), Community-based participatory research for health: Advancing social and health equity 2018; 357-362. [doi.org/10.1177/1524839919884912 [published First Online 12 Nov 2019]

For peer review only - http://bmjopen.bmj.com/site/about/guidelines.xhtml

61 Erves J, Mayo-Gamble T, Malin-Fair A, et al. Needs, Priorities, and Recommendations for Engaging Underrepresented Populations in Clinical Research: A Community Perspective. *J Community Health*. 2017 ;42(3):472-480. doi: 10.1007/s10900-016-0279-2. [published First Online 3 Nov 2016]

62 Cheah P, Parker M. Research consent from young people in resource-poor settings. *Archives of disease in childhood.*2014. doi:10.1136/archdischild-2014-307121.[published May 2015]

63 Cross T, Bazron B, Dennis K, et al. Towards a culturally competent system of care.1989;Georgetown University Child Development Center, CASSP Technical Assistance Center, Washington, DC. www. https://files.eric.ed.gov/fulltext/ED330171.pdf (accessed May 2020)

64 Jongen C, McCalman J, Bainbridge R. Health workforce cultural competency interventions: a systematic scoping review. *BMC Health Serv Res* 2018;18(232) . doi.org/10.1186/s12913-018-3001-5 [published First Online 2 Apr 2018]

65 Minkler M, Pies C, Hyde C. Ethical issues in community organizing and capacity building. In Minkler, M. (Ed.), Community organizing and community building for health and welfare .New Brunswick, NJ: Rutgers University Press. 2012:110-129

66 Hanif W, Ali S, Patel K et al.Cultural competence in Covid-19 vacine rollout *BMJ* 2020;371:4845 doi:10.1136/bmj.m4845.*doi: 10.1136/bmj.m4845* [published First Online 18 Dec 2020]

67 National Institute of Healthcare (NIH). 2019;Cultural respect.www.nih.gov/institutes-nih/nih-office-director/office-communications-public-liason/clear-communication/cultural-respect (accessed Apr 2020)

68 Ahikari B, Vincent R, Wong, G. A realist review of community engagement with health research. *Wellcome Open Research* 2019; 4(87). doi: 10.12688/wellcomeopenres.15298.2 [published First Online 2 Aug 2019]

69 Fiske A, Prainsack B, Buyx A. *J Med Ethics* 2019;45:617–622. doi:10.1136/medethics-2018-105253 [published First Online 12 Jul 2019]

70 Edge D, Grey P. An Assets-Based Approach to Co-Producing a Culturally Adapted Family Intervention (CaFI) with African Caribbeans Diagnosed with Schizophrenia and Their Families. *Ethnicity & disease*, *28*(Suppl 2), 485-492.www.ethndis.org/edonline/index.php/ethndis/article/view/951 (accessed Sept 2020)

71 Nnaji C, Boone M, Pugnaire MP, et al.An Innovative Simulation-based Community-engaged Intervention for Training Research Assistants in Culturally Appropriate Informed Consent. *Prog Community Health Partnersh*. 2018;12(3):247-262. doi: 10.1353/cpr.2018.0049 [published First Online 28 Nov 2017]

72 Kadam R. Informed consent process: A step further towards making it meaningful! *Perspectives in Clinical Research*. 2017; 8(3):107-12. doi10.4103/picr.PICR_147_16 [published Sept 2017]

For peer review only - http://bmjopen.bmj.com/site/about/guidelines.xhtml

73 Grewal I and Ritchie J. Ethnic and language matching of the researcher and the research group during design, fieldwork, and analysis. In: Nazroo, J (ed) *Health and Social Research in Multiethnic Societies London:* Routledge :2006:1-17 www.taylorfrancis.com/chapters/edit/10.4324/9780203969939-12/ethnic-language-matching-researcher-research-group-design-%EF%AC%81eldwork-analysis-ini-grewal-jane-ritchie (accessed May 2020

74 Bailey Z, Feldman J, Bassett M. How Structural Racism Works — Racist Policies as a Root Cause of U.S. Racial Health Inequities. *New England Journal of Medicine* 2021;384, 768–773.doi:10.1056/nejmms2025396 [published First Online 25 Feb 2021]

75 Hardeman R, Murphy K, Karbeah J,et al. Naming Institutionalized Racism in the Public Health Literature: A Systematic Literature Review. Public Health Report . 2018;133(3): 240-249. doi: 10.1177/0033354918760574[published First Online 3 Apr 2018]

76 Moulton H, Moulton B, Lahey T, et al. Can Consent to Participate in Clinical Research Involve Shared Decision Making? *AMA J Ethics*. 2020 May 1;22(5):E365-371. www. pubmed.ncbi.nlm.nih.gov/32449651/ (accessed Dec 2021)

77 Childress J, Childress M *AMA J Ethics.*2020;22(5):E423-429. www.journalofethics.ama-assn.org/article/what-does-evolution-informed-consent-shared-decision-making-teach-us-about-authority-health-care/2020-05 (accessed Dec 2021)

78 NICE *Shared decision making* NICE guideline [NG197]. www.nice.org.uk/guidance/ng197 [published 17 Jun 2021] (accessed Jan 2022)

79 Coulter A, Collins A. Making shared decision-making a reality: no decision about me, without me *The Kings Fund* 2011. www.kingsfund.org.uk/sites/default/files/Making-shared-decision-making-a-reality-paper-Angela-Coulter-Alf-Collins-July-2011_0.pdf (accessed 5 Jan 2022)

80 Redman S, Greenhalgh T, Adedokun L et al.(2021) Co-production of knowledge: the future A new collection highlights the role of co-production in strengthening health systems *BMJ*.2021;372.doi.org/10.1136/bmj.n434 [published First Online 16 Feb 2021]

For peer review only - http://bmjopen.bmj.com/site/about/guidelines.xhtml

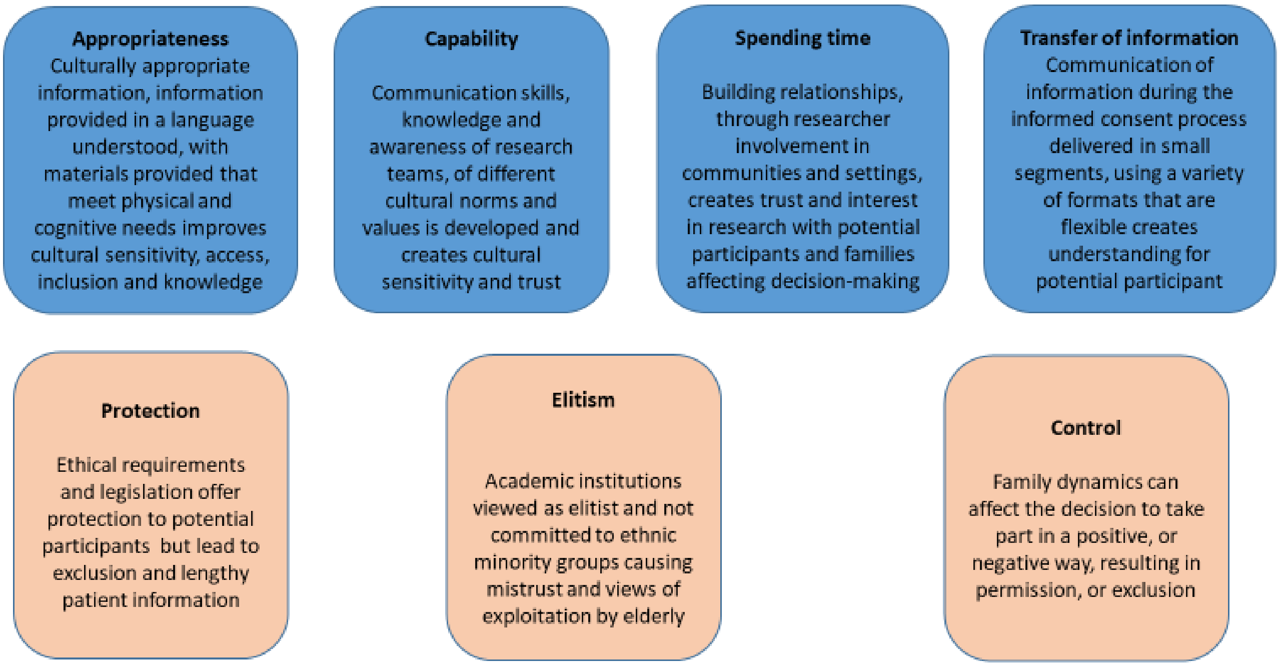

Figure 1. Theory Map –Factors influencing decisions about whether to participate in health research with under-served populations – identified from scoping search

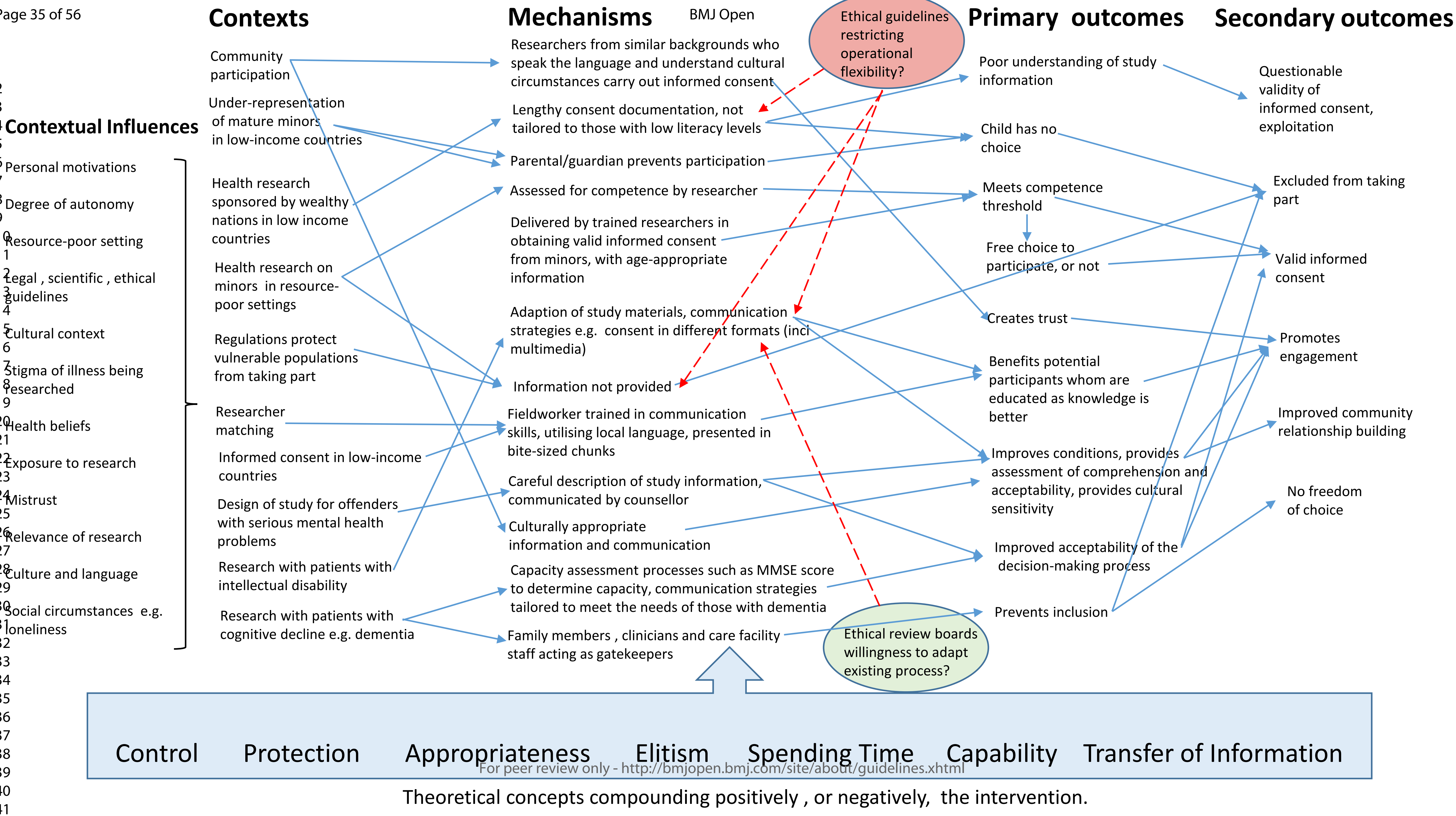

For peer review only - http://bmjopen.bmj.com/site/about/guidelines.xhtml

Flow diagram of searching

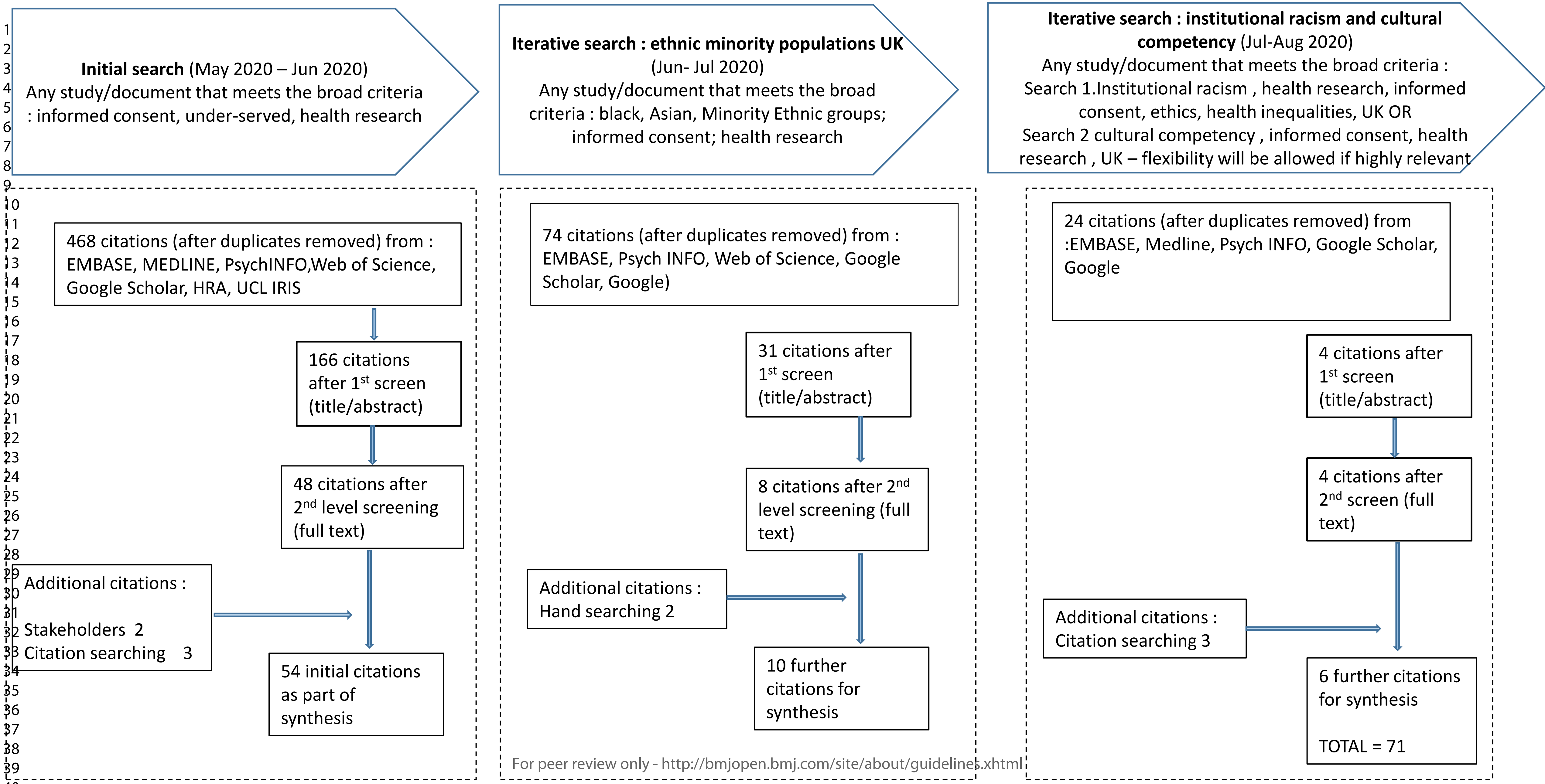

For peer review only - http://bmjopen.bmj.com/site/about/guidelines.xhtml

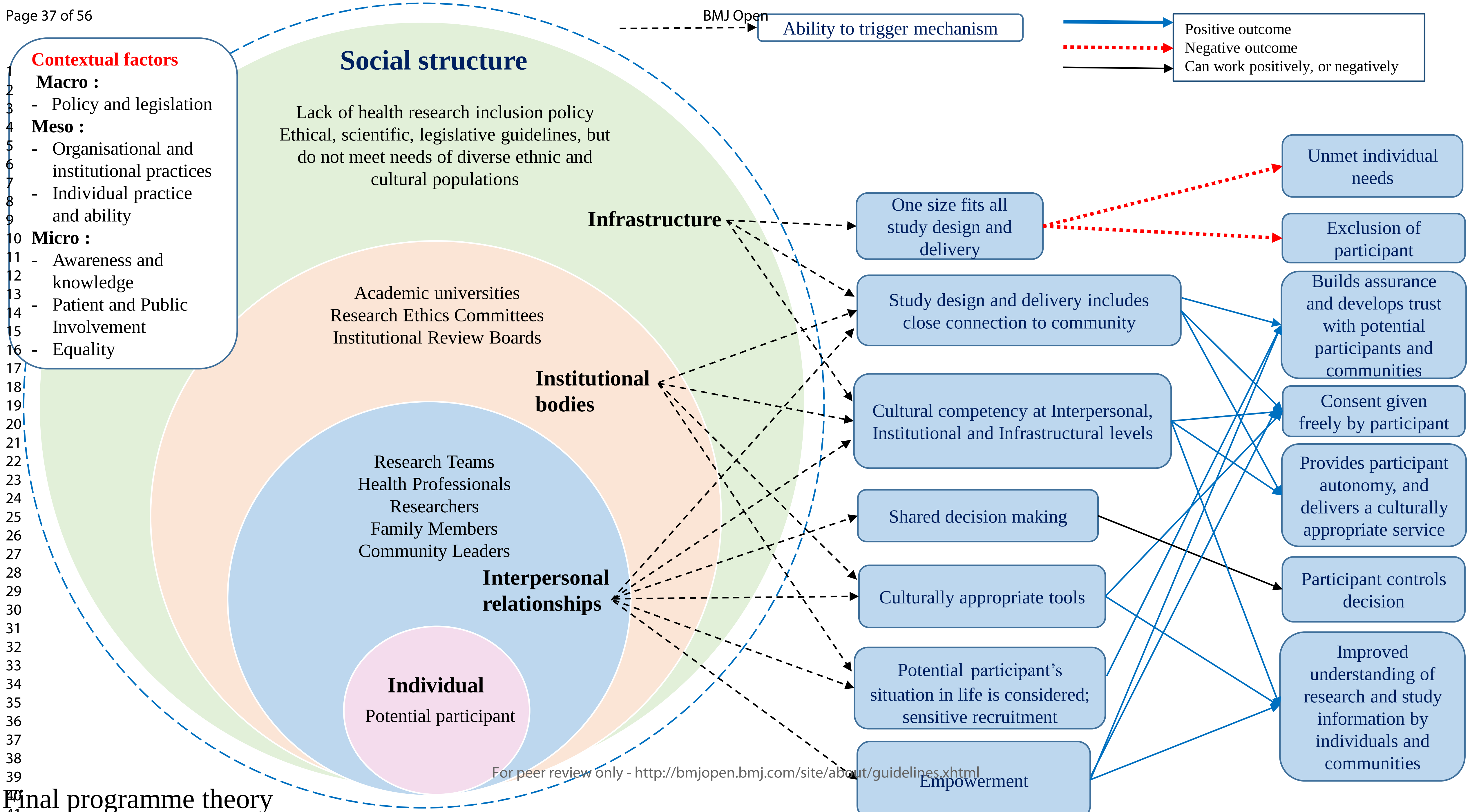

Final programme theory

Scoping Searches

Medline Scoping Search
Key terms informed consent, under-served , under-represented, health research, biomedical research 2005-current, English only

Database: Ovid MEDLINE(R) <1946 to May Week 2 2020>
Search Strategy:
----
1 informed consent.mp. or Informed Consent/ (57316)
2 exp Telemedicine/ or under-served.mp. or exp Vulnerable Populations/ (38247)
3 under-representation.mp. (756)
4 1 and 2 and 3 (0)
5 1 and 2 (820)
6 health research.mp. or exp Biomedical Research/ (277290)
7 5 and 6 (373)
8 limit 7 to (english language and yr="2005 -Current") (181)

****

Web of Science search May 21 2020

Key words : informed consent and under represented

Limits : English, 2005-current

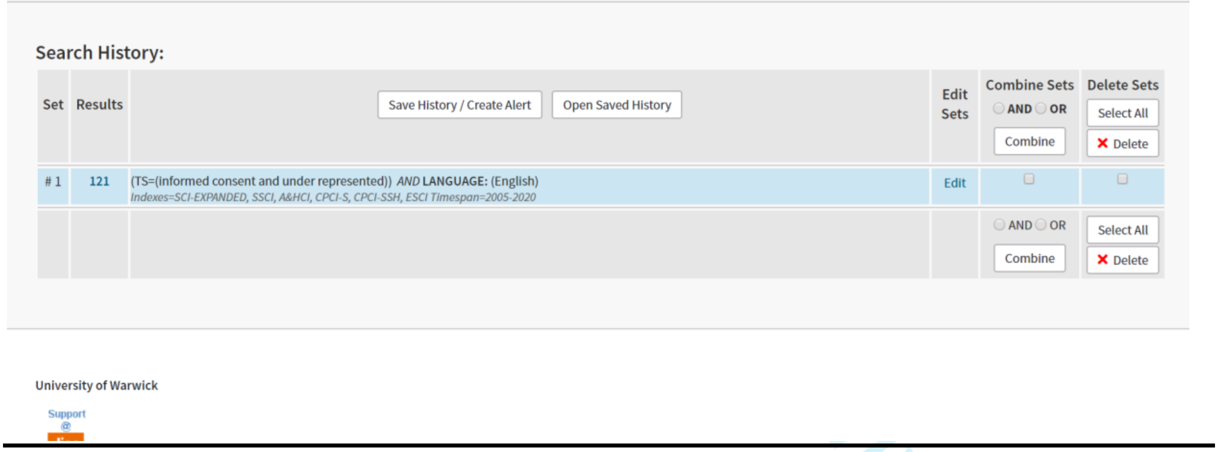

Database : UCL IRIS
Keywords : informed consent, under-served, health research, under represented

For peer review only

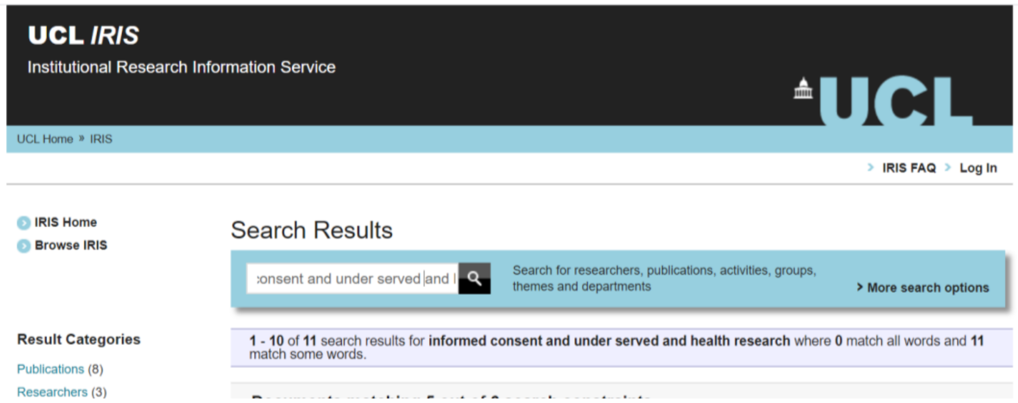

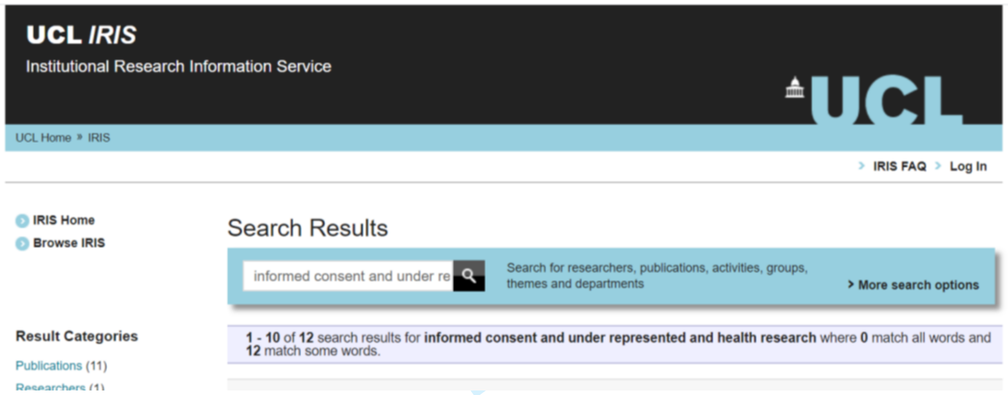

HRA (Health Research Authority)
Keywords: informed consent, underrepresented, health research

EMBASE SEARCH

Database: EMBASE <1947 to 2020 Week 20>

Search Strategy:

----

1 informed consent.mp. or exp informed consent/ (128719)

2 health research.mp. or exp medical research/ (433296)

3 1 and 2 (9419)

4 under served.mp. (587)

5 vulnerable populations.mp. or vulnerable population/ (20469)

6 under-served.mp. (587)

7 4 and 5 and 6 (27)

8 4 or 5 or 6 (21029)

9 3 and 8 (218)

10 limit 9 to english language (211)

****

Medline Search 1

Database: Ovid MEDLINE(R) <1946 to May Week 1 2020>

Search Strategy:

----

1 informed consent.mp. or exp Informed Consent/ (62033)

2 under served.mp. (312)

3 Health Services Research/ or health research.mp. or Biomedical Research/ (118031)

4 1 and 3 (2832)

5 under-served.mp. or exp Vulnerable Populations/ (10894)

6 4 and 5 (107)

****

Web of Science

TOPIC: (informed consent and under represented and health research)
Refined by: [excluding] PUBLICATION YEARS=(2004 OR 2003 OR 1993)

For peer review only

Timespan=All years. Indexes=SCI-EXPANDED, SSCI, A&HCI, CPCI-S, CPCI-SSH, ESCI.

"Title","Authors","Corporate Authors","Editors","Book Editors","Source Title","Publication Date","Publication Year","Volume","Issue","Part Number","Supplement","Special Issue","Beginning Page","Ending Page","Article Number","DOI","Conference Title","Conference Date","Total Citations","Average per Year","1900","1901","1902","1903","1904","1905","1906","1907","1908","1909","1910","1911","1912","1913","1914","1915","1916","1917","1918","1919","1920","1921","1922","1923","1924","1925","1926","1927","1928","1929","1930","1931","1932","1933","1934","1935","1936","1937","1938","1939","1940","1941","1942","1943","1944","1945","1946","1947","1948","1949","1950","1951","1952","1953","1954","1955","1956","1957","1958","1959","1960","1961","1962","1963","1964","1965","1966","1967","1968","1969","1970","1971","1972","1973","1974","1975","1976","1977","1978","1979","1980","1981","1982","1983","1984","1985","1986","1987","1988","1989","1990","1991","1992","1993","1994","1995","1996","1997","1998","1999","2000","2001","2002","2003","2004","2005","2006","2007","2008","2009","2010","2011","2012","2013","2014","2015","2016","2017","2018","2019","2020"

Database: APA PsycInfo <1806 to May Week 4 2020>

Search Strategy:

----

1 (under-served or underserved).mp. [mp=title, abstract, heading word, table of contents, key concepts, original title, tests & measures, mesh] (5394)

2 health research.mp. (5729)

3 informed consent.mp. or exp Informed Consent/ (11199)

4 biomedical research.mp. (5165)

5 (under represented or under-represented).mp. [mp=title, abstract, heading word, table of contents, key concepts, original title, tests & measures, mesh] (1120)

6 underserved.mp. (5126)

7 under served.mp. (294)

8 vulnerable population.mp. (2420)

9 6 or 7 (5394)

10 informed consent.mp. or exp Informed Consent/ (11199)

11 decision making.mp. or exp Decision Making/ (167677)

12 research.mp. or exp Experimentation/ (1223074)

13 10 or 11 or 12 (1339577)

14 9 and 13 (2287)

For peer review only

15 1 or 5 or 6 or 7 (6498)

16 2 and 3 (168)

Iteration 1- search

Database: APA PsycInfo <1806 to May Week 3 2020>

Search Strategy:

----

1 INFORMED CONSENT.mp. or exp Informed Consent/ (11049)

2 HEALTH RESEARCH.mp. (5578)

3 exp Blacks/ or exp At Risk Populations/ or UNDER-SERVED.mp. or exp Disadvantaged/ (94625)

4 1 and 2 and 3 (7)

Database: Embase Classic+Embase <1947 to 2020 Week 20>

Search Strategy:

----

1 informed consent.mp. or informed consent/ (131165)

2 health research.mp. or medical research/ (234000)

3 Black.mp. or Black person/ (178998)

4 At risk populations.mp. (3605)

5 Under-served.mp. (601)

6 disadvantaged.mp. (15612)

7 1 and 2 and 3 (20)

Web of Science 17	TOPIC: (informed consent and minority ethnic groups and health research)<br>**Refined by:** [excluding] **PUBLICATION YEARS:** (2004 OR 2002)<br>*Indexes=SCI-EXPANDED, SSCI, A&HCI, CPCI-S, CPCI-SSH, ESCI Timespan=All years*

Database: Ovid MEDLINE(R) <1946 to June Week 1 2020>

Search Strategy:

For peer review only

----

1 informed consent.mp. or Informed Consent/ (57978)

2 health research.mp. (12581)

3 Black.mp. (102740)

4 at risk populations.mp. (2115)

5 under-served.mp. or Vulnerable Populations/ (10932)

6 disadvantaged.mp. or Vulnerable Populations/ (20499)

7 1 and 2 and 3 (1)

8 2 and 5 (101)

9 1 and 8 (22)

****

For peer review only

Iteration 2 – Searches

(Search 1)

Database: Embase Classic+Embase <1947 to 2020 Week 35>

Search Strategy:

----

1 institutional racism.mp. (185)

2 health research.mp. or medical research/ (234000)

3 informed consent.mp. or informed consent/ (131165)

4 1 and 2 (3)

5 limit 4 to yr="2005 -Current" (1)

****

Database: APA PsycInfo <1806 to July Week 1 2020>

Search Strategy:

----

1 INSTITUTIONAL RACISM.mp. (453)

2 health research.mp. (5729)

3 informed consent.mp. or exp Informed Consent/ (11199)

4 1 and 2 (2)

5 limit 2 to yr ="2005-Current" (0)

Database : Web of Science July Week 1

Results: 2

(from Web of Science Core Collection)

You searched for: **TOPIC:** (institutional racism and health research and informed consent)

Timespan: All years. **Indexes:** SCI-EXPANDED, SSCI, A&HCI, CPCI-S, CPCI-SSH, ESCI.

Database: Ovid MEDLINE(R) <1946 to July Week 1 2020>

Search Strategy:

----

1 institutional racism.mp. (115)

2 Health Services Research/ or health research.mp. or Biomedical Research/ (118339)

3 1 and 2 (9)

4 limit 3 to yr="2005 -Current" (5)

(Search 2)

Database: Embase Classic+Embase <1947 to 2020 Week 25>

Search Strategy:

----

1 cultural competency.mp. or cultural competence/ (7186)

2 health research.mp. or medical research/ (234000)

3 1 and 2 (213)

4 informed consent.mp. or informed consent/ (131165)

5 3 and 4 (9)

Database: Ovid MEDLINE(R) <1946 to July Week 1 2020>

Search Strategy:

----

1 cultural competency.mp. or Cultural Competency/ (6133)

2 Health Services Research/ or health research.mp. or Biomedical Research/ (118339)

3 1 and 2 (196)

For peer review only

4 informed consent.mp. or Informed Consent/ (58028)

5 3 and 4 (8)

Database: APA PsycInfo <1806 to July Week 1 2020>

Search Strategy:

----

1 cultural competency.mp. (2952)

2 health research.mp. (5729)

3 informed consent.mp. or exp Informed Consent/ (11199)

4 1 and 2 and 3 (1)

Results: 9

(from Web of Science Core Collection)

You searched for: **TOPIC:** (cultural competency and health research and informed consent)

Timespan: All years. **Indexes:** SCI-EXPANDED, SSCI, A&HCI, CPCI-S, CPCI-SSH, ESCI.

For peer review only

Inclusion/exclusion criteria

Inclusion criteria

- Quantitative and qualitative studies, grey literature, websites, stakeholder recommendations (e.g., reports, conference papers).
- The search will be limited to a 15-year publication period (2005-2020) due to the extent to which research infrastructure and research governance has changed over that period of time. Inclusion of review articles (published within the last 15 years) included evidence from earlier studies so that we were able to draw on earlier evidence where it was relevant. In addition to this, the evidence reviewed was sufficient to inform and develop programme theory which was the purpose of the review.
- All studies that report on experiences and decision-making involved in the informed consent process will be included.
- Any source of data that is deemed relevant by the stakeholder group.
- Literature that explores the concept and practice of informed consent.
- Only sources written in English will be included.

Exclusion criteria

- Studies or sources of data that are not relevant to the informed consent process in health research in relation to under-represented populations.

CMOC Long list

The CMOC's shaded blue are specific to people of diverse ethnic and cultural backgrounds in the UK. All other CMOC's include those from the initial programme theory.

CMOC	Context (How designed and delivered)	Mechanism (tailored, how info provided, delivered)	Outcome (decision-making)	Does it work?
**CMOC1**	**C2 Meso level Organisations and people within them should deliver services with cultural competence**	**M1 Skilled , culturally competent researchers Promotes positive and effective relationships with diverse communities . Researchers need to develop and employ sensitive skills to work with people from different cultural backgrounds**	**Meets the social, cultural, religious and linguistic needs of ethnic minority populations**	**yes**
**CMOC2**	**C1 Macro level C2 Meso level informed consent can be delivered in other formats apart from written**	**M4 Flexible processes, tailored information oral, audio-recorded study information and verbal consent**	**promotes feelings of independence;**	**yes**
**CMOC3**	**C2 Meso level communities advertising research**	**M5 Community involvement shopkeepers and mosque leaders as advocates for research creates enthusiasm**	**influences decision to take part**	**yes**

For peer review only

CMOC4	C2 Meso level appropriate matching of researcher to group being researched	M4 Flexible processes, tailored information consideration of the gender of the researcher due to religious reasons can affect engagement with discussion; matching ethnic background of researcher to ethnic background of participant	participant does not feel comfortable to discuss problems in some cases depending on gender of researcher, due to religious reasons; may prevent some participants from airing counter-cultural views	no
CMOC 5	M1 Macro level<br>M2 Meso level	M4 Flexible processes, tailored information involving family, community members, or local health professionals to support shared decision making	shared decision making	yes
CMOC4	C2 Meso level ethnic matching	M4 Flexible processes, tailored information participant has some choice in which researcher they work with	effective and positive experience for potential participant, having a bearing on decision-making about taking part	yes
CMOC5	C1 Macro level<br>C2 Meso level information provided only in English	M2 Removing/restricting choice considered institutionalised racism	excludes older ethnic minority populations and migrants	no
CMOC6	C1 Macro level<br>C2 Meso level study design restricts access	M3 Inflexible process<br>M2 Removing/Restricting choice	under-representation	no

For peer review only

		eligibility criteria excludes certain populations		
**CMOC7**	**C2 Meso level commitment to confidentiality**	**M1 Skilled, culturally competent researchers researchers qualify confidentiality before obtaining consent, confirming comprehension**	**reduces fear of being reported to authorities (if migrant pop) and prevents decision of obedience to "community leaders"**	**yes**
**CMOC8**	**C1 Macro level C3 Micro level illiteracy, knowledge of health research, legal aspects**	**M3 Inflexible process signed informed consent , lacking in assurance of confidentiality**	**feel undue pressure to participate , or refuse due to fear of a negative impact on immigration process**	**no**
**CMOC9**	**M1 Macro level M2 Meso level study design**	**M1 Skilled, culturally competent researchers awareness of local protocol, legislation and culture; consideration of potential participants current situation in life; potential participant's awareness of the study; sensitive recruitment**	**culturally appropriate instruments , successful informed consent**	**yes**

For peer review only

CMOC11	M3 Micro level<br>life situation of some populations e.g. migrant communities	M2 Removing/restricting choice<br>M3 Inflexible process<br>individual decision-making not possible due to family relationships	identification of key family/community member involved in decision making with improved	yes
CMOC12	M1 Macro level<br>M2 Meso level<br>design and delivery of study	M4 Flexible processes, tailored information<br>close connection to community - conversations are basis for informed consent , summarising information, explaining written information (this can be supported by traditional methods, witnessed consent, audio-recording, video-taping or illustrations)	consent given freely, respect for cultural differences, improves trust , is ethical and ensures dignity	yes
CMOC13	M1 Macro Level<br>M2 Meso level<br>wider health care system	M3 Inflexible processes<br>M2 Removing / restricting choice<br>policies of institutions<br>1. presumption that one size fits all<br>2. difference is acknowledged but needs are misrepresented	discriminatory outcomes (e.g. exclusion due to eligibility criteria); ignores needs of ethnically diverse populations; enforces inappropriate policy and responses by health professionals	no

For peer review only

**CMOC14**	**M1 Macro level<br>M2 Meso level<br>primary health care**	**M2 Removing/restricting choice<br>gaining access<br>institutional racism used as a framework in healthcare**	**helps to understand discrimination and disadvantages minority ethnic groups experience**	**yes**
**CMOC15**	**M1 Macro level<br>low-income countries**	**M2 Removing/restricting choice<br>parental/guardian control**	**results in exclusion from taking part**	**no**
**CMOC 16**	**M1 Macro level<br>M2 Meso level<br>mature minors (often under-represented)**	**M4 Flexible processes, tailored information assessed for competence**	**meet competence threshold, providing valid informed consent**	**yes**
**CMOC 17**	**M1 Macro level<br>health research sponsored by wealthy nations in low income countries**	**M3 Inflexible process<br>Lengthy consent documentation, not tailored to those with low literacy levels**	**Inducement , poor understanding of study information, questionable validity of informed consent , exploitation**	**no**
**CMOC 18**	**M1 Macro level<br>health research in resource-poor settings**	**M1 Skilled, culturally competent researchers delivered by trained researchers in obtaining valid consent from minors, with age-appropriate study information**	**free choice to participate**	**unclear**
**CMOC19**	**M1 Macro level<br>regulations protect vulnerable populations from taking part**	**M2 Removing /restricting choice<br>information not provided**	**removes freedom of choice**	**no**

For peer review only

CMOC 20	M2 Meso level researcher matching	M1 Skilled, culturally competent researchers researchers from similar backgrounds who speak the language , understand cultural circumstances	creates trust	unclea r
CMOC2 1	M1 Macro level informed consent in low-income countries	M1 Skilled, culturally competent researchers fieldworker trained in communication skills and utilising local language to share information, in bite-size chunks	benefits potential participants whom are educated as knowledge is better	unclea r
CMOC2 2	M2 Meso level research protocol design	M3 Inflexible process M2 Removing/restricti ng choice complex forms and communication barriers, lack of social presence in the community, lack of diversity amongst researchers	results in exclusion, experience of mistrust, feel exploited and that academic institutions are not committed to ethnic minority communities, suspicion of formal transaction	no
CMOC2 3	M2 Meso level community participation and ethical review boards willingness to adapt existing process	M4 Flexible processes, tailored information adaptation of resources, consent in different formats (incl multimedia),	creates trust, better conditions , assessment of comprehension & acceptability, cultural sensitivity promotes access, community	unclea r

For peer review only

			relationship building	
CMOC24	M2 Meso level design of study for offenders with serious mental health problems	M4 Flexible processes, tailored information careful description of study, how it is communicated and by whom, in conjunction with dialogue-type counselling techniques , for patients whom are deemed to be able to make a competent decision	reduces coercion and confusion of role as a patient as opposed to a potential participant	unclear
CMOC25	M1 Macro level ethical guidelines for informed consent	M2 Removing choice/restricting choice restrict operational flexibility	lack of cultural sensitivity and appreciation of family and cultural dynamics that affect decision-making	no
CMOC26	M1 Macro level many African countries	M1 Skilled, culturally competent researchers culturally appropriate delivery, taking into consideration social and cultural conventions, familiarity with local context of research	improved acceptability of the decision-making process	yes
CMOC27	M2 Meso level M1 Micro level	M2 Removing/restricting choice	prevents inclusion, and	no

For peer review only

	research with patients with cognitive decline	family members, clinicians and care facility staff acting as gatekeepers	freedom of choice	
CMOC28	M2 Meso Level<br>M1 Micro level<br>research with patients with cognitive decline e.g. dementia	M4 Flexible processes, tailored information capacity assessment processes such as a MMSE score, as a way of determining capacity to consent and communication strategies tailored to meet needs of those with dementia	enables person to make own decision , whilst is a continuous check of willingness	yes
CMOC29	M2 Meso level<br>M1 Micro level<br>research with people with intellectual disability	M4 Flexible processes, tailored information adapting study materials and communication strategies during the informed consent process	builds rapport and engagement with participant and enables them to participate in the consent process to the best of their ability	yes

For peer review only

PRISMA 2020 Checklist

Section and Topic	Item #	Checklist item	Location where item is reported
**TITLE**			
Title	1	Identify the report as a systematic review.	N/A (realist review) RAMESES guidelines: http://www.ramesesproject.org/
**ABSTRACT**			
Abstract	2	See the PRISMA 2020 for Abstracts checklist.	N/A
**INTRODUCTION**			
Rationale	3	Describe the rationale for the review in the context of existing knowledge.	Page 5 (lines 107-109)
Objectives	4	Provide an explicit statement of the objective(s) or question(s) the review addresses.	Page 6 (lines 133-137)<br>Page 5 (lines 121-122)
**METHODS**			
Eligibility criteria	5	Specify the inclusion and exclusion criteria for the review and how studies were grouped for the syntheses.	Page 10 (line 227-228)
Information sources	6	Specify all databases, registers, websites, organisations, reference lists and other sources searched or consulted to identify studies. Specify the date when each source was last searched or consulted.	Page 9 (lines 203-205)
Search strategy	7	Present the full search strategies for all databases, registers and websites, including any filters and limits used.	Page 9 (lines 202-206,218-219)
Selection process	8	Specify the methods used to decide whether a study met the inclusion criteria of the review, including how many reviewers screened each record and each report retrieved, whether they worked independently, and if applicable, details of automation tools used in the process.	Page 9 (lines 207-214)<br>Page 10 (lines 227-230)
Data collection process	9	Specify the methods used to collect data from reports, including how many reviewers collected data from each report, whether they worked independently, any processes for obtaining or confirming data from study investigators, and if applicable, details of automation tools used in the process.	Pages 10-11 (lines 229-245)
Data items	10a	List and define all outcomes for which data were sought. Specify whether all results that were compatible with each outcome domain in each study were sought (e.g. for all measures, time points, analyses), and if not, the methods used to decide which results to collect.	N/A
	10b	List and define all other variables for which data were sought (e.g. participant and intervention characteristics, funding sources). Describe any assumptions made about any missing or unclear information.	N/A
Study risk of bias assessment	11	Specify the methods used to assess risk of bias in the included studies, including details of the tool(s) used, how many reviewers assessed each study and whether they worked independently, and if applicable, details of automation tools used in the process.	N/A
Effect measures	12	Specify for each outcome the effect measure(s) (e.g. risk ratio, mean difference) used in the synthesis or presentation of results.	N/A
Synthesis methods	13a	Describe the processes used to decide which studies were eligible for each synthesis (e.g. tabulating the study intervention characteristics and comparing against the planned groups for each synthesis (item #5)).	N/A

For peer review only

PRISMA 2020 Checklist

Section and Topic	Item #	Checklist item	Location where item is reported
	13b	Describe any methods required to prepare the data for presentation or synthesis, such as handling of missing summary statistics, or data conversions.	N/A
	13c	Describe any methods used to tabulate or visually display results of individual studies and syntheses.	N/A
	13d	Describe any methods used to synthesize results and provide a rationale for the choice(s). If meta-analysis was performed, describe the model(s), method(s) to identify the presence and extent of statistical heterogeneity, and software package(s) used.	N/A
	13e	Describe any methods used to explore possible causes of heterogeneity among study results (e.g. subgroup analysis, meta-regression).	N/A
	13f	Describe any sensitivity analyses conducted to assess robustness of the synthesized results.	N/A
Reporting bias assessment	14	Describe any methods used to assess risk of bias due to missing results in a synthesis (arising from reporting biases).	N/A
Certainty assessment	15	Describe any methods used to assess certainty (or confidence) in the body of evidence for an outcome.	N/A
**RESULTS**			
Study selection	16a	Describe the results of the search and selection process, from the number of records identified in the search to the number of studies included in the review, ideally using a flow diagram.	Page 9 (line 219 (figure 3))
	16b	Cite studies that might appear to meet the inclusion criteria, but which were excluded, and explain why they were excluded.	N/A
Study characteristics	17	Cite each included study and present its characteristics.	N/A
Risk of bias in studies	18	Present assessments of risk of bias for each included study.	N/A
Results of individual studies	19	For all outcomes, present, for each study: (a) summary statistics for each group (where appropriate) and (b) an effect estimate and its precision (e.g. confidence/credible interval), ideally using structured tables or plots.	N/A
Results of syntheses	20a	For each synthesis, briefly summarise the characteristics and risk of bias among contributing studies.	N/A
	20b	Present results of all statistical syntheses conducted. If meta-analysis was done, present for each the summary estimate and its precision (e.g. confidence/credible interval) and measures of statistical heterogeneity. If comparing groups, describe the direction of the effect.	N/A
	20c	Present results of all investigations of possible causes of heterogeneity among study results.	N/A
	20d	Present results of all sensitivity analyses conducted to assess the robustness of the synthesized results.	N/A

For peer review only

PRISMA 2020 Checklist

Section and Topic	Item #	Checklist item	Location where item is reported
Reporting biases	21	Present assessments of risk of bias due to missing results (arising from reporting biases) for each synthesis assessed.	Page 22 (lines 492-494)
Certainty of evidence	22	Present assessments of certainty (or confidence) in the body of evidence for each outcome assessed.	N/A
**DISCUSSION**			
Discussion	23a	Provide a general interpretation of the results in the context of other evidence.	Page 19 (lines 420-425)
	23b	Discuss any limitations of the evidence included in the review.	N/A
	23c	Discuss any limitations of the review processes used.	Page 22 (lines 488-494)
	23d	Discuss implications of the results for practice, policy, and future research.	Page 17-19 (lines 416-417 (Table 1))
**OTHER INFORMATION**			
Registration and protocol	24a	Provide registration information for the review, including register name and registration number, or state that the review was not registered.	Not registered
	24b	Indicate where the review protocol can be accessed, or state that a protocol was not prepared.	https://systematicreviewsjournal.biomedcentral.com/articles/10.1186/s13643-021-01652-2
	24c	Describe and explain any amendments to information provided at registration or in the protocol.	N/A
Support	25	Describe sources of financial or non-financial support for the review, and the role of the funders or sponsors in the review.	Page 24 (lines 547-554)
Competing interests	26	Declare any competing interests of review authors.	Page 24 (line 557)
Availability of data, code and other materials	27	Report which of the following are publicly available and where they can be found: template data collection forms; data extracted from included studies; data used for all analyses; analytic code; any other materials used in the review.	Page 7 Supplementary file 1 (lines 168-169)<br>Page 9 Supplementary file 2 (lines 203)<br>Page 10 Supplementary file 3 (line 227)<br>Page 13 Supplementary file 4 (line 298)

From: Page MJ, McKenzie JE, Bossuyt PM, Boutron I, Hoffmann TC, Mulrow CD, et al. The PRISMA 2020 statement: an updated guideline for reporting systematic reviews. BMJ 2021;372:n71. doi: 10.1136/bmj.n71

For more information, visit: http://www.prisma-statement.org/

For peer review only

BMJ Open

Factors influencing decisions about whether to participate in health research by people of diverse ethnic and cultural backgrounds: a realist review.

Journal:	*BMJ Open*
Manuscript ID	bmjopen-2021-058380.R2
Article Type:	Original research
Date Submitted by the Author:	08-Mar-2022
Complete List of Authors:	Hoverd, Eleanor; University of Warwick<br>Hawker-Bond, George; Oxford University, Nuffield Department of Clinical Neurosciences,<br>Staniszewska, Sophie; University of Warwick, Warwick Medical School<br>Dale, Jeremy; University of Warwick, Warwick Medical School
**Primary Subject Heading**:	Health services research
Secondary Subject Heading:	Ethics
Keywords:	Health policy < HEALTH SERVICES ADMINISTRATION & MANAGEMENT, MEDICAL ETHICS, Clinical trials < THERAPEUTICS

SCHOLARONE™
Manuscripts

I, the Submitting Author has the right to grant and does grant on behalf of all authors of the Work (as defined in the below author licence), an exclusive licence and/or a non-exclusive licence for contributions from authors who are: i) UK Crown employees; ii) where BMJ has agreed a CC-BY licence shall apply, and/or iii) in accordance with the terms applicable for US Federal Government officers or employees acting as part of their official duties; on a worldwide, perpetual, irrevocable, royalty-free basis to BMJ Publishing Group Ltd ("BMJ") its licensees and where the relevant Journal is co-owned by BMJ to the co-owners of the Journal, to publish the Work in this journal and any other BMJ products and to exploit all rights, as set out in our licence.

The Submitting Author accepts and understands that any supply made under these terms is made by BMJ to the Submitting Author unless you are acting as an employee on behalf of your employer or a postgraduate student of an affiliated institution which is paying any applicable article publishing charge ("APC") for Open Access articles. Where the Submitting Author wishes to make the Work available on an Open Access basis (and intends to pay the relevant APC), the terms of reuse of such Open Access shall be governed by a Creative Commons licence – details of these licences and which Creative Commons licence will apply to this Work are set out in our licence referred to above.

Other than as permitted in any relevant BMJ Author's Self Archiving Policies, I confirm this Work has not been accepted for publication elsewhere, is not being considered for publication elsewhere and does not duplicate material already published. I confirm all authors consent to publication of this Work and authorise the granting of this licence.

For peer review only

TITLE

Factors influencing decisions about whether to participate in health research by people of diverse ethnic and cultural backgrounds: a realist review.

Corresponding Author: Mrs. Eleanor Hoverd, NIHR Clinical Research Network West Midlands, Room A117, Warwick Medical School, Warwick University, Gibbet Hill Rd, Coventry CV4 7AL, England eleanor.hoverd@nihr.ac.uk

Co-authors:

Dr. George Hawker-Bond, MChem (Hons) Chemistry, MBChB in Medicine and Surgery, Nuffield Department of Clinical Neurosciences, University of Oxford, Oxford, England george.hawker-bond@medsci.ox.ac.uk

Professor Sophie Staniszewska, DPhil (Oxon), BSc (Hons), Warwick Research in Nursing, Warwick Medical School, Warwick University, Coventry, England Sophie.Staniszewska@warwick.ac.uk

Professor Jeremy Dale, Unit of Academic Primary Care, Warwick Medical School, Warwick University, Coventry, England jeremy.dale@warwick.ac.uk

Word count 4806

For peer review only - http://bmjopen.bmj.com/site/about/guidelines.xhtml

ABSTRACT

Objective To develop and refine a programme theory that explains factors that influence decisions to take part in health research by people of diverse ethnic and cultural backgrounds.

Design Realist review following a sequence of five steps: 1) scoping search and identification of programme theory 2) evidence searching 3) critical appraisal and data extraction 4) organisation of evidence 5) refinement of programme theory.

Eligibility criteria Documents (including peer-reviewed articles, grey literature, websites, reports, and conference papers) either full text, or a section of relevance to the over-arching research question were included.

Data sources EMBASE, Medline, Web of Science, Psych Info, Google and Google Scholar were searched iteratively between May through August 2020. Search strategy was refined for each database providing a broad enough review for building of programme theory.

Analysis Data from eligible documents was extracted to build understanding of the factors that influence decision-making. Data was mapped to create a data matrix according to Context (C), Mechanism (M), Outcome (O), Configurations (C) (CMOCs) for the process of informed consent, to aid interpretation and produce final programme theory.

Results 566 documents were screened and 71 included. Final programme theory was underpinned by CMOCs on processes influencing decisions to take part in research. Key findings indicate the type of infrastructure required, e.g., resources, services and policies, to support inclusion in health research, with a greater need to increase the social presence of researchers within communities, improve cultural competency of individuals and organisations, reduce the complexity of participant information, and provide additional resources to support adaptive processes and shared decision making.

Conclusion The review indicates the need for a more inclusive research infrastructure that facilitates diverse participation in health research through incorporating adaptive processes

For peer review only - http://bmjopen.bmj.com/site/about/guidelines.xhtml

that support shared decision making within the informed consent process and in the conduct of research projects.

For peer review only - http://bmjopen.bmj.com/site/about/guidelines.xhtml

ARTICLE SUMMARY

Strengths and limitations of this study

·Using realist methods to explore the contexts and mechanisms of the complexities affecting the informed consent process in health research, for people of diverse ethnic and cultural populations, allowed new insight to emerge.

·There is a paucity of evidence around the informed consent process in health research with people of diverse ethnic and cultural backgrounds, limiting the evidence from which to extract data from.

·Relevance and rigour were increased through the involvement of National Institute for Health Research (NIHR) (Public) Research Champions, healthcare professionals and clinical academics in refining the programme theory.

·Owing to the COVID-19 pandemic, stakeholder involvement in the programme was conducted remotely, enabling access from a wider geographical spread of stakeholders.

·Whilst the review was undertaken in the context of the UK health care system, the programme theory is likely to have wider applicability.

Keywords: realist review, informed consent, under-served, ethnic minority, health research

For peer review only - http://bmjopen.bmj.com/site/about/guidelines.xhtml

INTRODUCTION

People who have poorer health outcomes, and have greater needs are currently under-served by the way in which health research is accessed and delivered.[1] Lack of diversity of participants taking part in health research, poses a serious challenge both within the UK and globally, around how to address and develop a more inclusive health research system so that under-served populations can benefit. It has been argued that the informed consent process may be contributing to inequalities in health research through a lack of flexibility in the process.[2-10] The informed consent process in health research aims to provide patients and the public with the information they require to make a voluntary decision about participation in health research. In May 2019, the International Council for Harmonisation (ICH)[11] drafted the *General Considerations for Clinical Studies E8 (R1)* suggesting that a "*one size fits all approach*" to studies should be avoided. Hence, it is important to unravel the factors that may affect the decision to take part in health research by these populations, to develop a system that is more inclusive.

Evidence suggests that the informed consent process, including the language used within it, creates a serious barrier to access and participation.[8,12-17] Several systematic reviews highlight common barriers to the informed consent process and indicate the need for further research to determine how information is delivered and what information people need, to make an informed decision about participation in health research.[18-20] There is less focus in the literature around what works, to inform research design and delivery. It is a critical issue that requires considerable improvement to develop a more inclusive health research system.

Conducting a realist review of the informed consent process in health research with under-served populations, is a well-suited method for exploring such a complex intervention[21] to explain how the informed consent process may work for whom, how and under what

For peer review only - http://bmjopen.bmj.com/site/about/guidelines.xhtml

circumstances. The over-arching research question for this review is: What contextual factors influence the decision to take part in health research in under-served populations?

The term under-served is preferred when referring to groups or communities who are under-represented in health research e.g., marginalised groups, and is also used to describe geographical locations such as rural or low-income countries, although a single definition is not found within the literature.[1] There are a number of intersecting factors that contribute to groups being under-served; such as demographic, socioeconomic, health status and disease-specific status, leading to disadvantage and discrimination.[1] However, being under-served is also likely to be context-specific, with key characteristics related to trial design influencing the ability to participate.[1] For example, the informed consent process is recognised as a specific barrier to inclusion in health research by under-served populations.[1]

Objectives were as follows:

1. To undertake a realist review to identify the contexts and mechanisms that affect the informed consent process and the decision to participate in health research in under-served populations .
2. To draw on the review's programme theory to develop guidance that addresses barriers to participating in health research in under-served populations.

METHODS

In this study, we undertook a realist review to produce a programme theory to explain the informed consent process, and hence provide actionable recommendations for policymakers and health researchers to inform the development of inclusive health research. The review process followed the steps as laid out in the protocol[22] and was conducted and reported in accordance with RAMESES (Realist and Meta-narrative Evidence Syntheses : Evolving Standards) guidance and publication standards, as well as providing a rationale for selecting

For peer review only

this approach.[23] RAMESES guidance has been produced to provide initial reporting standards for realist reviews and evaluations.[23]

Following a realist analytical process, Context, Mechanism, Outcome, Configurations (CMOCs) were built by EH. These were shared and discussed at meetings with the review team and stakeholders whereby feedback was encouraged which contributed to further refinement and establishing a long list of CMOCs. The CMOCs conceptualise key features that support explanation and understanding of complex programmes and create a model that describes how mechanisms are activated, for who and under what circumstances, which affects outcomes.[23] Summaries were developed after each meeting to support reflection and understanding with stakeholders. Diagrams were constructed and refined to support explanation of the findings.

Patient Public Involvement

The National Institute for Health Research (NIHR) funds health and social care research in the UK and advocates for patient and public involvement (PPI) throughout all stages of research. To support PPI, the NIHR have introduced the role of NIHR (Public) Research Champions who are patients or members of the public, and volunteer to raise awareness of health and social care research as well as help researchers to understand the participant experience.[24] Six NIHR (Public) Research Champions were involved in this review, helping to clarify the scope of the review, contributing to the development of programme theory and developing recommendations. Three of the NIHR (Public) Research Champions were from populations of diverse ethnic and cultural backgrounds.[24] Their involvement is discussed in more detail, below (step 1).

Development of an initial programme theory (step 1)

In order to develop an initial programme theory, a scoping search (see online supplementary file 1 Scoping searches) was carried out based on search terms centred on the intervention

For peer review only - http://bmjopen.bmj.com/site/about/guidelines.xhtml

under study (informed consent) with under-served populations in health research, to explore key literature in this field. This allowed us to explore the available literature and to create some initial boundaries.[25] Theoretical concepts and evidence that would contribute to explaining different outcomes; for example, positive (understood study information) or negative experiences (excluded from taking part). A theoretical concept map was created (figure 1) identifying higher-level, abstract theories which provided a basis to discuss some of the larger complexities of informed consent with the stakeholder group in combination with the empirical evidence from the scoping search, as a way of supporting, contradicting , or modifying the programme theory and to refine the purpose of the review.[25]

The initial scoping search resulted in 16 documents selected based on relevance to the over-arching research question.[4,26-37] These were examined, with codes applied to label CMOCs, concepts and theories, which were helpful in building the initial programme theory. These CMOCs were then collated, a long list of CMOCs drawn-up and then developed into a diagram, to create a visualisation of the initial programme theory (figure 2). This enabled identification of key theories for further exploration.

Stakeholder involvement

Stakeholders included NIHR (Public) Research Champions, health professionals (research nurses, a dietician, physiotherapists and a general practitioner) and clinical academics who were openly invited to participate via the review team's associated Medical School and NIHR Clinical Research Network (CRN). A total of six meetings were held virtually (three with NIHR (Public) Research Champions and three with the mixed group of health professionals and clinical academics). Stakeholders were geographically widespread and thus virtual meetings enabled easier access than had meetings been held face to face. At each stakeholder meeting, background information was provided, including an explanation of realist methodology. The facilitated discussions covered under-served populations; initial

For peer review only - http://bmjopen.bmj.com/site/about/guidelines.xhtml

programme theory; analysis; results and recommendations; and stakeholder feedback and advice.

Meetings were organised to provide perspectives and expert opinions around issues relevant to building programme theory, insights into the ways in which social structure may influence the informed consent process, as well as identifying any pertinent documents of relevance.[21] Stakeholder input is described in the protocol.[22]

Evidence searching (step 2)

Literature searches were conducted iteratively and conducted between May through August 2020 (see online supplementary file 2 Searches) . The initial search used the databases EMBASE, Medline, Web of Science and Psych Info, in addition to manual-searching using Google and Google Scholar. The search strategy was refined for each database , aiming to provide a broad enough review for further building of programme theory.

Following the initial search, 468 citations were identified following removal of duplicates, with 54 citations remaining following 1st and 2nd level screening by EH and G H-B. As synthesis progressed, key theories were emerging around the informed consent process with people of diverse ethnic and cultural backgrounds, with most of literature from the US. Therefore, following discussion with stakeholders and the review team, it was agreed synthesis would focus on a smaller number of key theories with additional searches required to test and refine programme theory, focussing on a "*conceptually rich*"[38] sub-group of literature to produce recommendations relevant to the UK health research system. The reason for limiting the subsequent searches to test theories within the UK literature was because an inclusion policy is lacking, and with a free , publicly funded health care system, context and resources may differ in comparison to other geographical locations, thus affecting mechanisms and outcomes. A flow diagram of the iterative searching process is displayed in figure 3, based on RAMESES guidelines.[23]

For peer review only - http://bmjopen.bmj.com/site/about/guidelines.xhtml

A key consideration of a realist review is to reflect on contextual factors of the social system(s) and how this affects the working of the intervention.[21] In developing the initial programme theory, we sought to view the informed consent process through the larger social system at micro (individual), meso (organisational, institutional) and macro (policy) levels, where interactions between individuals and levels can be understood better.[39] This was aimed at bringing about recommendations for change.[20]

Selection and appraisal of documents (step 3)

Broad inclusion and exclusion criteria (see online supplementary file 3 Inclusion/Exclusion criteria) were applied in the selection and appraisal process, each document was assessed for relevance and rigour by EH and G H-B, independently.[40] Relevance and rigour were assessed in parallel. Decisions were based on whether a document was relevant to the over-arching research question and what it contributed to the review. Rigour was determined by assessing methodological rigour and whether a document included important information that may help with interpretation, as well as providing sufficient detail to determine trustworthiness.[23] Quality of data was noted in data extraction forms and considered during analysis and synthesis. All relevant data was used to build the arguments that supported the final programme theory, including systematic and literature reviews.[25] Two papers included in the review are noted to be included in a systematic review, but do not impact upon the review in terms of the importance given to CMOC development.[6,41] Documents identified as being helpful in developing, refuting and refining the programme theory were sought, although the number of iterations and length of time spent on this was limited due to the timescale of the review.[25]

EH screened all documents identified through searches, by title and abstract. Second reviewer G H-B screened a random 10% of the documents.[42] To establish inter-reviewer agreement,

For peer review only

a kappa measure of k>0.8 was pre-determined, with a resultant kappa measure of 0.90 following second level screening by EH and G H-B .

Data analysis and synthesis (step 4)

A combination of annotations and notes, along with a data extraction form was used to classify information from documents about potential candidate theories, through noting CMOC concepts.[20] A data extraction form was adapted from Rycroft-Malone[43] to record characteristics of documents and whether evidence was good enough and relevant to include in the synthesis. This included author, year, source, issues of sample size, data collection and data analysis, and interpretations made, characteristics of theoretical concepts and the impact these characteristics may have on the informed consent process (e.g., characteristics of appropriateness of informed consent and how these characteristics may impact on the decision to take part in research). The data extraction form was tested on the first three documents to confirm its applicability and usefulness.

Refinement and validation of programme theory (step 5)

In step 5, both data and annotations were combined. Data synthesis was undertaken by EH through mapping data to create a data matrix according to CMOCs which helped with interpretations and in developing theory.[23] Contexts were themed according to the level of the social system within which they related to.[39] Through noting common features, or relationships and identifying any underlying concepts, recurring themes were identified and patterns in the evidence were tested.

Recurring contexts and outcomes were explained through mechanisms. For example, within the included texts, the design of some research studies were reported as resulting in exclusion of people of diverse ethnic and cultural backgrounds, leading to mistrust, feelings of being exploited and a belief that academic communities are not committed to understanding the needs and experiences of people from diverse ethnic and cultural communities.[5,37,44-46]

For peer review only - http://bmjopen.bmj.com/site/about/guidelines.xhtml

During data synthesis the aim was to provide an explanation of these contexts and outcomes through the identification of mechanisms. Further studies were sought to test the various elements of programme theory. Synthesis results were shared and discussed at stakeholders' meetings, to ensure validity and consistency of the interpretations made. The extraction of data, analysis and synthesis process was iterative with documents repeatedly read at various points throughout the process.

An overview of theories is described in the results section, through a narrative of how the informed consent process works, for whom, how and under what circumstances based upon the evidence in this review. References are made to CMOCs , including some quotations to draw out key aspects of CMOCs.

Development of recommendations (step 6)

Recommendations were developed with stakeholders through presenting the findings and a narrative of the programme theory through a virtual meeting. EH led the remote discussion leading to agreement upon a set of practical recommendations aimed at government agencies, Research Ethics Committees (RECs), researchers, and health professionals. Input from stakeholders was intended to ensure their practicality.

RESULTS

Document characteristics

In total, seventy-one documents published between 2005 and 2020 were included from 19 countries with the majority from the USA (37%), UK (24%) and Africa (13%). The date range of the sources was from 2004 to 2020. The source type was mixed including discussion papers and opinion pieces (17, 24%), qualitative research (27, 38%), literature reviews (4, 6%) , mixed methods research (2, 3%), systematic reviews (5, 8%), quantitative research (9, 13%), guidelines (2, 3%), case study (1, 1%), book chapter (1, 1%), audit (1, 1%), report (1, 1%) and a working paper (1, 1%).

Context Mechanism Outcome Configurations (CMOCs)

The key themes that emerged related to inter-relationships; organisational and individual cultural competency and adaptive processes. Patterns in Mechanisms were identified as can be seen on the long list of 29 CMOCs used to develop programme theory (see online supplementary file 4 CMOC Long List). These are discussed below making reference to the associated, numbered CMOCs and selected quotations from documents which help to explain the theories.

Inter-relationships

Developing trusting relationships between researchers and under-served communities is important. Key family members, community, or faith leaders and local healthcare staff may influence a potential participant's decision to take part (CMOC 3, CMOC 5, CMOC 11).[3,47,48] The approach to identifying key individuals must be done sensitively with clear information provided about what participation involves (CMOC 1, CMOC 7, CMOC 9, CMOC 18, CMOC 26).[3,6,17-49] This can lead to increased autonomy, trust and a positive view towards health research with the key individuals becoming a trusted point of information, thus helping to improve knowledge about health research, increasing the awareness and confidence of communities to participate in research.[3]

> *…research assistants attempted to immerse themselves in the activities of the diabetes clinic…*[6] (P.7) (CMOC 1).

Researcher-matching may help (CMOC 1, CMOC 4) as it provides some choice for the potential participant as to whom they work with[7,50] and can enable more effective and positive experiences. This has been used in trials on diabetes, mental health, and cardiovascular disease with participants of South Asian heritage and Black British, African, and African-Caribbean heritage.[51,52] There does not appear to be one definition of researcher-matching; examples include: an interviewer speaking the same language as the

For peer review only - http://bmjopen.bmj.com/site/about/guidelines.xhtml

participant conducts interviews; a health professional who speaks the same language as participant conducts intervention; an interpreter is present to support delivery of the intervention; trained multi-lingual facilitators; ethnically matched outreach team delivers the intervention; research assistants who speak the same language; and culturally adapted interventions.[2,9,17,37,52-59] The cost implications of this approach were rarely reported, with only one study discussing the impact and challenges of training facilitators and the complexities of translating material.[9] The perceptions of having interviews or focus groups conducted by someone of the same ethnicity is under-explored and viewed by some researchers as not as essential as language, gender, technical and interpersonal skills which may be more important.[9] There was some suggestion that researcher-matching should be used with caution as there is a risk that this may lead to researchers lacking cultural competence.[60] There is also a risk when using interpreters who have been trained to facilitate qualitative interviews that the richness of data could be compromised.[61]

Family relationships may trigger a positive or negative experience of the decision-making process (CMOC 5, CMOC 11).[2-4,62] Identification of key family /community members involved in decision-making process can determine whether someone takes part and conversely can improve the choice through improved understanding (CMOC 5).[2-3,60,62]

Organisational and individual cultural competency

There is considerable evidence to support the importance of delivering services with cultural competence (CMOC 2, CMOC 9).[3,5] Cultural competence is defined as the way in which behaviours, attitudes, and policies of a system, or amongst individuals, such as health care professionals, enables a service to operate effectively amongst diverse cultural situations.[63] Developing cultural competency and leadership helps to develop a capable, skilled research workforce (CMOC 1)[3,5,7,10] protecting the autonomy of potential participants,

For peer review only - http://bmjopen.bmj.com/site/about/guidelines.xhtml

encouraging reflection on the individual's life situation, and developing awareness of different cultures.[5]

Contextual factors such as the life situation of some populations (e.g., migrant communities) can influence the decision-making process in health research due to

> *...fear of identification and reporting to authorities*[5](P.13) (CMOC 11).

Whilst cultural competence is evidenced to improve the responsiveness and appropriateness in the way in which health research is delivered, as well as reducing health disparities, it requires further exploration that goes beyond the scope of this review.[3,5,7,10,17] There is some evidence to suggest that cultural competency conflates language, geographical origin, ethnicity and race, potentially diluting the identity of individuals and communities.[64] The term has caused considerable debate since the introduction of the concept in the 1980's[63] with some asserting that it is not possible to be competent in other individuals' cultures.[59,65] However, cultural competence has been considered vital for engaging with diverse ethnic and cultural communities to ensure accurate information is shared appropriately to instil confidence in the safety and efficacy of COVID-19 vaccines.[66] Cultural competency when positioned within a human rights framework is viewed positively as a way of operationalising respect, providing a service that is culturally appropriate.[64,67]

Adaptive processes

Providing small bitesize participant information about an available health research study, in local languages, with local cultural context supports understanding.[5,17,68] In addition to this, co-production and more community-based participatory approaches may encourage wider participation, offering a way to empower individuals and communities who are under-served in health research.[69] It has been demonstrated that engagement with people of African-Caribbean heritage in the development of a culturally-adapted family intervention for people with schizophrenia can be achieved by taking a two-way approach, whereby

For peer review only - http://bmjopen.bmj.com/site/about/guidelines.xhtml

researchers become integrated within communities and individuals from communities are able to access training and support around health research to increase capability as part of a community based participatory research (CBPR) approach, this has been demonstrated.[70] Taking these approaches improves accountability and makes provided information more relevant.

> *…members of target populations should be involved in the overall design of research…*[41] (P.5) (CMOC 9, CMOC 14).

In addition, it may be helpful to offer a range of materials (e.g., written, oral, audio, and multi-media) to ensure concepts are understood (CMOC 2, CMOC 12).[3,28] Providing study information in various formats can promote feelings of independence, leading to consent being given freely, serving more respect for cultural differences (CMOC 2, CMOC 14).[6,7,71]

> *...this way you won't need to depend on your children anymore* [6](P6*)* (CMOC 3).

Some groups lack an agreed written form of their main language, and alternative formats are useful in this respect (CMOC 2, CMOC 12).[6-7]

This creates trust and ensures dignity.[3,5-6,10,17,51]

Institutional barriers

When the needs (e.g., cultural, linguistic or historical) of people from diverse ethnic and cultural backgrounds are misrepresented or ignored [3,5,72] (CMOC 6, CMOC 8, CMOC 13) this results in: discriminatory outcomes (e.g., exclusion due to eligibility criteria) and enforces inappropriate ethical, legislative, scientific guidelines and recruitment processes (CMOC 5, CMOC 13).[3,5,8] It has been recognised that individuals from diverse ethnic and cultural backgrounds are under-represented in commissioning and making funding decisions about health research, and on editorial boards of journals (CMOC 13).[9]

For peer review only - http://bmjopen.bmj.com/site/about/guidelines.xhtml

Study designs can restrict access to health research through creating eligibility criteria that excludes populations from taking part, usually due to a language barrier.[3]

> *...many studies require participants to have the ability to understand and speak English which excludes older people from ethnic minority communities and/or migrants*[3] (P.6) (CMOC 5, CMOC 9, CMOC 12, CMOC 13).[3,72-73]

This results in under-representation and may be perceived as institutional racism (CMOC 5, CMOC 9, CMOC 12, CMOC 13).[2,8,58,73]

> *…dearth of research with ethnic minority communities in the UK is a reflection of the lack of influential policy…*[3](P.9) (CMOC 6, CMOC 13).

Evidence from the United States suggests that institutional racism is not easily defined and encompasses not only individual prejudices, but prejudices deeply embedded within the law, practice, economy, culture and society which result in unequal access to healthcare, including health research.[74] The role of institutional racism in health research may be fuelled by the costs for translation and additional culturally sensitive resources affecting the chances of receiving funding for grant applications.[52] However, there is little evidence available on the extent to which institutional racism occurs in health research. A systematic review found that it is a concept that is rarely explicitly named in abstracts and titles and rarely engaged with; lack of acknowledgement of its existence could be holding back discovery within this field.[64-75]

Programme Theory

A resultant programme theory was produced following the five steps shown in figure 4. The programme theory informed discussions with stakeholders to develop recommendations and actions (Table 1) for individuals, organisations, institutions and policymakers to improve the informed consent process.

Table 1. Recommendations

For peer review only - http://bmjopen.bmj.com/site/about/guidelines.xhtml

**Recommendation**	**Action required**
**1. Policymakers, organisations, institutions and individuals need to take responsibility and address institutional racism, identifying and tackling issues that contribute to this (e.g., lack of culturally adapted information, practices that may be contributing to institutional racism.)**	This requires:<br>- leadership<br>- identification of best practice<br>- open discussion of culturally sensitive research ethics throughout the social system (e.g., values, beliefs and practices)<br>Organisations and institutions should inform and educate their staff about institutional racism, what it is and how to address it.
**2. Build relationships with communities from diverse cultural and ethnic backgrounds to provide opportunities for Patient and Public Involvement (PPI) and empower individuals so that study designs become more relevant and culturally appropriate.**	Employ more staff from diverse ethnic and cultural backgrounds in academic institutions and health research organisations.<br>Ethics committees should seek community members from diverse ethnic and cultural backgrounds.<br>Provide cultural competency training for all involved in health research.<br>Identify local champions and/or leads to raise awareness of cultural competency in health research.
**3. Develop tailored informed consent in health research that offers choices (e.g., written, audio, multi-media), which may require additional resources.**	Ensure greater Patient and Public Involvement (PPI) by people of diverse ethnic and cultural backgrounds in study designs.
**4. Organisations and institutions need to develop cultural competence within them,**	This requires cultural competence training that covers capability, knowledge and

For peer review only - http://bmjopen.bmj.com/site/about/guidelines.xhtml

**increasing capability, knowledge and awareness of other cultures.**	awareness in relation to research participation.<br><br>All who are involved in health research should undertake cultural competency training.

DISCUSSION

Statement of principal findings

This realist review of 71 sources summarises and expands upon the existing evidence around decision-making and participation in health research through focussing upon the context and mechanisms surrounding decision-making and understanding. The evidence base on participation in health research has tended to focus on the psychosocial drivers of participation, but use of a realist approach enabled greater consideration of contextual factors that influence whether participation occurs.

Through deeper exploration using iterative literature searching, data emerged that indicated the use of complex study documentation e.g., patient information leaflets, communication barriers, lack of social presence in the community by researchers and a lack of diversity amongst researchers as key realist mechanisms which led to an explanation of the interaction with context to influence outcomes.[2,8,54,72] Whilst the review team itself was not ethnically diverse, the stakeholder group were ethnically, professionally and organisationally diverse. EH is part of an Equality, Diversity, Inclusion working group which supported engagement with patient and public stakeholders from diverse cultural and ethnic backgrounds.

At the individual level (micro) level, empowerment through access to resources, such as culturally adapted information, and the opportunity to be involved in the design of health research form a significant aspect of the programme theory. At the interpersonal and

For peer review only - http://bmjopen.bmj.com/site/about/guidelines.xhtml

institutional (meso) levels, enabling shared decision making can result in positive outcomes through approaches such as co-production; building community relationships and provision of researcher-matching and/or culturally competent researchers provides assurance and increases trust.

The differences and similarities between the use of the term shared decision making in clinical care and within a health research context would benefit from additional exploration of the literature. The term shared decision making is defined by the UK's National Institute for Health and Care Excellence (NICE) shared decision making collaborative as:

> *a process in which clinicians and patients work together to select tests, treatments, management or support packages, based on clinical evidence and the patient's informed preferences. It involves the provision of evidence-based information about options, outcomes and uncertainties, together with decision support counselling and a system for recording and implementing patients' informed preferences.*[76-77]

It is of key importance in protecting patient/participant autonomy and voluntariness.[78-79] In the context of this review, shared decision making is part of the informed consent process and requires establishing understanding by the potential participant, discussion of the risks and benefits, as well as clarifying the consequences of different options available.[79] Shared decision making is also a vital element of co-production, or the involvement of patients and the public in shaping research projects.[80] In some instances, shared decision making can result in negative, or positive outcomes; for example, the hierarchies within some families may control decision-making through preventing or granting permission for family members to take part in health research.[6,9] More positive, shared decision making occurs when researchers spend time with communities, providing opportunities for informal discussions about research, building trust whilst developing cultural sensitivity to the "*norms and values*" of particular groups.[26]

For peer review only - http://bmjopen.bmj.com/site/about/guidelines.xhtml

Resources at the macro level are lacking, such as the lack of flexibility in approaches to the informed consent process, leading to negative outcomes with needs not being met and exclusion from health research, which may be perceived as institutional racism.[46,52,58,75] The data synthesis together with the stakeholder group meetings framed an overall programme theory in realist terms as:

> *If* organisations, institutions and individuals work together with diverse ethnic and cultural communities and become culturally competent, provide adaptive processes and empower community members to become involved , *then* this will develop and result in trust, better engagement, shared decision making and improved understanding.

The programme theory from this realist review is likely to have wider applicability despite being carried out in the context of the UK health care system. The informed consent process is a universal cornerstone to ethical practice in health research although may vary in the way in which it is carried out. However, rigid study designs and delivery may be contributing to the global issue of under-representation of under-served populations in health research.[18,50] While the critical issues described in this review are likely to be suitable for consideration by health research systems outside of the UK, it is recognised that resources and context differ across research systems, which may limit the transferability, particularly in low-income countries. The recommendations in this realist review requires evaluation in different settings to understand their wider applicability and transferability.

Strengths and weaknesses in relation to other studies

Using a realist approach enabled a wider overview of more hidden factors that affect the decision-making process in health research, regarding the informed consent process with people of diverse ethnic and cultural backgrounds. The realist review has drawn from both peer-reviewed literature (66,93%) and grey literature (5,7%) as well as bringing in expertise

For peer review only - http://bmjopen.bmj.com/site/about/guidelines.xhtml

from the stakeholder group. The lack of literature available on health research in general with people of diverse ethnic and cultural backgrounds is a weakness as more specific evidence about what works, for whom and under what circumstances is urgently needed in order to help researchers design studies that are more inclusive and inform service development.[3,5] It is acknowledged that not all sources may have been discovered throughout this realist review process, in addition to not including the expertise of policymakers due to restrictions on time.

The review was conducted in accordance with RAMESES quality and publication standards to ensure transparency and is viewed as a strength.[19,43]

The involvement of NIHR (Public) Research Champions, healthcare professionals, clinical academics and PPI Lead in refining the programme theory was felt to enhance the review's relevance. Having a second reviewer can increase the number of relevant studies included in a review and therefore this was considered a strength.[5]

Suggestions for further research

From the review, cultural competence emerged as a recurring mechanism within the literature with further gaps in the evidence around : the way in which studies are designed and delivered and how people from under-served populations are involved in this aspect of the research process; institutional racism and its impact on health research; experiences of participation in health research with people of diverse ethnic and cultural backgrounds; the best adaptive informed consent processes, particularly for people of diverse ethnic and cultural backgrounds . In addition to this, further research is required to understand more the full range of requirements that may be needed to develop a more inclusive informed consent process, alongside in-depth enquiry around cultural competence and researcher-matching in relation to outcomes about research participation.

CONCLUSION

For peer review only - http://bmjopen.bmj.com/site/about/guidelines.xhtml

The resultant programme theory explains how, when, why and for whom the processes involved in consenting to participate in health research does and does not work, and leads to suggestions for improvements to achieve greater inclusivity with people of diverse ethnic and cultural backgrounds.

Some aspects of the programme theory (e.g., institutional racism, cultural competency in health research, researcher-matching) need more research to better understand how they affect outcomes. A critical finding is that a research infrastructure that supports and mandates inclusion is needed, as well as additional resources to support adaptive processes, support shared decision making through involving more patients and the public in the design of health research studies. A greater focus on the evidence base developing in the field of global health community engagement, particularly around co-production, may provide valuable insights into how patients and the public from under-served communities can become involved in the design of health research studies.[80]

Main document

Figure 1. Theory Map – Factors influencing decisions about whether to participate in health research with under-served populations – identified from scoping search

Figure 2. Theoretical concepts compounding positively, or negatively, the intervention

Figure 3. Flow diagram of searching

Figure 4. Final programme theory

online supplementary file 1 – Scoping searches

online supplementary file 2 – Searches

online supplementary file 3 – Inclusion/Exclusion criteria

online supplementary file 4 – CMOC Long List

LIST OF ABBREVIATIONS

NIHR National Institute for Health Research

PPI Patient and Public Involvement

CMOC Context Mechanism Outcome Configuration

UCL IRIS University College London Institutional Research Information System

IHE Institute for Health Equality

DECLARATIONS

Funding statement

This work was supported by a National Institute for Health Research (NIHR), HEE/NIHR ICA Programme Pre-doctoral Clinical Academic Fellowships *grant number* NIHR 300317, as a Masters in Health Research dissertation. Funding for the open access charges for the publication of this protocol was provided by the NIHR Clinical Research Network West Midlands.

SS is part funded by the NIHR Applied Research Collaboration (ARC) West Midlands, the NIHR Health Protection Research Unit (HPRU) Gastrointestinal Infections, and the NIHR HPRU Genomics and Enabling Data.

Disclaimer The views expressed in this publication are those of the author(s) and not necessarily those of the NIHR, HEE, NHS or the UK Department of Health and Social Care.

Competing Interests None declared.

Ethics approval and consent to participate Not applicable**.**

Consent for publication. Not applicable.

For peer review only

For peer review only - http://bmjopen.bmj.com/site/about/guidelines.xhtml

Availability of data and materials. Not applicable.

Competing interests. None declared.

Author contributions EH was the lead reviewer. G H-B completed second reviewer tasks. SS, JD, G H-B critically reviewed and edited the manuscript. EH was responsible for the initial design and drafting of the manuscript. All authors read and approved the final manuscript.

Acknowledgements We thank Samantha Johnson Information Specialist, for critically appraising the search strategy. We would also like to thank the following stakeholders involved in this review for their contribution to improving the quality and relevance of the final programme theory as well as the recommendations: Anne Devrell, Geoff Robson, Ifeanyi Sargeant, Mohini Samini, Dr. Mohammed Shaikh, Richard Stephens, Sandra Prew, Dr. Farhana Lockhat, Dr. Emma Sutton , Zi Liew, Sarah Joshi, Claire Talbot, Jonathan Heffernan-Davies, Sylvia Turner, Mohamed Mooradun.

Patient consent for publication Not required.

Data sharing statement This review presents previously published and publicly available data. Please refer to the reference list and their authors for these research data.

References

1 Witham M, Anderson E, Carroll C, et al. Developing a roadmap to improve trial delivery for under-served groups: results from a UK multi-stakeholder process. *Trials*. 2020. doi.org/10.1186/s13063-020-04613-7 [published First Online 1 Aug 2020]

2 Redwood S, Gill P. Under-representation of minority ethnic groups in research- call for action. *Br J Gen Pract*. 2013. doi:10.3399/bjgp13X668456 [published Jul 2013]

3 Jutlla, K, Raghavan R. Improving the recruitment of Black, Asian and Minority Ethnic (BAME) communities in health and social care research: a

For peer review only - http://bmjopen.bmj.com/site/about/guidelines.xhtml

review of literature 2017.www.dora.dmu.ac.uk/handle/2086/13221 (accessed 28 Jun 2020)

4 Verastegui, E.Consenting of the vulnerable: The informed consent procedure in advanced cancer patients in Mexico. *BMC medical ethics*. 2006 doi:org/10.1186/1472-6939-7-13 [published Online First: 13 December 2006]

5 Vickers T, Craig G, Atkin K. Addressing Ethnicity in Social Care Research *Social Policy and Administration47(3):310 – 326* doi:org/10.1111/j.1467-9515.2012.00851.x [published Online First 24 May 2012]

6 Lloyd, C, Johnson, M, Mughal, S. Securing recruitment and obtaining informed consent in minority ethnic groups in the UK. BMC *Health Serv Res*.2008; 8: 68.doi:org/10.1186/1472-6963-8-68 [published Online First 30 Mar 2008]

7 Masood Y, Bower P, Waheed M, et al.Synthesis of researcher reported strategies to recruit adults of ethnic minorities to clinical trials in the United Kingdom: A systematic review. *Contemp Clin Trials*2019; 78:1-10. doi: 10.1016/j.cct.2019.01.004 [published First Online 8 Jan 2019]

8 Barata P, Guicciardi E, Ahmad F et al.Cross-cultural perspectives on research participation and informed consent. *Social Science & Medicine.*2006;62:479-www.academia.edu/9183270/Cross_cultural_perspectives_on_research_participation_and_informed_consent (accessed 01 Jun 2020)

9 Culley L, Hudson N, Rapport F. Using Focus Groups with Minority Ethnic Communities: Researching Infertility in British South Asian Communities. *Qualitative Health Research*. 2007;17(1):102-112. doi:10.1177/1049732306296506 [published First Online 1 Jan 2007]

10 Smart A, Harrison E. The under-representation of minority ethnic groups in UK medical research. *Ethn Health*. 2017 Feb;22(1):65-82. doi: 10.1080/13557858.2016.1182126 [published First Online 13 May 2016]

11 ICH .ICH E8 General considerations for clinical studies.2019.www.ema.europa.eu/en/ich-e8-general-considerations-clinical-studies (accessed Jun 2021)

12 Verastegui E. Consenting of the vulnerable: the informed consent procedure in advanced cancer patients in Mexico *BMC Medical Ethics.* 2006;7:13 doi:10.1186/1472-6939-7-13 [published First Online 13 Dec 2006]

13 Riesa N, Mansfield E, Sanson-Fisher R. Ethical and legal aspects of research involving older people with cognitive impairment: A survey of dementia researchers in *Australia International Journal of Law and Psychiatry. 2019;68.*doi.org/10.1016/j.ijlp.2019.101534 [published Feb 2020]

14 Smith J. How ethical is ethical research? Recruiting marginalized, vulnerable groups into health services research. *Journal of Advanced Nursing* 2008;62(2), 248–257. doi: 10.1111/j.1365-2648.2007.04567.x [published First Online 3 Apr 2008]

15 Zion D, Briskman L, Loff B. Returning to History: The Ethics of Researchign Asylum Seeker Health in Australia . *The American Journal of Bioethics*.2010;

For peer review only - http://bmjopen.bmj.com/site/about/guidelines.xhtml

10(2). doi.org/10.1080/15265160903469310 [published First Online 3 Feb 2010]

16 Atkin K. Institutional racism, policy and practice. In: Primary Healthcare and South Asian Populations–Meeting the Challenges. Oxford: Radcliffe Medical Press. 2004:1-12

17 Flory J, Emanuel E. Interventions to improve research participants' understanding in informed consent for research: a systematic review 2004. *JAMA* 292(13):1593-601

18 Nishimura A, Carey J, Erwin P. Improving understanding in the research informed consent process: a systematic review of 54 interventions tested in randomised control trials. *BMC Medical Ethics*. [online] 2013*:*14:28 Available from : .doi:10.1186/1472-6939-14-28 (accessed 15 Jun 2020)

19 Montalvo W, Larson E. Participant comprehension of research for which they volunteer: a systematic review. *J Nurs Scholarsh*. [online]2014;46(6):423-31. Available from : doi: 10.1111/jnu.12097. (accessed 30 Jun 2020)

20 Pawson R, Greenhalgh T, Harvey G, et al. Realist Review - a new method of systematic review designed for complex policy interventions. *J Health Serv Res Policy*. 2005; 1:21-34.doi:10.1258/1355819054308530 [published 1 Jul 2005]

21 Pawson, R, Greenhalgh, T, Harvey, G et al. 2004 *Realist Synthesis: An Introduction.* RMP Methods Paper 2/2004. www.researchgate.net/publication/228855827_Realist_Synthesis_An_Introduction/link/0fcfd507f0b7cbb2ce000000/download (accessed May 2020)

22 Hoverd E, Staniszewska S, Dale J. The informed consent process in health research with under-served populations: a realist review protocol. *Syst Rev.* 2021;10: 103. doi.org/10.1186/s13643-021-01652-2 [published First Online 9 Apr 2021]

23 Wong G, Greenhalgh T, Westhorp G. et al. RAMESES publication standards: realist syntheses. *BMC Med.2013;* 11:21 . doi.org/10.1186/1741-7015-11-21[published First Online29 Jan 2013]

24 National Institute for Health Research. Research Champions 2020.www.nihr.ac.uk/patients-carers-and-the-public/i-want-to-help-with-research/research-champions.htm (accessed 20 Apr 2020)

25 Emmel N, Greenhalgh J, Manzano A, et al. *Doing Realist Research*. London: Sage 2018.

26 Calamaro CJ. Culture competence in research: research design and subject recruitment. *J Pediatr Health Care.* 2008 Sep-Oct;22(5):329-32. doi: 10.1016/j.pedhc.2008.05.007 [published 1 Sep 2008]

27 Francis-Graham S, Ekeke N, Nelson C, et al. Understanding how, why, for whom, and under what circumstances opt-out bloodborne virus testing programmes work to increase test engagement and uptake within prison: a rapid-realist review *BMC Health Services Research* .2019;19:152 doi.org/10.1186/s12913-019-3970-z [published First Online 8 Mar 2019]

For peer review only - http://bmjopen.bmj.com/site/about/guidelines.xhtml

28 Garrison, N Sathe, N, Antommaria, A *et al.* A systematic literature review of individuals' perspectives on broad consent and data sharing in the United States. *Genet Med* 2016;18, 663–671. doi.org/10.1038/gim.2015.138 [published First Online 19 Nov 2015]
29 Hill, C Tawiah-Agyemang, S, Odei-Danso, B et al.Informed consent in Ghana: what do participants really understand? *Med Ethics* 2008;34:48–53. doi:10.1136/jme.2006.019059 [published First Online 21 Dec 2007]
30 Hughson J, Woodward-Kron R, Parker A, et al. A review of approaches to improve participation of culturally and linguistically diverse populations in clinical trials *Trials*. 2016 doi:10.1186/s13063-016-1384-3 [published First Online 26 May 2016]
31 Locher J,Bronstein J, Robinson et al. Ethical Issues Involving Research Conducted With Homebound Older Adults The Gerontologist *The Gerontological Society of America;*2006; 46: 2, 160–164. doi.org/10.1093/geront/46.2.160 [published First 1 Apr 2006]
32 Munthe C, Radovic S, Anckarsater H.Ethical Issues in forensic psychiatric research on mentally disordered offenders. *Bioethics*2010;24(1):34-35. doi:10.1111/j.1467-8519.2009.01773.x [published First Online 11 Dec 2009]
33 Mystakidou K ,Panagiotou I. Ethical and practical challenges in implementing informed consent in HIV/AIDS clinical trials in developing or resource-limited countries, SAHARA-J: *Journal of Social Aspects of HIV/AIDS*, 2009;6:2, 46-57. doi.org/10.1080/17290376.2009.9724930 [published First Online 28 Feb 2009]
34 Shepherd, V, Wood, F, Griffith, R et al. Protection by exclusion? The (lack of) inclusion of adults who lack capacity to consent to research in clinical trials in the UK .*Trials.* 2019;20:474. doi.org/10.1186/s13063-019-3603-1[published First Online 5 Aug 2019]
35 Vallely L, Lees S, Shagi C et al. How informed is consent in vulnerable populations? Experience using a continuous consent process during the MDP301 vaginal microbicide trial in Mwanza, Tanzania *BMC Medical Ethics* 2010;11:10 .www.biomedcentral.com/1472-6939/11/10 (accessed Jul 2020)
36 Lakes, K, Vaughan, E, Jones, M.et al. Diverse perceptions of the informed consent process: implications for the recruitment and participation of diverse communities in the National Children's Study. *American journal of community psychology* 2012; 49(1-2), 215–232. doi.org/10.1007/s10464-011-9450-1[published First Online 14 Jun 2011]
37 Folayan M, Haire B, Harrison A, et al.Ethical Issues in Adolescents' Sexual and Reproductive Health Research in Nigeria. *Dev World Bioeth.* 2015;15(3):191-8. doi: 10.1111/dewb.12061[published First Online 9 Jun 2014]
38 Kantilal K, Hardeman W, Whiteside H, et al. Realist review protocol for understanding the real-world barriers and enablers to practitioners implementing self-management support to people living with and beyond

For peer review only - http://bmjopen.bmj.com/site/about/guidelines.xhtml

cancer *BMJ Open* 2020;10. doi: 10.1136/bmjopen-2020-037636 [published First Online 3 Sep 2020]

39 Malek- Ahmadi M. Macro-Level, Meso-Level, and Micro-Level Analysis 2020.www.coursehero.com/sg/introduction-to-sociology/macro-level-meso-level-and-micro-level-analysis/ (accessed Aug 2020)

40 Moore T, McArthur M, Noble-Carr D. More a marathon than a hurdle: towards children's informed consent in a study on safety. *Qualitative Research* 2018;18(1):88-107. doi:10.1177/1468794117700708 [published First Online 2 May 2017]

41 Vickers T, Craig G, Atkin K. Research with black and minority ethnic people using social care services. SSCR methods review, 2012;11. NIHR School for Social Care Research, London, UK.www.eprints.lse.ac.uk/43161/1/SSCR_Methods_Review_11_(lsero).pdf (accessed Apr 2020)

42 Stoll C, Izadi S, Fowler S et al. The value of a second reviewer for study selection in systematic reviews *Research synthesis methods*. 2019;10(4):539-545. doi.org/10.1002/jrsm.1369 [published First Online 4 Jul 2019]

43 Rycroft - Malone J, McCormack B, Hutchinson A. Realist synthesis: illustrating the method for implementation research. *Impl Science*. 2012;doi:10.1186/1748-5908-7-33 [published First Online 19 Apr 2012]

44 Scharff D, Mathews K, Jackson P, et al. More than Tuskegee: understanding mistrust about research participation. *J Health Care Poor Underserved*. 2010;21(3):879-897. doi:10.1353/hpu.0.0323 [published First Online Aug 2010]

45 Kao C, Aranda S, Krishnasamy M, et al. Identifying essential information to support patient decision-making regarding participation in cancer clinical trials: A Delphi study. *Eur J Cancer Care* (Engl). 2018 ;27(6).doi: 10.1111/ecc.12954 [published First Online 17 Sep 2018]

46 Grady, C, Hampson, L, Wallen, G et al. Exploring the ethics of clinical research in an urban community. *American Journal of Public Health,* 2006;96(11). doi.org/10.2105/AJPH.2005.071233 [published First Online 10 Oct 2011]

47 McDougall G, Simpson G, Friend M. Strategies for research recruitment and retention of older adults of racial and ethnic minorities. *J Geront Nurs*. 2015. doi.org/10.3928/00989134-20150325-01 [published First Online 25 Mar 2015]

48 Heywood R, Mackaskill A,Williams K. Informed consent in hospital practice: health professionals' perspectives and legal reflections.*Medical Law Review,* 2010; 18 (2).www.pubmed.ncbi.nlm.nih.gov/20513795/ (accessed May 2020)

49 Mchale J. Innovation, informed consent, health research and the Supreme Court: Montgomery v Lanarkshire - a brave new world? *Health Econ Policy Law*. 2017;12(4):435-452. doi: 10.1017/S174413311700010X. [published First Online 27 Apr 2017]

For peer review only - http://bmjopen.bmj.com/site/about/guidelines.xhtml

50 Treweek S, Forouhi N, Narayan K,et al .COVID-19 and ethnicity:who will research results apply to? *The Lancet* 2020. 395(10242):1955.doi: 10.1016/S0140-6736(20)31380-5 [published First Online 12 Jun 2020]

51 Jolly K, Lip G, Taylor R,et al. Recruitment of ethnic minority patients to a cardiac rehabilitation trial: the Birmingham Rehabilitation Uptake Maximisation (BRUM) study *BMC medical research methodology* 2005;*5*, 18. doi.org/10.1186/1471-2288-5-18 [published First Online 17 May 2005]

52 Hussain-Gambles M, Atkin K, Leese B. South Asian participation in clinical trials: the views of lay people and health professionals. *Health Policy.* 2006 ;77(2):149-65. doi: 10.1016/j.healthpol.2005.07.022.[published First Online 10 Oct 2005]

53 Kleinman A, Benson P. Anthropology in the Clinic: The Problem of Cultural Competency and How to Fix It. *PLoS Med* 2006;3(10). doi.org/10.1371/journal.pmed.0030294 [published First Online 24 Oct 2006]

54 Kirby T. Evidence mounts on the disproportionate effect of COVID-19 on ethnic minorities. *The Lancet Respiratory Medicine*.2020.doi:10.1016/S2213-2600(20)30228-9 [published First Online 8 May 2020]

55 McDougall G, Simpson G, Friend M. Strategies for research recruitment and retention of older adults of racial and ethnic minorities. *J Geront Nurs.* 2015.doi:10.3928/00989134-20150325-01 [published First Online 30 Mar 2015]

56 Ries N, Thompson K, Lowe M. Including People with Dementia in Research: An Analysis of Australian Ethical and Legal Rules and Recommendations for Reform. *J Bioeth Inq.* 2017;14(3):359-374. www. //psycnet.apa.org/record/2017-38470-007 (accessed Apr 2020)

57 Dawson, S, Campbell S, Giles S et al. Black and minority ethnic group involvement in health and social care research: A systematic review. *Health expectations: an international journal of public participation in health care and health policy* 2018;21(1): 3-22. doi:10.1111/hex.12597 [published First Online 15 Aug 2018]

58 Wiles, R, Charles V, Crow G et al. ‘Researching researchers: lessons for research ethics. *Qualitative Research*, 2006;6(3): 283–299.www. https://eprints.soton.ac.uk/40739/ (accessed Aug 2021)

59 Hamel L, Penner L, Albrecht T, et al. Barriers to Clinical Trial Enrollment in Racial and Ethnic Minority Patients With Cancer. *Cancer Control.* 2016;23(4):327-337. doi:10.1177/107327481602300404 [published First Online 1 Oct 2016]

60 Chavez V. Cultural humility: Reflections and relevance for CBPR. In Wallerstein, N., Duran, B., Oetzel, J., Minkler, M. (Eds.), Community-based participatory research for health: Advancing social and health equity 2018; 357-362. [doi.org/10.1177/1524839919884912 [published First Online 12 Nov 2019]

For peer review only - http://bmjopen.bmj.com/site/about/guidelines.xhtml

61 Erves J, Mayo-Gamble T, Malin-Fair A, et al. Needs, Priorities, and Recommendations for Engaging Underrepresented Populations in Clinical Research: A Community Perspective. *J Community Health*. 2017 ;42(3):472-480. doi: 10.1007/s10900-016-0279-2. [published First Online 3 Nov 2016]

62 Cheah P, Parker M. Research consent from young people in resource-poor settings. *Archives of disease in childhood.*2014. doi:10.1136/archdischild-2014-307121.[published May 2015]

63 Cross T, Bazron B, Dennis K, et al. Towards a culturally competent system of care.1989;Georgetown University Child Development Center, CASSP Technical Assistance Center, Washington, DC. www. https://files.eric.ed.gov/fulltext/ED330171.pdf (accessed May 2020)

64 Jongen C, McCalman J, Bainbridge R. Health workforce cultural competency interventions: a systematic scoping review. *BMC Health Serv Res* 2018;18(232) . doi.org/10.1186/s12913-018-3001-5 [published First Online 2 Apr 2018]

65 Minkler M, Pies C, Hyde C. Ethical issues in community organizing and capacity building. In Minkler, M. (Ed.), Community organizing and community building for health and welfare .New Brunswick, NJ: Rutgers University Press. 2012:110-129

66 Hanif W, Ali S, Patel K et al.Cultural competence in Covid-19 vacine rollout *BMJ* 2020;371:4845 doi:10.1136/bmj.m4845.*doi: 10.1136/bmj.m4845* [published First Online 18 Dec 2020]

67 National Institute of Healthcare (NIH). 2019;Cultural respect.www.nih.gov/institutes-nih/nih-office-director/office-communications-public-liason/clear-communication/cultural-respect (accessed Apr 2020)

68 Ahikari B, Vincent R, Wong, G. A realist review of community engagement with health research. *Wellcome Open Research* 2019; 4(87). doi: 10.12688/wellcomeopenres.15298.2 [published First Online 2 Aug 2019]

69 Fiske A, Prainsack B, Buyx A. *J Med Ethics* 2019;45:617–622. doi:10.1136/medethics-2018-105253 [published First Online 12 Jul 2019]

70 Edge D, Grey P. An Assets-Based Approach to Co-Producing a Culturally Adapted Family Intervention (CaFI) with African Caribbeans Diagnosed with Schizophrenia and Their Families. *Ethnicity & disease*, *28*(Suppl 2), 485-492.www.ethndis.org/edonline/index.php/ethndis/article/view/951 (accessed Sept 2020)

71 Nnaji C, Boone M, Pugnaire MP, et al.An Innovative Simulation-based Community-engaged Intervention for Training Research Assistants in Culturally Appropriate Informed Consent. *Prog Community Health Partnersh*. 2018;12(3):247-262. doi: 10.1353/cpr.2018.0049 [published First Online 28 Nov 2017]

72 Kadam R. Informed consent process: A step further towards making it meaningful! *Perspectives in Clinical Research*. 2017; 8(3):107-12. doi10.4103/picr.PICR_147_16 [published Sept 2017]

For peer review only - http://bmjopen.bmj.com/site/about/guidelines.xhtml

73 Grewal I and Ritchie J. Ethnic and language matching of the researcher and the research group during design, fieldwork, and analysis. In: Nazroo, J (ed) *Health and Social Research in Multiethnic Societies London:* Routledge :2006:1-17 www.taylorfrancis.com/chapters/edit/10.4324/9780203969939-12/ethnic-language-matching-researcher-research-group-design-%EF%AC%81eldwork-analysis-ini-grewal-jane-ritchie (accessed May 2020

74 Bailey Z, Feldman J, Bassett M. How Structural Racism Works — Racist Policies as a Root Cause of U.S. Racial Health Inequities. *New England Journal of Medicine* 2021;384, 768–773.doi:10.1056/nejmms2025396 [published First Online 25 Feb 2021]

75 Hardeman R, Murphy K, Karbeah J,et al. Naming Institutionalized Racism in the Public Health Literature: A Systematic Literature Review. Public Health Report . 2018;133(3): 240-249. doi: 10.1177/0033354918760574[published First Online 3 Apr 2018]

76 NICE *Shared decision making* NICE guideline [NG197]. www.nice.org.uk/guidance/ng197 [published 17 Jun 2021] (accessed Jan 2022)

77 Coulter A, Collins A. Making shared decision-making a reality: no decision about me, without me *The Kings Fund* 2011. www.kingsfund.org.uk/sites/default/files/Making-shared-decision-making-a-reality-paper-Angela-Coulter-Alf-Collins-July-2011_0.pdf (accessed 5 Jan 2022)

78 Moulton H, Moulton B, Lahey T, et al. Can Consent to Participate in Clinical Research Involve Shared Decision Making? *AMA J Ethics*. 2020 May 1;22(5):E365-371. www. pubmed.ncbi.nlm.nih.gov/32449651/ (accessed Dec 2021)

79 Childress J, Childress M *AMA J Ethics.*2020;22(5):E423-429. www.journalofethics.ama-assn.org/article/what-does-evolution-informed-consent-shared-decision-making-teach-us-about-authority-health-care/2020-05 (accessed Dec 2021)

80 Redman S, Greenhalgh T, Adedokun L et al.(2021) Co-production of knowledge: the future A new collection highlights the role of co-production in strengthening health systems *BMJ*.2021;372.doi.org/10.1136/bmj.n434 [published First Online 16 Feb 2021]

For peer review only - http://bmjopen.bmj.com/site/about/guidelines.xhtml

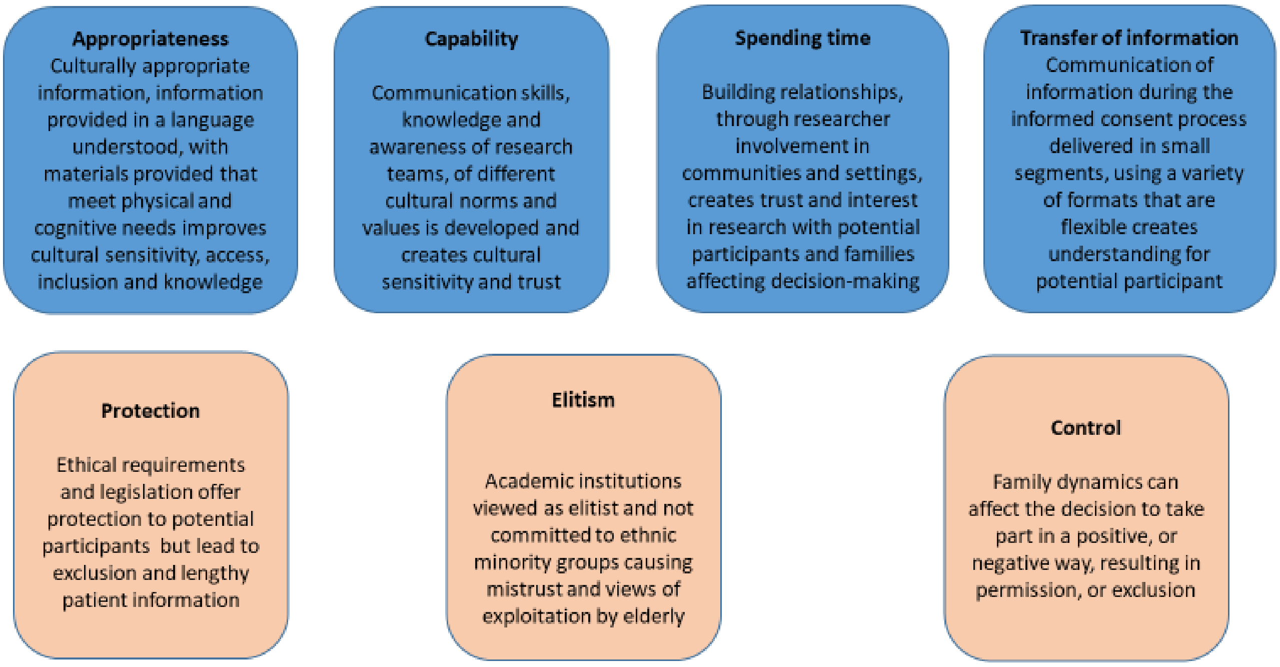

Figure 1. Theory Map –Factors influencing decisions about whether to participate in health research with under-served populations – identified from scoping search

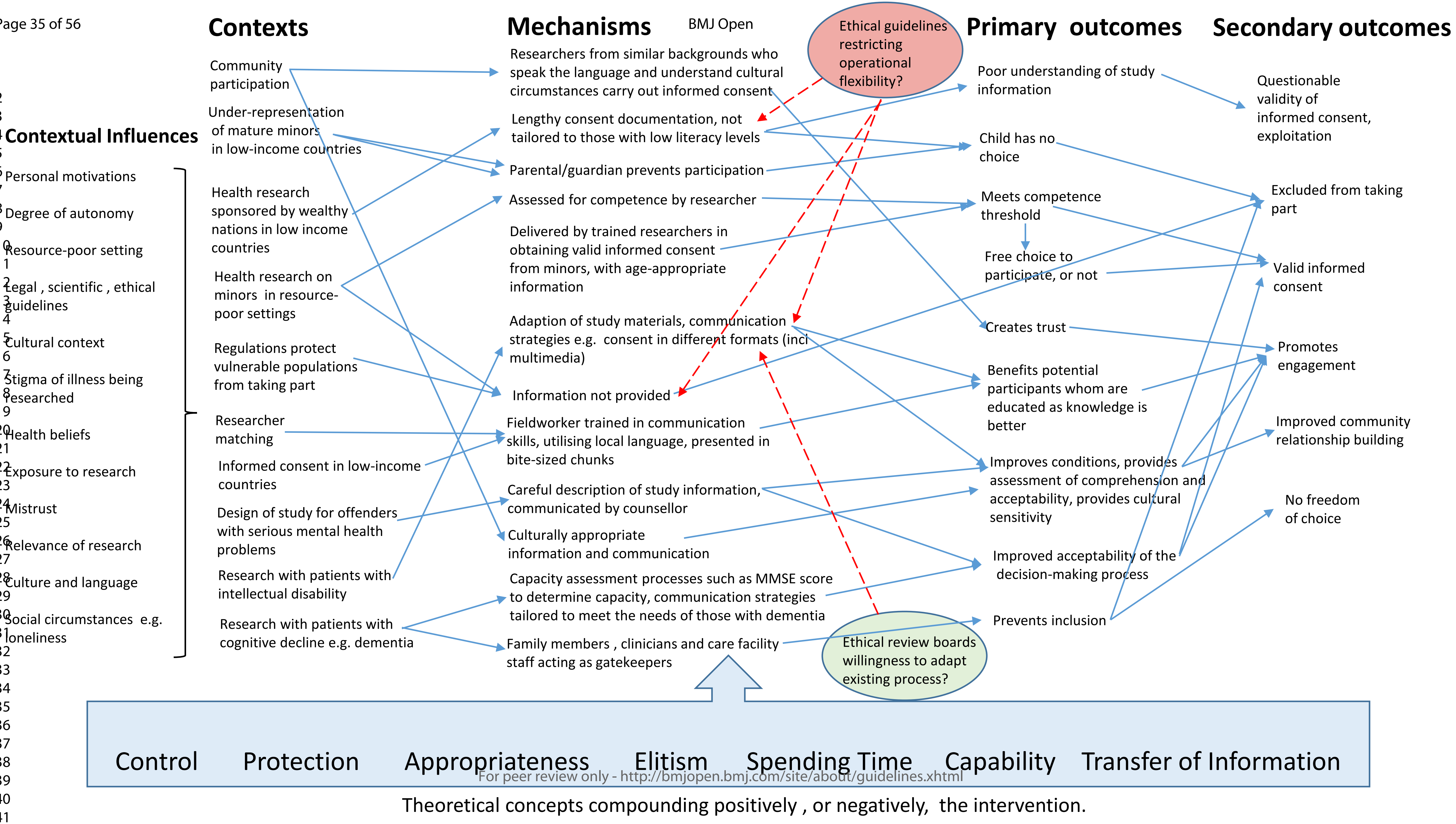
Contexts
Mechanisms
Primary outcomes
Secondary outcomes
Contextual Influences
Personal motivations
Degree of autonomy
Resource-poor setting
Legal , scientific , ethical guidelines
Cultural context
Stigma of illness being researched
Health beliefs
Exposure to research
Mistrust
Relevance of research
Culture and language
Social circumstances e.g. loneliness
Community participation
Under-representation of mature minors in low-income countries
Health research sponsored by wealthy nations in low income countries
Health research on minors in resource-poor settings
Regulations protect vulnerable populations from taking part
Researcher matching
Informed consent in low-income countries
Design of study for offenders with serious mental health problems
Research with patients with intellectual disability
Research with patients with cognitive decline e.g. dementia
Researchers from similar backgrounds who speak the language and understand cultural circumstances carry out informed consent
Lengthy consent documentation, not tailored to those with low literacy levels
Parental/guardian prevents participation
Assessed for competence by researcher
Delivered by trained researchers in obtaining valid informed consent from minors, with age-appropriate information
Adaption of study materials, communication strategies e.g. consent in different formats (incl multimedia)
Information not provided
Fieldworker trained in communication skills, utilising local language, presented in bite-sized chunks
Careful description of study information, communicated by counsellor
Culturally appropriate information and communication
Capacity assessment processes such as MMSE score to determine capacity, communication strategies tailored to meet the needs of those with dementia
Family members , clinicians and care facility staff acting as gatekeepers
Ethical guidelines restricting operational flexibility?
Ethical review boards willingness to adapt existing process?
Poor understanding of study information
Child has no choice
Meets competence threshold
Free choice to participate, or not
Creates trust
Benefits potential participants whom are educated as knowledge is better
Improves conditions, provides assessment of comprehension and acceptability, provides cultural sensitivity
Improved acceptability of the decision-making process
Prevents inclusion
Questionable validity of informed consent, exploitation
Excluded from taking part
Valid informed consent
Promotes engagement
Improved community relationship building
No freedom of choice
Control Protection Appropriateness Elitism Spending Time Capability Transfer of Information
Theoretical concepts compounding positively , or negatively, the intervention.

Flow diagram of searching

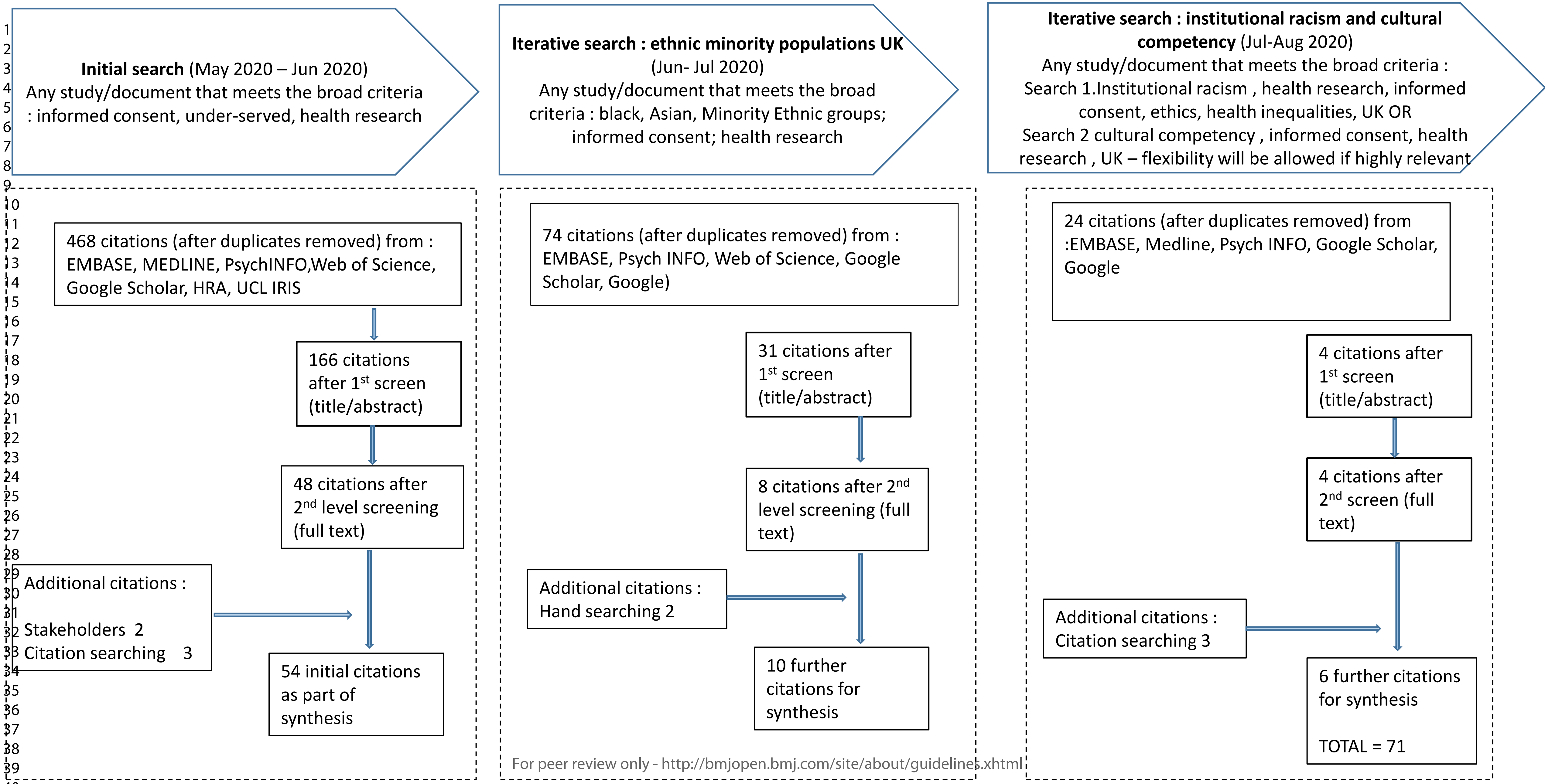

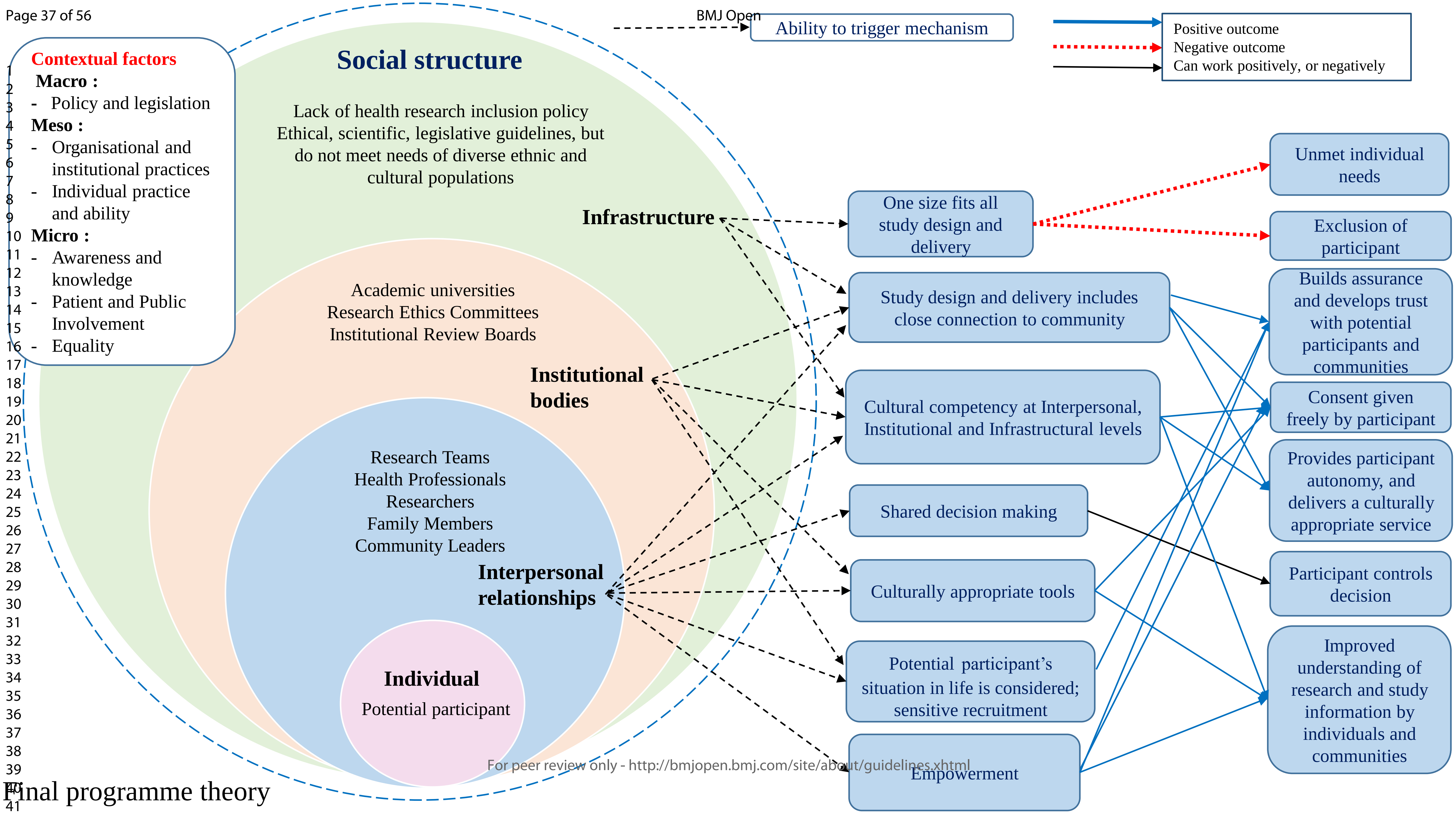

Final programme theory

Scoping Searches

Medline Scoping Search
Key terms informed consent, under-served , under-represented, health research, biomedical research 2005-current, English only

Database: Ovid MEDLINE(R) <1946 to May Week 2 2020>
Search Strategy:
----
1 informed consent.mp. or Informed Consent/ (57316)
2 exp Telemedicine/ or under-served.mp. or exp Vulnerable Populations/ (38247)
3 under-representation.mp. (756)
4 1 and 2 and 3 (0)
5 1 and 2 (820)
6 health research.mp. or exp Biomedical Research/ (277290)
7 5 and 6 (373)
8 limit 7 to (english language and yr="2005 -Current") (181)

****

Web of Science search May 21 2020

Key words : informed consent and under represented

Limits : English, 2005-current

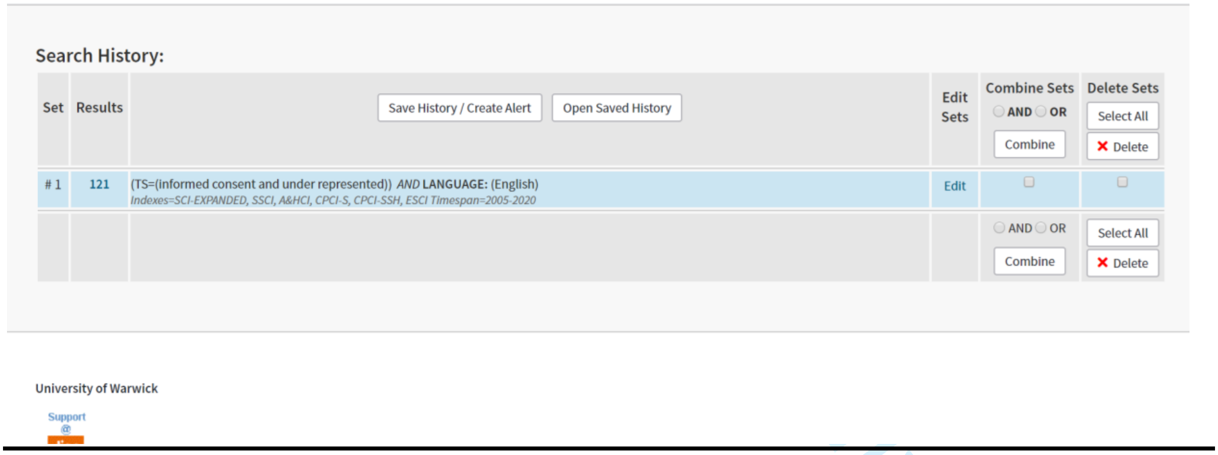

Database : UCL IRIS
Keywords : informed consent, under-served, health research, under represented

For peer review only - http://bmjopen.bmj.com/site/about/guidelines.xhtml

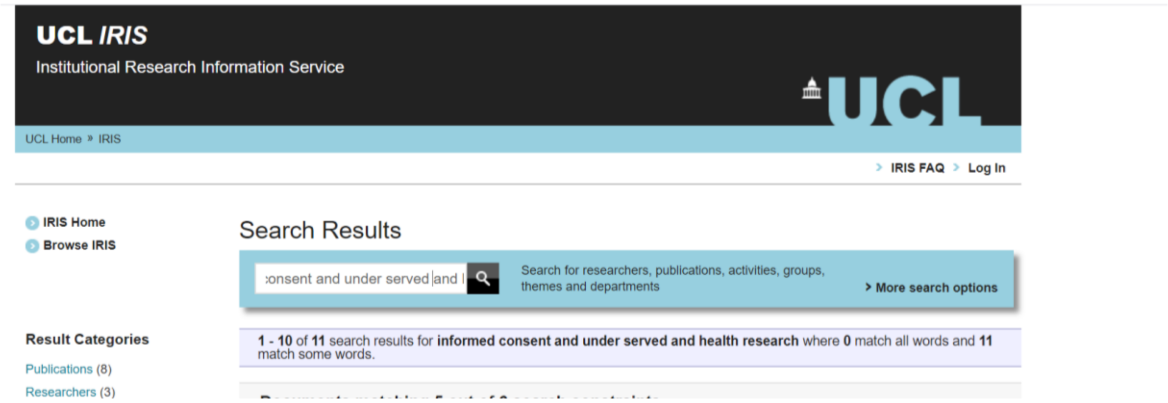

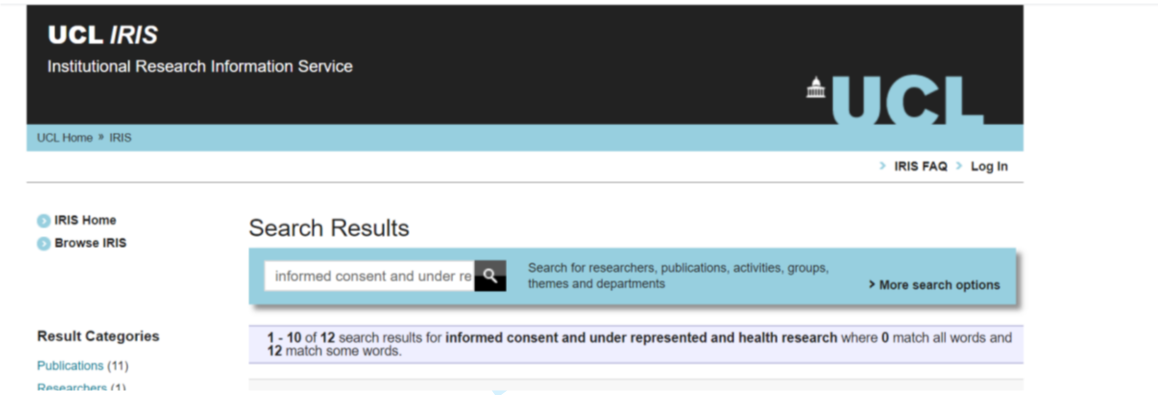

HRA (Health Research Authority)
Keywords: informed consent, underrepresented, health research

For peer review only

EMBASE SEARCH

Database: EMBASE <1947 to 2020 Week 20>

Search Strategy:

----

1 informed consent.mp. or exp informed consent/ (128719)

2 health research.mp. or exp medical research/ (433296)

3 1 and 2 (9419)

4 under served.mp. (587)

5 vulnerable populations.mp. or vulnerable population/ (20469)

6 under-served.mp. (587)

7 4 and 5 and 6 (27)

8 4 or 5 or 6 (21029)

9 3 and 8 (218)

10 limit 9 to english language (211)

****

Medline Search 1

Database: Ovid MEDLINE(R) <1946 to May Week 1 2020>

Search Strategy:

----

1 informed consent.mp. or exp Informed Consent/ (62033)

2 under served.mp. (312)

3 Health Services Research/ or health research.mp. or Biomedical Research/ (118031)

4 1 and 3 (2832)

5 under-served.mp. or exp Vulnerable Populations/ (10894)

6 4 and 5 (107)

****

Web of Science

TOPIC: (informed consent and under represented and health research)
Refined by: [excluding] PUBLICATION YEARS=(2004 OR 2003 OR 1993)

For peer review only

Timespan=All years. Indexes=SCI-EXPANDED, SSCI, A&HCI, CPCI-S, CPCI-SSH, ESCI.

"Title","Authors","Corporate Authors","Editors","Book Editors","Source Title","Publication Date","Publication Year","Volume","Issue","Part Number","Supplement","Special Issue","Beginning Page","Ending Page","Article Number","DOI","Conference Title","Conference Date","Total Citations","Average per Year","1900","1901","1902","1903","1904","1905","1906","1907","1908","1909","1910","1911","1912","1913","1914","1915","1916","1917","1918","1919","1920","1921","1922","1923","1924","1925","1926","1927","1928","1929","1930","1931","1932","1933","1934","1935","1936","1937","1938","1939","1940","1941","1942","1943","1944","1945","1946","1947","1948","1949","1950","1951","1952","1953","1954","1955","1956","1957","1958","1959","1960","1961","1962","1963","1964","1965","1966","1967","1968","1969","1970","1971","1972","1973","1974","1975","1976","1977","1978","1979","1980","1981","1982","1983","1984","1985","1986","1987","1988","1989","1990","1991","1992","1993","1994","1995","1996","1997","1998","1999","2000","2001","2002","2003","2004","2005","2006","2007","2008","2009","2010","2011","2012","2013","2014","2015","2016","2017","2018","2019","2020"

Database: APA PsycInfo <1806 to May Week 4 2020>

Search Strategy:

----

1 (under-served or underserved).mp. [mp=title, abstract, heading word, table of contents, key concepts, original title, tests & measures, mesh] (5394)

2 health research.mp. (5729)

3 informed consent.mp. or exp Informed Consent/ (11199)

4 biomedical research.mp. (5165)

5 (under represented or under-represented).mp. [mp=title, abstract, heading word, table of contents, key concepts, original title, tests & measures, mesh] (1120)

6 underserved.mp. (5126)

7 under served.mp. (294)

8 vulnerable population.mp. (2420)

9 6 or 7 (5394)

10 informed consent.mp. or exp Informed Consent/ (11199)

11 decision making.mp. or exp Decision Making/ (167677)

12 research.mp. or exp Experimentation/ (1223074)

13 10 or 11 or 12 (1339577)

14 9 and 13 (2287)

For peer review only

15 1 or 5 or 6 or 7 (6498)

16 2 and 3 (168)

Iteration 1- search

Database: APA PsycInfo <1806 to May Week 3 2020>

Search Strategy:

----

1 INFORMED CONSENT.mp. or exp Informed Consent/ (11049)

2 HEALTH RESEARCH.mp. (5578)

3 exp Blacks/ or exp At Risk Populations/ or UNDER-SERVED.mp. or exp Disadvantaged/ (94625)

4 1 and 2 and 3 (7)

Database: Embase Classic+Embase <1947 to 2020 Week 20>

Search Strategy:

----

1 informed consent.mp. or informed consent/ (131165)

2 health research.mp. or medical research/ (234000)

3 Black.mp. or Black person/ (178998)

4 At risk populations.mp. (3605)

5 Under-served.mp. (601)

6 disadvantaged.mp. (15612)

7 1 and 2 and 3 (20)

Web of Science 17	TOPIC: (informed consent and minority ethnic groups and health research)<br>**Refined by:** [excluding] **PUBLICATION YEARS:** (2004 OR 2002)<br>*Indexes=SCI-EXPANDED, SSCI, A&HCI, CPCI-S, CPCI-SSH, ESCI Timespan=All years*

Database: Ovid MEDLINE(R) <1946 to June Week 1 2020>

Search Strategy:

For peer review only

----

1 informed consent.mp. or Informed Consent/ (57978)

2 health research.mp. (12581)

3 Black.mp. (102740)

4 at risk populations.mp. (2115)

5 under-served.mp. or Vulnerable Populations/ (10932)

6 disadvantaged.mp. or Vulnerable Populations/ (20499)

7 1 and 2 and 3 (1)

8 2 and 5 (101)

9 1 and 8 (22)

****

Iteration 2 – Searches

(Search 1)

Database: Embase Classic+Embase <1947 to 2020 Week 35>

Search Strategy:

----

1 institutional racism.mp. (185)

2 health research.mp. or medical research/ (234000)

3 informed consent.mp. or informed consent/ (131165)

4 1 and 2 (3)

5 limit 4 to yr="2005 -Current" (1)

****

Database: APA PsycInfo <1806 to July Week 1 2020>

Search Strategy:

----

1 INSTITUTIONAL RACISM.mp. (453)

2 health research.mp. (5729)

For peer review only

3 informed consent.mp. or exp Informed Consent/ (11199)

4 1 and 2 (2)

5 limit 2 to yr ="2005-Current" (0)

Database : Web of Science July Week 1

Results: 2

(from Web of Science Core Collection)

You searched for: **TOPIC:** (institutional racism and health research and informed consent)

Timespan: All years. **Indexes:** SCI-EXPANDED, SSCI, A&HCI, CPCI-S, CPCI-SSH, ESCI.

Database: Ovid MEDLINE(R) <1946 to July Week 1 2020>

Search Strategy:

----

1 institutional racism.mp. (115)

2 Health Services Research/ or health research.mp. or Biomedical Research/ (118339)

3 1 and 2 (9)

4 limit 3 to yr="2005 -Current" (5)

(Search 2)

Database: Embase Classic+Embase <1947 to 2020 Week 25>

Search Strategy:

----

1 cultural competency.mp. or cultural competence/ (7186)

2 health research.mp. or medical research/ (234000)

3 1 and 2 (213)

4 informed consent.mp. or informed consent/ (131165)

5 3 and 4 (9)

Database: Ovid MEDLINE(R) <1946 to July Week 1 2020>

Search Strategy:

----

1 cultural competency.mp. or Cultural Competency/ (6133)

2 Health Services Research/ or health research.mp. or Biomedical Research/ (118339)

3 1 and 2 (196)

For peer review only

4 informed consent.mp. or Informed Consent/ (58028)

5 3 and 4 (8)

Database: APA PsycInfo <1806 to July Week 1 2020>

Search Strategy:

----

1 cultural competency.mp. (2952)

2 health research.mp. (5729)

3 informed consent.mp. or exp Informed Consent/ (11199)

4 1 and 2 and 3 (1)

Results: **9**

(from Web of Science Core Collection)

You searched for: **TOPIC:** (cultural competency and health research and informed consent)

Timespan: All years. **Indexes:** SCI-EXPANDED, SSCI, A&HCI, CPCI-S, CPCI-SSH, ESCI.

For peer review only

Inclusion/exclusion criteria

Inclusion criteria

- Quantitative and qualitative studies, grey literature, websites, stakeholder recommendations (e.g., reports, conference papers).
- The search will be limited to a 15-year publication period (2005-2020) due to the extent to which research infrastructure and research governance has changed over that period of time. Inclusion of review articles (published within the last 15 years) included evidence from earlier studies so that we were able to draw on earlier evidence where it was relevant. In addition to this, the evidence reviewed was sufficient to inform and develop programme theory which was the purpose of the review.
- All studies that report on experiences and decision-making involved in the informed consent process will be included.
- Any source of data that is deemed relevant by the stakeholder group.
- Literature that explores the concept and practice of informed consent.
- Only sources written in English will be included.

Exclusion criteria

- Studies or sources of data that are not relevant to the informed consent process in health research in relation to under-represented populations.

For peer review only - http://bmjopen.bmj.com/site/about/guidelines.xhtml

CMOC Long list

The CMOC's shaded blue are specific to people of diverse ethnic and cultural backgrounds in the UK. All other CMOC's include those from the initial programme theory.

CMOC	Context (How designed and delivered)	Mechanism (tailored, how info provided, delivered)	Outcome (decision-making)	Does it work?
CMOC1	C2 Meso level<br>Organisations and people within them should deliver services with cultural competence	M1 Skilled , culturally competent researchers Promotes positive and effective relationships with diverse communities . Researchers need to develop and employ sensitive skills to work with people from different cultural backgrounds	Meets the social, cultural, religious and linguistic needs of ethnic minority populations	yes
CMOC2	C1 Macro level<br>C2 Meso level<br>informed consent can be delivered in other formats apart from written	M4 Flexible processes, tailored information oral, audio-recorded study information and verbal consent	promotes feelings of independence;	yes
CMOC3	C2 Meso level<br>communities advertising research	M5 Community involvement shopkeepers and mosque leaders as advocates for research creates enthusiasm	influences decision to take part	yes

For peer review only

CMOC4	C2 Meso level appropriate matching of researcher to group being researched	M4 Flexible processes, tailored information consideration of the gender of the researcher due to religious reasons can affect engagement with discussion; matching ethnic background of researcher to ethnic background of participant	participant does not feel comfortable to discuss problems in some cases depending on gender of researcher, due to religious reasons; may prevent some participants from airing counter-cultural views	no
CMOC 5	M1 Macro level M2 Meso level	M4 Flexible processes, tailored information involving family, community members, or local health professionals to support shared decision making	shared decision making	yes
CMOC4	C2 Meso level ethnic matching	M4 Flexible processes, tailored information participant has some choice in which researcher they work with	effective and positive experience for potential participant, having a bearing on decision-making about taking part	yes
CMOC5	C1 Macro level C2 Meso level information provided only in English	M2 Removing/restricting choice considered institutionalised racism	excludes older ethnic minority populations and migrants	no
CMOC6	C1 Macro level C2 Meso level study design restricts access	M3 Inflexible process M2 Removing/Restricting choice	under-representation	no

For peer review only

		eligibility criteria excludes certain populations		
CMOC7	C2 Meso level commitment to confidentiality	M1 Skilled, culturally competent researchers researchers qualify confidentiality before obtaining consent, confirming comprehension	reduces fear of being reported to authorities (if migrant pop) and prevents decision of obedience to "community leaders"	yes
CMOC8	C1 Macro level<br>C3 Micro level<br>illiteracy, knowledge of health research, legal aspects	M3 Inflexible process signed informed consent , lacking in assurance of confidentiality	feel undue pressure to participate , or refuse due to fear of a negative impact on immigration process	no
CMOC9	M1 Macro level<br>M2 Meso level<br>study design	M1 Skilled, culturally competent researchers awareness of local protocol, legislation and culture; consideration of potential participants current situation in life; potential participant's awareness of the study; sensitive recruitment	culturally appropriate instruments , successful informed consent	yes

For peer review only

CMOC11	M3 Micro level<br>life situation of some populations e.g. migrant communities	M2 Removing/restricting choice<br>M3 Inflexible process<br>individual decision-making not possible due to family relationships	identification of key family/community member involved in decision making with improved	yes
CMOC12	M1 Macro level<br>M2 Meso level<br>design and delivery of study	M4 Flexible processes, tailored information<br>close connection to community - conversations are basis for informed consent , summarising information, explaining written information (this can be supported by traditional methods, witnessed consent, audio-recording, video-taping or illustrations)	consent given freely, respect for cultural differences, improves trust , is ethical and ensures dignity	yes
CMOC13	M1 Macro Level<br>M2 Meso level<br>wider health care system	M3 Inflexible processes<br>M2 Removing / restricting choice<br>policies of institutions<br>1. presumption that one size fits all<br>2. difference is acknowledged but needs are misrepresented	discriminatory outcomes (e.g. exclusion due to eligibility criteria); ignores needs of ethnically diverse populations; enforces inappropriate policy and responses by health professionals	no

For peer review only

**CMOC14**	**M1 Macro level M2 Meso level primary health care**	**M2 Removing/restricting choice gaining access institutional racism used as a framework in healthcare**	**helps to understand discrimination and disadvantages minority ethnic groups experience**	**yes**
**CMOC15**	**M1 Macro level low-income countries**	**M2 Removing/restricting choice parental/guardian control**	**results in exclusion from taking part**	**no**
**CMOC 16**	**M1 Macro level M2 Meso level mature minors (often under-represented)**	**M4 Flexible processes, tailored information assessed for competence**	**meet competence threshold, providing valid informed consent**	**yes**
**CMOC 17**	**M1 Macro level health research sponsored by wealthy nations in low income countries**	**M3 Inflexible process Lengthy consent documentation, not tailored to those with low literacy levels**	**Inducement , poor understanding of study information, questionable validity of informed consent , exploitation**	**no**
**CMOC 18**	**M1 Macro level health research in resource-poor settings**	**M1 Skilled, culturally competent researchers delivered by trained researchers in obtaining valid consent from minors, with age-appropriate study information**	**free choice to participate**	**unclear**
**CMOC19**	**M1 Macro level regulations protect vulnerable populations from taking part**	**M2 Removing /restricting choice information not provided**	**removes freedom of choice**	**no**

For peer review only

CMOC 20	M2 Meso level researcher matching	M1 Skilled, culturally competent researchers researchers from similar backgrounds who speak the language , understand cultural circumstances	creates trust	unclea r
CMOC21	M1 Macro level informed consent in low-income countries	M1 Skilled, culturally competent researchers fieldworker trained in communication skills and utilising local language to share information, in bite-size chunks	benefits potential participants whom are educated as knowledge is better	unclea r
CMOC22	M2 Meso level research protocol design	M3 Inflexible process M2 Removing/restricting choice complex forms and communication barriers, lack of social presence in the community, lack of diversity amongst researchers	results in exclusion, experience of mistrust, feel exploited and that academic institutions are not committed to ethnic minority communities, suspicion of formal transaction	no
CMOC23	M2 Meso level community participation and ethical review boards willingness to adapt existing process	M4 Flexible processes, tailored information adaptation of resources, consent in different formats (incl multimedia),	creates trust, better conditions , assessment of comprehension & acceptability, cultural sensitivity promotes access, community	unclea r

For peer review only

			relationship building	
CMOC24	M2 Meso level<br>design of study for offenders with serious mental health problems	M4 Flexible processes, tailored information<br>careful description of study, how it is communicated and by whom, in conjunction with dialogue-type counselling techniques , for patients whom are deemed to be able to make a competent decision	reduces coercion and confusion of role as a patient as opposed to a potential participant	unclear
CMOC25	M1 Macro level<br>ethical guidelines for informed consent	M2 Removing choice/restricting choice<br>restrict operational flexibility	lack of cultural sensitivity and appreciation of family and cultural dynamics that affect decision-making	no
CMOC26	M1 Macro level<br>many African countries	M1 Skilled, culturally competent researchers<br>culturally appropriate delivery, taking into consideration social and cultural conventions, familiarity with local context of research	improved acceptability of the decision-making process	yes
CMOC27	M2 Meso level<br>M1 Micro level	M2 Removing/restricting choice	prevents inclusion, and	no

For peer review only

	research with patients with cognitive decline	family members, clinicians and care facility staff acting as gatekeepers	freedom of choice	
CMOC28	M2 Meso Level<br>M1 Micro level<br>research with patients with cognitive decline e.g. dementia	M4 Flexible processes, tailored information capacity assessment processes such as a MMSE score, as a way of determining capacity to consent and communication strategies tailored to meet needs of those with dementia	enables person to make own decision , whilst is a continuous check of willingness	yes
CMOC29	M2 Meso level<br>M1 Micro level<br>research with people with intellectual disability	M4 Flexible processes, tailored information adapting study materials and communication strategies during the informed consent process	builds rapport and engagement with participant and enables them to participate in the consent process to the best of their ability	yes

For peer review only

PRISMA 2020 Checklist

Section and Topic	Item #	Checklist item	Location where item is reported
**TITLE**			
Title	1	Identify the report as a systematic review.	N/A (realist review) RAMESES guidelines: http://www.ramesesproject.org/
**ABSTRACT**			
Abstract	2	See the PRISMA 2020 for Abstracts checklist.	N/A
**INTRODUCTION**			
Rationale	3	Describe the rationale for the review in the context of existing knowledge.	Page 5 (lines 109-111)
Objectives	4	Provide an explicit statement of the objective(s) or question(s) the review addresses.	Page 6 (lines 135-139)<br>Page 5 (lines 120-123)
**METHODS**			
Eligibility criteria	5	Specify the inclusion and exclusion criteria for the review and how studies were grouped for the syntheses.	Page 10 (line 229-231)
Information sources	6	Specify all databases, registers, websites, organisations, reference lists and other sources searched or consulted to identify studies. Specify the date when each source was last searched or consulted.	Page 9 (lines 206-207)
Search strategy	7	Present the full search strategies for all databases, registers and websites, including any filters and limits used.	Page 9 (lines 204-208,220-221)
Selection process	8	Specify the methods used to decide whether a study met the inclusion criteria of the review, including how many reviewers screened each record and each report retrieved, whether they worked independently, and if applicable, details of automation tools used in the process.	Page 9 (lines 209-212)<br>Page 10 (lines 229-231)
Data collection process	9	Specify the methods used to collect data from reports, including how many reviewers collected data from each report, whether they worked independently, any processes for obtaining or confirming data from study investigators, and if applicable, details of automation tools used in the process.	Pages 10-11 (lines 229-247)
Data items	10a	List and define all outcomes for which data were sought. Specify whether all results that were compatible with each outcome domain in each study were sought (e.g. for all measures, time points, analyses), and if not, the methods used to decide which results to collect.	N/A
	10b	List and define all other variables for which data were sought (e.g. participant and intervention characteristics, funding sources). Describe any assumptions made about any missing or unclear information.	N/A
Study risk of bias assessment	11	Specify the methods used to assess risk of bias in the included studies, including details of the tool(s) used, how many reviewers assessed each study and whether they worked independently, and if applicable, details of automation tools used in the process.	N/A
Effect measures	12	Specify for each outcome the effect measure(s) (e.g. risk ratio, mean difference) used in the synthesis or presentation of results.	N/A
Synthesis methods	13a	Describe the processes used to decide which studies were eligible for each synthesis (e.g. tabulating the study intervention characteristics and comparing against the planned groups for each synthesis (item #5)).	N/A

For peer review only

PRISMA 2020 Checklist

Section and Topic	Item #	Checklist item	Location where item is reported
	13b	Describe any methods required to prepare the data for presentation or synthesis, such as handling of missing summary statistics, or data conversions.	N/A
	13c	Describe any methods used to tabulate or visually display results of individual studies and syntheses.	N/A
	13d	Describe any methods used to synthesize results and provide a rationale for the choice(s). If meta-analysis was performed, describe the model(s), method(s) to identify the presence and extent of statistical heterogeneity, and software package(s) used.	N/A
	13e	Describe any methods used to explore possible causes of heterogeneity among study results (e.g. subgroup analysis, meta-regression).	N/A
	13f	Describe any sensitivity analyses conducted to assess robustness of the synthesized results.	N/A
Reporting bias assessment	14	Describe any methods used to assess risk of bias due to missing results in a synthesis (arising from reporting biases).	N/A
Certainty assessment	15	Describe any methods used to assess certainty (or confidence) in the body of evidence for an outcome.	N/A
**RESULTS**			
Study selection	16a	Describe the results of the search and selection process, from the number of records identified in the search to the number of studies included in the review, ideally using a flow diagram.	Page 9 (line 220 (figure 3))
	16b	Cite studies that might appear to meet the inclusion criteria, but which were excluded, and explain why they were excluded.	N/A
Study characteristics	17	Cite each included study and present its characteristics.	N/A
Risk of bias in studies	18	Present assessments of risk of bias for each included study.	N/A
Results of individual studies	19	For all outcomes, present, for each study: (a) summary statistics for each group (where appropriate) and (b) an effect estimate and its precision (e.g. confidence/credible interval), ideally using structured tables or plots.	N/A
Results of syntheses	20a	For each synthesis, briefly summarise the characteristics and risk of bias among contributing studies.	N/A
	20b	Present results of all statistical syntheses conducted. If meta-analysis was done, present for each the summary estimate and its precision (e.g. confidence/credible interval) and measures of statistical heterogeneity. If comparing groups, describe the direction of the effect.	N/A
	20c	Present results of all investigations of possible causes of heterogeneity among study results.	N/A
	20d	Present results of all sensitivity analyses conducted to assess the robustness of the synthesized results.	N/A

For peer review only

PRISMA 2020 Checklist

Section and Topic	Item #	Checklist item	Location where item is reported
Reporting biases	21	Present assessments of risk of bias due to missing results (arising from reporting biases) for each synthesis assessed.	Page 22 (lines 494-496)
Certainty of evidence	22	Present assessments of certainty (or confidence) in the body of evidence for each outcome assessed.	N/A
**DISCUSSION**			
Discussion	23a	Provide a general interpretation of the results in the context of other evidence.	Page 19 (lines 422-427)
	23b	Discuss any limitations of the evidence included in the review.	N/A
	23c	Discuss any limitations of the review processes used.	Page 22 (lines 490-493)
	23d	Discuss implications of the results for practice, policy, and future research.	Page 17-19 (lines 418-419 (Table 1))
**OTHER INFORMATION**			
Registration and protocol	24a	Provide registration information for the review, including register name and registration number, or state that the review was not registered.	Not registered
	24b	Indicate where the review protocol can be accessed, or state that a protocol was not prepared.	https://systematicreviewsjournal.biomedcentral.com/articles/10.1186/s13643-021-01652-2
	24c	Describe and explain any amendments to information provided at registration or in the protocol.	N/A
Support	25	Describe sources of financial or non-financial support for the review, and the role of the funders or sponsors in the review.	Page 24 (lines 549-556)
Competing interests	26	Declare any competing interests of review authors.	Page 24 (line 559)
Availability of data, code and other materials	27	Report which of the following are publicly available and where they can be found: template data collection forms; data extracted from included studies; data used for all analyses; analytic code; any other materials used in the review.	Page 7 Supplementary file 1 (lines 170-171)<br>Page 9 Supplementary file 2 (lines 205)<br>Page 10 Supplementary file 3 (line 229-230)<br>Page 13 Supplementary file 4 (line 299-300)

From: Page MJ, McKenzie JE, Bossuyt PM, Boutron I, Hoffmann TC, Mulrow CD, et al. The PRISMA 2020 statement: an updated guideline for reporting systematic reviews. BMJ 2021;372:n71. doi: 10.1136/bmj.n71

For more information, visit: http://www.prisma-statement.org/

For peer review only
